# Long-term Outcomes of Bevacizumab and Chemoradiation for Locoregionally Advanced Nasopharyngeal Carcinoma: A Nonrandomized Controlled Trial

**DOI:** 10.1001/jamanetworkopen.2023.16094

**Published:** 2023-06-02

**Authors:** Nancy Y. Lee, Jonathan Harris, John Kim, Adam Garden, James Mechalakos, David G. Pfister, Anthony T.C. Chan, Kenneth Hu, A Dimitrios Colevas, Steven Frank, George Shenouda, Voichita Bar-Ad, John N. Waldron, Paul M. Harari, Adam Raben, Pedro Torres-Saavedra, Quynh-Thu Le

**Affiliations:** 1 Department of Radiation Oncology, Memorial Sloan Kettering Cancer Center, New York, New York; 2 Statistics and Data Management Center, NRG Oncology, American College of Radiology, Philadelphia, Pennsylvania; 3 Department of Radiation Oncology, Princess Margaret Cancer Centre, University Health Network, University of Toronto, Toronto, Ontario, Canada; 4 Department of Radiation Oncology, MD Anderson Cancer Center, Houston, Texas; 5 Department of Medical Physics, Memorial Sloan Kettering Cancer Center, New York, New York; 6 Department of Medicine Head and Neck Service, Memorial Sloan Kettering Cancer Center, New York, New York; 7 Department of Clinical Oncology, Chinese University of Hong Kong, Shatin, Hong Kong; 8 Department of Radiation Oncology, New York University Langone Medical Center, New York, New York; 9 Accrual for State University of New York Health Science Center, Brooklyn, New York; 10 Department of Medicine, Stanford University, Stanford, California; 11 Accruals for University of California, San Francisco; 12 Department of Radiation Oncology, McGill University, Montreal, Quebec, Canada; 13 Department of Radiation Oncology, Thomas Jefferson University, Philadelphia, Pennsylvania; 14 Department of Human Oncology, University of Wisconsin School of Medicine and Public Health, Madison; 15 Helen F. Graham Cancer Center, Christiana Care Health System, Newark, Delaware; 16 Department of Radiation Oncology, Stanford University, Stanford, California

## Abstract

**Importance:**

The long-term outcomes associated with adding bevacizumab, a vascular endothelial growth factor inhibitor, to standard chemoradiation have continued to be favorable for a group of patients with locoregionally advanced nasopharyngeal carcinoma (NPC).

**Objective:**

To assess long-term toxic effects and clinical outcomes associated with chemotherapy, radiation therapy (RT), and bevacizumab for NPC.

**Design, Setting, and Participants:**

This single-arm phase II nonrandomized controlled trial was conducted by the National Cancer Trials Network group and NRG Oncology (formerly Radiation Therapy Oncology Group), with accrual from December 13, 2006, to February 5, 2009, and data analysis from June 26 to July 1, 2019. The study was conducted at 19 cancer centers with a median (IQR) follow-up of 9.0 (7.7-9.3) years. Included patients were adults (aged ≥18 years) with NPC that was World Health Organization (WHO) histologic grade I to IIb or III, American Joint Committee on Cancer stage IIB or greater, and with or without lymph node involvement.

**Interventions:**

Patients received 3 cycles of bevacizumab (15 mg/kg) concurrently with standard cisplatin (100 mg/m^2^) and RT (69.96 Gy) followed by 3 cycles of adjuvant bevacizumab (15 mg/kg) given concurrently with cisplatin (80 mg/m^2^) and fluorouracil (1000 mg/m^2^/d).

**Main Outcomes and Measures:**

The primary end point was grade 4 hemorrhage or grade 5 adverse events in the first year. Secondary end points were locoregional progression–free (LRPF) interval, distant metastasis–free (DMF) interval, progression-free survival (PFS), overall survival (OS), and other adverse events. Long-term toxic effects and clinical outcomes were reported due to the limited follow-up in the initial report for this trial and the importance of long-term outcomes when combining bevacizumab with chemoradiation.

**Results:**

Among 46 patients with NPC who were enrolled, 44 patients were analyzed (29 males [65.9%]; 23 Asian [52.3%], 2 Black [4.5%], and 16 White [36.4%]; 38 not Hispanic [86.4%]; median [IQR] age, 48.5 [39.0-56.0] years). There were 33 patients with a Zubrod performance status of 0, indicating that they were fully functional and asymptomatic (75.0%); 32 patients with a WHO histologic grade of IIb or III (72.7%); and 39 patients with stage III or IVB disease (88.6%). Among analyzed patients, 42 individuals received radiation therapy of 69.96 Gy or greater (95.5%; dose range, 65.72-70.00 Gy); 30 patients received 3 cycles of cisplatin (68.2%) with RT, and 31 patients received 3 cycles of bevacizumab with RT (70.5%); this was followed by 3 cycles of adjuvant cisplatin in 21 patients (47.7%), fluorouracil in 24 patients (54.5%), and bevacizumab in 23 patients (52.3%). No grade 4 hemorrhage or grade 5 AEs were reported in the first year or thereafter. Late grade 3 AEs occurred in 16 patients (36.4%), including 7 patients with dysphagia (15.9%), 6 patients with hearing impairment (13.6%), and 2 patients with dry mouth (4.5%). The 1- and 5-year rates of feeding tube use were 5 of 41 patients (12.2%) and 0 of 27 patients, respectively. There were 19 patients (43.2%) who progressed or died without disease progression (6 patients with locoregional progression [13.6%], 8 patients with distant progression [18.2%], and 5 patients who died without progression [11.4%]). The 5- and 7-year rates were 79.5% (95% CI, 67.6%-91.5%) and 69.7% (95% CI, 55.9%-83.5%) for OS, 61.2% (95% CI, 46.8%-75.6%) and 56.3% (95% CI, 41.5%-71.1%) for PFS, 74.9% (95% CI, 61.4%-86.6%) and 72.3% (95% CI, 58.4%-84.7%) for LRPF interval, and 79.5% (95% CI,66.4%-90.0%) for both times for DMF interval. Among 13 patients who died, death was due to disease in 8 patients (61.5%).

**Conclusions and Relevance:**

In this nonrandomized controlled trial, no grade 4 hemorrhage or grade 5 AEs were reported in the first year or thereafter among patients with NPC receiving bevacizumab combined with chemoradiation. The rate of distant metastasis was low although 89% of patients had stage III to IVB disease, suggesting that further investigation may be warranted.

**Trial Registration:**

ClinicalTrials.gov Identifier: NCT00408694

## Key Points

**Question:**

Can bevacizumab be safely combined with chemoradiation in the treatment of nasopharyngeal cancer?

**Findings:**

In this nonrandomized controlled trial of 44 patients with locoregionally advanced nasopharyngeal cancer, the addition of bevacizumab to standard chemoradiation was not associated with excessive or unwarranted toxic effects.

**Meaning:**

These findings suggest that the addition of bevacizumab to chemoradiation for nasopharyngeal cancer may be safe and tolerable.

## Teaser

This nonrandomized controlled trial investigates the association of bevacizumab added to standard chemoradiation with adverse events in patients with nasopharyngeal cancer.

## Introduction

There are currently 2 standard treatment options for locoregionally advanced nasopharyngeal cancer (LA-NPC). Patients can be treated with induction chemotherapy followed by concurrent chemoradiotherapy (CCRT) or CCRT followed by adjuvant chemotherapy.^1,2,3,4^ There were debates concerning whether adjuvant chemotherapy was necessary after CCRT for these tumors; however, findings from the most recent updated NPC meta-analysis of chemotherapy (MAC-NPC) meta-analysis^5^ suggest that concurrent CCRT alone may be insufficient for LA-NPC. Patients may even need additional adjuvant therapy despite having received induction chemotherapy.^5,6,7,8^ This standard did not change despite the introduction of intensity-modulated radiation therapy (IMRT).^9^ With IMRT, high locoregional control can be achieved for NPC, but distant recurrence remains the predominant failure resulting in patient death. These findings suggest that more effective systemic therapy is needed to further improve overall survival (OS) for NPC.^5,6^

Overexpression of vascular endothelial growth factor (VEGF) A has been associated with a poor prognosis for NPC.^10^ Patients with NPC and increased levels of VEGF also have higher likelihoods of tumor recurrence and distant metastases and worse OS. A preclinical study^11^ in a head and neck squamous cell carcinoma model system found that antiangiogenic strategies (VEGF inhibition) in combination with radiation were associated with favorable tumor response outcomes. The Radiation Therapy Oncology Group (RTOG; now NRG Oncology) launched this phase 2 study (RTOG 0615) to assess the safety and tolerability of the addition of bevacizumab, a monoclonal antibody directed against VEGF, to CCRT and adjuvant chemotherapy. The study also assessed 1- and 2-year rates of locoregional failure, distant metastasis, progression-free survival (PFS), and OS.^12^ Indeed, preliminary results from this combination were encouraging, with 2-year distant metastasis–free (DMF) interval and OS rates of approximately 90% for a large group of patients with locoregionally advanced NPC. No unwarranted adverse events, including hemorrhage, were observed, and furthermore, it was deemed safe to deliver 6 total cycles of bevacizumab in combination with CCRT (3 concurrent cycles and 3 adjuvant cycles).^12^ This study reports the long-term results of this phase II multicenter trial. This analysis is timely given the renewed interest in adding VEGF inhibition, which may be associated with enhanced anticancer outcomes for immunotherapy.

## Methods

Patients signed written informed consent for this nonrandomized controlled study. The study was approved by the National Cancer Institute and participating centers’ institutional review boards; it is registered at ClinicalTrials.gov (NCT00408694). A copy of the protocol can be found in Supplement 1. This study followed the Transparent Reporting of Evaluations With Nonrandomized Designs (TREND) reporting guideline.

### Study Objectives and Patient Eligibility

RTOG 0615 is a nonrandomized multicenter phase II trial at 19 cancer centers that included patients with previously untreated stage IIB through IVB NPC (according to the American Joint Committee on Cancer Staging Manual, 6th Edition [AJCC 6]). Participating centers were the Beth Israel Medical Center, Memorial Sloan Kettering Cancer Center, Stanford University Medical Center, City of Hope Medical Center, Chinese University of Hong Kong, University Of Texas MD Anderson Cancer Center, MD Anderson Cancer Center-Orlando, McGill University, Thomas Jefferson University Hospital, University of Medicine and Dentistry of New Jersey-New Jersey Medical School, Princess Margaret Hospital, University of Wisconsin Hospital, Schiffler Cancer Center, Medical College of Wisconsin, Radiological Associates of Sacramento, University Of Western Ontario, University of Pittsburgh Medical Center-Shadyside Hospital, St. John’s Hospital Cancer Institute, and Christiana Care Health Services, Inc. Patients with Zubrod performance status of 0 (indicating fully functioning and asymptomatic) to 1 (indicating symptomatic but completely ambulatory) who met criteria for blood counts and other tests (eg, white blood cell [WBC] count ≥4000/μl; platelet count ≥100 ×10^3^/μl; serum creatinine ≤1.5 mg/dL) were eligible for this study (to convert platelet count to ×10^9^ per liter, multiply by 1.0; creatinine to micromoles per liter, multiply by 88.4; white blood cell count to ×10^9^ per liter, multiply by 0.001). Patients younger than age 18 years, those with a prior (within 3 years) or synchronous malignant neoplasm other than nonmelanoma skin cancer, and those with T stage 1 or 2 disease with involvement of retropharyngeal lymph nodes only (N1) were excluded. Patients with tumors close to major blood vessels were not excluded from this study. However, patients who had major bleed within 1 month of enrollment were excluded from this study. Race and ethnicity were self-reported. Race options were American Indian or Alaska Native, Asian, Black or African American, Native Hawaiian or other Pacific Islander, White, more than 1 race, and unknown. Ethnicity options were Hispanic or Latino, not Hispanic or Latino, and unknown. Race and ethnicity were assessed to obtain the demographic background of the study population.

Standard of care pretreatment evaluations consisted of history collection and physical, dental, nutritional, audiogram, and laboratory studies. Magnetic resonance imaging (MRI) of the nasopharynx or neck was required unless there was a contraindication (ie, use of a pacemaker), in which case a computed tomography (CT) scan of the nasopharynx or neck was required. Additional tests to evaluate the extent of disease included chest x-ray; tests of alkaline phosphatase level, liver function, and lactate dehydrogenase level; and liver and bone scans when indicated. Positron emission tomography (PET) was optional at the time of this protocol.

### Radiation, Chemotherapy, and Bevacizumab Guidelines

For radiation planning, fusion of MRI or PET/CT scans with treatment-planning CT images was encouraged to accurately define the gross tumor volume, which consisted of the primary disease in the nasopharynx and nodes greater than 1 cm in diameter or nodes with necrotic centers. Areas at risk for subclinical spread of disease included the entire nasopharynx, retropharyngeal nodal regions, clivus, skull base, pterygoid fossae parapharyngeal fat (coverage of trigeminal nerve V3), sphenoid sinus, and posterior third of the nasal cavity or maxillary sinuses, including the pterygopalatine fossae (coverage of trigeminal nerve V2) and bilateral neck I through V lymph nodal regions. Level I nodal regions were not included in the N0 neck. IMRT was delivered in 33 fractions using a simultaneous integrated boost technique in which the planning target volume (PTV) of the gross disease received radiation doses as follows: PTV_70_ received 70 Gy in 2.12 Gy/fraction, high-risk subclinical disease (PTV_59.4_) received 59.4 Gy in 1.8Gy/fraction, and lower-risk subclinical disease (PTV_54_) received 54 Gy at 1.64 Gy/fraction. Radiation therapy was administered daily, Monday through Friday for 33 days. Lower neck nodes could be included in extended IMRT fields or irradiated with matching conventional anterior-posterior fields. In the latter scenario, a dose of 50.4 Gy was administered at 1.8 Gy/fraction.

Bevacizumab (15 mg/kg) was given concurrently with cisplatin (100 mg/m^2^) on days 1, 22, and 43, followed by adjuvant bevacizumab (15 mg/kg) and cisplatin (80 mg/m^2^) on day 1 and fluorouracil (protracted venous infusion, 1000 mg/m^2^/d) on days 1 to 4 every 4 weeks × 3 cycles. Adjuvant therapy started 3 weeks after CCRT and occurred on days 64 to 67, 85 to 88, and 106 to 109 after radiation. For more details, please refer to prior publication.^12^

### Follow-up and End Points

Patients underwent weekly examinations during CCRT. Follow-up evaluations occurred every 3 months during the first 2 years, every 6 months during years 3 to 5, and then annually. Adverse events were graded per National Cancer Institute Common Terminology Criteria for Adverse Events version 3.0. Adverse events scored as definitely, probably, or possibly associated with protocol treatment (or with unknown association) were considered treatment associated.

#### Primary End Point and Sample Size Derivation

The primary end point was treatment-associated grade 4 hemorrhage or treatment-associated grade 5 adverse events in the first year after the start of treatment. The trial used a Fleming 2-stage design with 14 and 42 patients in the first and second stage (0.14 one-sided α and 83% power; acceptable and unacceptable rates of 5% and 15%, respectively); more details were given in the initial publication.^12^

#### Secondary End Points

Secondary end points were treatment-associated grade 4 hemorrhage or treatment-associated grade 5 adverse events after the first year, death during treatment or within 30 days of treatment completion (regardless of the cause of death), other grade 3 to 5 adverse events, feeding tube use rates, treatment tolerability (for each treatment component, concurrent and adjuvant), locoregional progression–free (LRPF) interval, DMF interval, PFS, and OS. Long-term treatment–associated AEs and clinical outcomes (OS, PFS, LRPF interval, and DMF interval), including 5- and 7-year rates, that were not specified in the protocol are reported for this study.

### Statistical Analysis

For the concurrent phase, tolerability was measured by the percentage of patients receiving 2 or more cycles of cisplatin and bevacizumab with RT; it was scored by study chairs (N.Y.L., J.K., and D.G.P.) as no variation or minor variation for tumor volume contouring score and organs at risk contouring score. For the adjuvant phase, tolerability was measured by the percentage of patients receiving 2 or more cycles of cisplatin, fluorouracil, and bevacizumab. If either component had a tolerability rate of less than 50%, that component would not be recommended for further study without modification. Failure for LRPF rate was local or regional progression or recurrence or death due to study cancer or unknown causes with an undocumented site of failure. Failure for DMF rate was distant metastasis. Failure for PFS was local, regional, or distant recurrence or progression, or death due to any cause. Failure for OS was death due to any cause. All time-to-event end points were defined from the time of registration to failure; patients without failure were censored at the last follow-up. LRPF and DMF rates were estimated by subtracting cumulative incidence estimates from 100%; death without failure was considered a competing risk for both end points. PFS and OS rates were estimated by the Kaplan-Meier method. CIs for 5- and 7-year OS and PFS rates were calculated using Greenwood variance and normal approximation; LRPF and DMF interval rates were computed using a log-log transformation and normal approximation. Acute and late toxic effect periods were defined as 90 days or less vs greater than 90 days, respectively, after the end of treatment. CIs for treatment-associated AE rates across time were computed using the Wald method. Eligible patients who started protocol treatment were included in the analysis. DMF survival rates (with failure as distant metastasis or death) at 5 and 7 years are also presented as an unplanned sensitivity analysis to allow comparison with other published trials. Data were analyzed using SAS statistical software version 9.4 (SAS Institute), with analysis from June 26 to July 1, 2019.

## Results

### Patient Characteristics and Treatment Compliance

**Figure 1.**
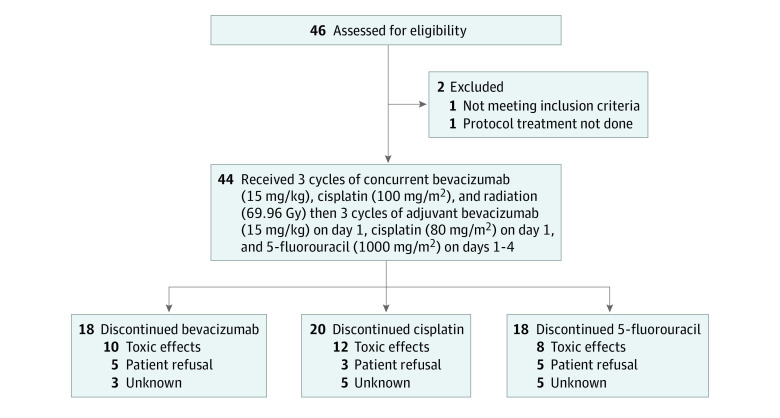
Study Flowchart

Among 46 patients with NPC enrolled in the study from December 13, 2006, to February 5, 2009, 44 patients (29 males [65.9%]; 23 Asian [52.3%], 2 Black [4.5%], and 16 White [36.4%]; 38 not Hispanic [86.4%]; median [IQR] age, 48.5 [39.0-56.0] years) were eligible for analysis (1 patient did not start protocol therapy, and 1 patient was ineligible due to low WBC count; see patient flowchart in Figure 1). There were 33 patients with a Zubrod score of 0 (75.0%), 32 patients with a World Health Organization histologic grade of IIb or III (72.7%), and 39 patients with stage III or IVB disease (88.6%). Table 1 lists pretreatment patient and tumor characteristics. With changes in the staging system since the completion of this study, 10 patients may have been downstaged. Details of treatment administered have been published previously.^12^ In brief, all but 1 eligible patient (43 patients [97.7%]) received IMRT, and all but 2 patients (42 patients [95.5%]) received the prescribed radiation dose of 69.96 Gy or greater (dose range, 65.72-70.00 Gy). All patients received at least 2 cycles of cisplatin concurrent with RT, and 1 patient received less than 2 cycles of bevacizumab concurrent with IMRT (2.3%); 30 patients received 3 cycles of cisplatin (68.2%) with RT, and 31 patients received 3 cycles of bevacizumab with radiation therapy (70.5%); this was followed by 3 cycles of adjuvant cisplatin in 21 patients (47.7%), fluorouracil in 24 patients (54.5%), and bevacizumab in 23 patients (52.3%). Concurrent and adjuvant phases were both tolerable in 30 patients (68.2%).

**Table 1.** Pretreatment Patient Characteristics

Characteristic	Patients, No. (%) (N = 44)
Age, median (IQR), y	48.5 (39.0-56.0)
Sex	
Male	29 (66)
Female	15 (34)
Race	
Asian	23 (52)
Black or African American	2 (5)
White	16 (36)
>1 Race^a^	2 (5)
Unknown	1 (2)
Ethnicity	
Hispanic or Latino	3 (7)
Not Hispanic or Latino	38 (86)
Unknown	3 (7)
Zubrod performance status	
0	33 (75)
1	11 (25)
Primary tumor site	
Nasopharynx not otherwise specified	43 (98)
Posterior superior wall	1 (2)
WHO histologic grade	
I	4 (9)
IIa or II	8 (18)
IIb or III	32 (73)
T stage	
T1	12 (27)
T2a	4 (9)
T2b	3 (7)
T3	15 (34)
T4	10 (23)
N stage	
N0	5 (11)
N1	8 (18)
N2	26 (59)
N3a	2 (5)
N3b	3 (7)
AJCC stage	
IIB	5 (11)
III	24 (55)
IVA	10 (23)
IVB	5 (11)

Abbreviations: AJCC, American Joint Committee on Cancer; WHO, World Health Organization.

^a^ There was an option for more than 1 self-reported race in the patient clinical record.

### Treatment Outcome Data

**Figure 2.**
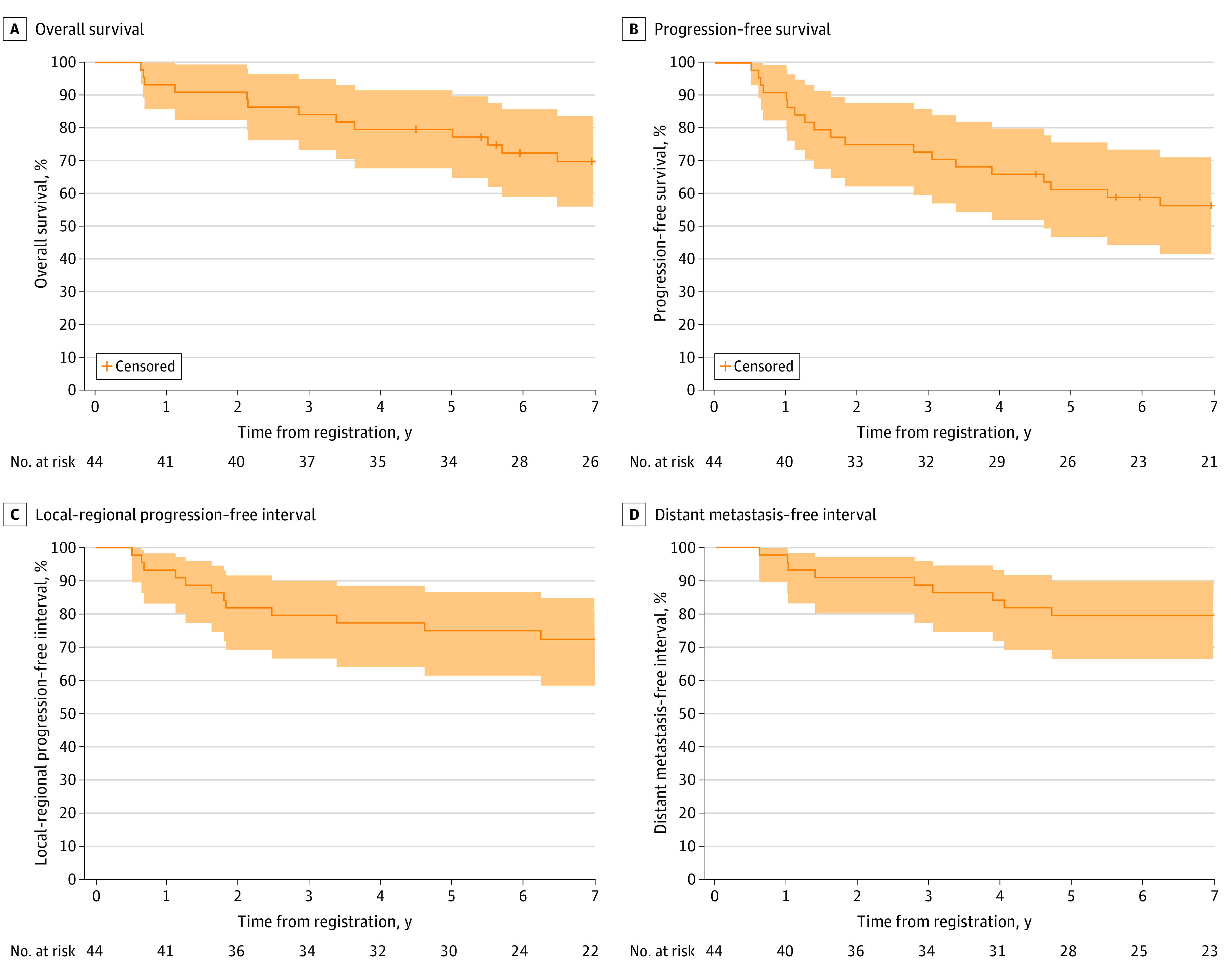
Estimates of Survival Rates Shaded areas indicate pointwise 95% CIs.

Median (IQR) follow-up for surviving patients was 9.0 (7.7-9.3) years compared with 2.5 (2.1-2.9) years for the initial report. After the initial report, 7 additional deaths were reported, for a total of 13 deaths (29.5%). Estimated 5- and 7-year OS rates were 79.5% (95% CI, 67.6%-91.5%) and 69.7% (95% CI, 55.9%-83.5%), respectively. After the initial report, 7 additional PFS events were reported, for a total of 19 events (43.2%; 6 patients with locoregional progression [13.6%], 8 patients with distant progression [18.2%], and 5 patients who died without progression [11.4%]). The first failure among new events was locoregional progression in 2 patients, distant metastasis in 3 patients, death due to other causes in 1 patient, and death due to unknown causes in 1 patient. Estimated 5- and 7-year PFS rates were 61.2% (95% CI, 46.8%-75.6%) and 56.3% (95% CI, 41.5%-71.1%), respectively. After the initial report, 5 additional LRPF events (4 locoregional progressions and 1 death due to unknown causes) were reported, for a total of 12 events (27.3%; 8 locoregional progressions, 2 deaths due to study cancer, and 2 deaths due to unknown causes). Estimated 5- and 7-year LRPF interval rates were 74.9% (95% CI, 61.4%-86.6%) and 72.3% (95% CI, 58.4%-84.7%), respectively. After the initial report, 4 additional DMF events were reported, for a total of 9 events (20.5%). Estimated 5- and 7-year DMF interval rates were both 79.5% (95% CI, 66.4%-90.0%). Estimated 5- and 7-year DMF survival rates were 65.8% (95% CI, 51.8%-79.9%) and 60.9% (95% CI, 46.4%-75.5%). Figure 2 shows estimated OS, PFS, LRPF interval, and DMF interval rates. Of 19 PFS failure events, 6 events (31.6%) were locoregional progression, 8 events (42.1%) were distant progression, and 5 events (26.3%) were death. Of 13 deaths, 8 deaths (61.5%, or 18.2% of all patients) were due to study cancer, 1 death (7.7%) was due to other causes, and 4 deaths (30.8%) were due to unknown causes.

### Toxic Effects Data

No grade 4 hemorrhages or grade 5 adverse events in the first year were reported in the prior publication,^12^ and no such adverse events after the first year were recorded in this update. There were 33 patients (75.0%) with grade 3 and 1 patient (2.3%) with grade 4 acute radiation mucositis, and there were 6 patients (13.6%) with acute grade 3 radiation dermatitis. Overall, there were 40 patients with grade 3 to 4 toxic effects (90.9%).

**Figure 3.**
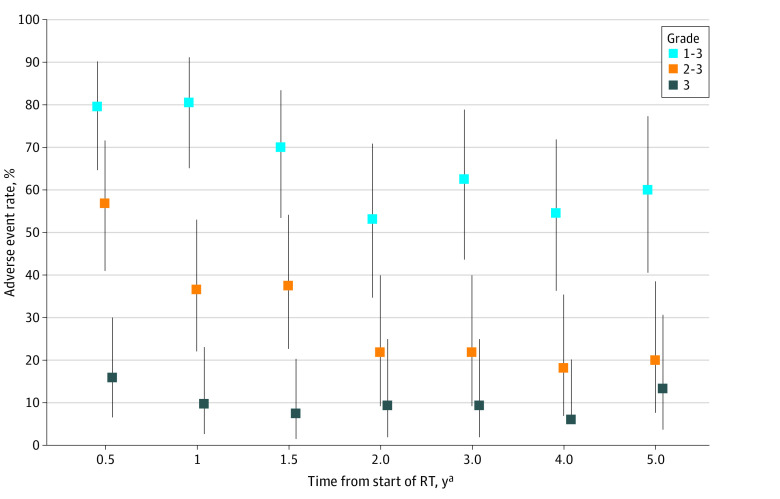
Estimates of Treatment-Associated Late Adverse Event Rates Over Time RT indicates radiation therapy; whiskers, 95% CIs. ^a^Events occurring in a window of 3 months before or after the scheduled follow-up period were included.

The most frequently reported late adverse event grades were grade 3, occurring in 16 patients (36.4%); grade 2, occurring in 16 patients (36.4%); and grade 1, occurring in 9 patients (20.5%). There were late grade 3 reports of dysphagia in 7 patients (15.9%), hearing impairment in 6 patients (13.6%), dry mouth in 2 patients (4.5%), and neuralgia not otherwise specified in 2 patients (4.5%). No other late grade 3 adverse event was reported in more than 1 patient. Late adverse event rates over time are summarized in Figure 3 and Table 2. At 1, 2, and 5 years after treatment, 4 of 41 patients (9.8%), 3 of 32 patients (9.4%), and 4 of 30 patients (13.3%) had a grade 3 adverse event, respectively. Among all patients, 4 patients (9.1%) underwent use of a prophylactic feeding tube. Feeding tubes were used in 5 of 41 patients (12.2%) at 1 year, 2 of 37 patients (5.4%) at 2 years, 1 of 30 patients (3.3%) at 3 years, 1 of 29 patients (3.4%) at 4 years, and 0 of 27 patients at 5 years.

**Table 2.** Grade 3 Treatment-Associated Late Adverse Event Rates by Category and Term^a^

Event category or term	Patients, No. (%) (N = 44)							
	Any (n = 44)	6 mo (n = 44)	1 y (n = 41)	18 mo (n = 40)	2 y (n = 32)	3 y (n = 32)	4 y (n = 33)	5 y (n = 30)
Any event	16 (36)	7 (16)	4 (10)	3 (8)	3 (9)	3 (9)	2 (6)	4 (13)
Auditory/ear	6 (14)	1 (2)	2 (5)	2 (5)	1 (3)	3 (9)	2 (6)	3 (10)
Hearing disability	1 (2)	1 (2)	1 (2)	1 (3)	0	0	0	0
Hearing impaired	6 (14)	0	1 (2)	1 (3)	1 (3)	2 (6)	2 (6)	3 (10)
Otitis media serous NOS	1 (2)	0	0	0	0	1 (3)	0	0
Constitutional symptoms	2 (5)	1 (2)	1 (2)	0	0	0	0	0
Constitutional symptoms—other	1 (2)	1 (2)	0	0	0	0	0	0
Fatigue	1 (2)	0	1 (2)	0	0	0	0	0
Gastrointestinal	9 (20)	5 (11)	1 (2)	0	0	0	0	0
Dehydration	1 (2)	1 (2)	0	0	0	0	0	0
Dry mouth	2 (5)	0	0	0	0	0	0	0
Dysphagia	7 (16)	5 (11)	1 (2)	0	0	0	0	0
Radiation mucositis	1 (2)	1 (2)	0	0	0	0	0	0
Infection	1 (2)	0	0	0	1 (3)	0	0	0
Infection—other	1 (2)	0	0	0	1 (3)	0	0	0
Musculoskeletal/soft tissue	1 (2)	1 (2)	0	0	0	0	0	0
Muscle weakness NOS	1 (2)	1 (2)	0	0	0	0	0	0
Neurology	2 (5)	1 (2)	0	1 (3)	1 (3)	0	0	0
Neurology other	1 (2)	0	0	0	0	0	0	0
Peripheral motor neuropathy	1 (2)	1 (2)	0	1 (3)	1 (3)	0	0	0
Peripheral sensory neuropathy	1 (2)	1 (2)	0	0	0	0	0	0
Pain	2 (5)	2 (5)	0	0	0	0	0	0
Neuralgia NOS	2 (5)	2 (5)	0	0	0	0	0	0
Vascular	2 (5)	0	0	0	0	0	0	1 (3)
Peripheral ischemia	1 (2)	0	0	0	0	0	0	1 (3)
Vessel injury—artery: carotid	1 (2)	0	0	0	0	0	0	0

Abbreviation: NOS, not otherwise specified.

^a^ Treatment associated is defined as definitely, probably, or possibly associated with the protocol treatment. The late period is defined as more than 90 days after the end of treatment. Time points were measured from the end of treatment, with a window of 3 months before or after the given time. Adverse events were graded with National Cancer Institute Common Terminology Criteria for Adverse Events version 3.0.

## Discussion

Findings from our initial report^12^ suggested that it was feasible to add bevacizumab to chemoradiation for LA-NPC without an associated compromise in the delivery of standard chemoradiation or adjuvant chemotherapy. With an updated median follow-up for surviving patients with NPC of 9.0 years, this nonrandomized controlled trial found promising estimated 5- and 7-year DMF interval rates of 79.5%, translating to 5- and 7-year OS rates of 79.5% and 69.7%, respectively. Furthermore, no other grade 3 or 4 hemorrhages were observed despite initial concern about such toxic effects. The reported grade 3 to 4 toxic effect rate of 90.9% in this series was slightly higher than the induction gemcitabine and cisplatin NPC trial’s findings of 75.7% for grade 3 to 4 toxic effects rates.^2^ Although data on this outcome was not collected in this study, bevacizumab has been found to be associated with hypertension and proteinuria^12^; patients should therefore be assessed thoroughly prior to administration of bevacizumab. Additionally, there was no excessive report in our study of unexpected late effects despite longer follow-up. Given that distant recurrence remains the predominant cause of patient death for NPC (approximately 20%),^5^ more effective systemic therapy or its combinations may be needed to further improve OS.

Immunotherapy (ie, programmed cell death 1 protein [PD1] inhibitors), has shown activity and promise for recurrent and metastatic NPC. Several phase II nonrandomized controlled trials^13,14,15^ have found overall response rates of approximately 20% for recurrent and metastatic NPC treated with immune-checkpoint inhibitors. The Keynote-028 study^15^ included patients with NPC among whom prior standard systemic therapy had failed. The overall response rate was impressive at 25.9% (95% CI, 11.1%**-**46.3%), with 26% of patients experiencing partial response and 52% of patients experiencing stable disease. Nivolumab was administered among patients with recurrent or metastatic NPC receiving substantial pretreatment.^13^ In 44 evaluable patients, the objective response rate (ORR) was 20.5%, the 1-year OS rate was 59% (95% CI, 44.3%-78.5%), and the 1-year PFS rate was 19.3% (95% CI, 10.1%-37.2%). Atezolizumab (an anti–programmed cell death 1 ligand 1 [PD-L1] drug) also showed promising efficacy for patients with NPC (13% of patients) in a phase I clinical trial. Among 32 patients, the ORR was 22%, with a median (range) PFS of 2.6 (0.5-48.4) months and a median (range) OS of 6.0 (0.5-51.6) months.^14^

Phase III randomized clinical trials^16,17^ have shown further improvement in the response rate and PFS when PD1 inhibitors were added to gemcitabine and cisplatin vs gemcitabine and cisplatin for first-line recurrent or metastatic NPC. With the addition of camrelizumab to standard chemotherapy, the median PFS was 10.8 months vs 6.9 months without camrelizumab (hazard ratio [HR], 0.51; 95% CI, 0.37-0.69; 1-sided *P* < .001). The median duration of response was also longer in the group with camrelizumab plus chemotherapy vs the group with placebo plus chemotherapy (HR, 0.48; 95% CI, 0.34-0.68). Preliminary data also suggest an OS improvement in patients who received camrelizumab (HR, 0.67; 95% CI, 0.41-1.11).^16^ Similarly, in the JUPITER-2 phase III randomized clinical trial,^17^ when toripalimab (a PD1 inhibitor) was added to cisplatin and gemcitabine, there was a significant increase in PFS per the Response Evaluation Criteria in Solid Tumours (RECIST) guideline version 1.1; most notably, the increase in 2-year OS was 77.8% with toripalimab vs 63.3% without it, translating to a 40% reduction in risk of death (HR, 0.60; 95% CI, 0.364-0.997, *P* = .0462). Although these results were encouraging, nearly 90% of patients experienced grade 3 or 4 toxic effect, attributed mainly to chemotherapy.^16,17^ This suggest that an opportunity may exist to replace chemotherapy with biologic-targeted therapy, such as bevacizumab, with lower rates of toxic effects. These patients with NPC would have already undergone a platinum-containing regimen, including induction, concurrent, or adjuvant regiments, so changing to targeted therapy like bevacizumab, an active drug, is a logical approach. The addition of PD1 inhibition to VEGF inhibition may be associated with further reductions in the rate of distance recurrence and further improvement in OS.

Combining anti-VEGF with anti–PD1 and PDL1 therapies may be associated with further improvement in efficacy by reversing VEGF-mediated immunosuppression and promoting T cell infiltration in tumors. This combination strategy was found to be effective in hepatocellular carcinoma, kidney cell cancer, lung cancer, and head and neck squamous cell carcinoma.^18,19,20,21,22,23^ In hepatocellular carcinoma, atezolizumab selectively targets PD-L1 to prevent interaction with receptors PD-1 and B7-1, thus reversing T cell suppression; bevacizumab is a monoclonal antibody that targets VEGF, inhibits angiogenesis and tumor growth, and reduces VEGF-mediated immunosuppression within the tumor and its microenvironment. IMbrave150 was a global, multicenter, open-label phase III randomized clinical trial investigating the safety and efficacy of atezolizumab plus bevacizumab compared with sorafenib in patients with unresectable hepatocellular carcinoma who had not previously received systemic therapy.^20^ In patients with unresectable hepatocellular carcinoma, atezolizumab combined with bevacizumab resulted in better OS and PFS outcomes vs sorafenib alone.^20^ In nonsquamous non–small cell lung cancer in a phase III randomized clinical trial,^21^ the addition of atezolizumab to bevacizumab plus chemotherapy improved PFS vs chemotherapy and bevacizumab regardless of PD-L1 expression or *EGFR* or *ALK* genetic alteration status (HR, 0.62; *P* < .001). In a phase III randomized clinical trial,^18^ atezolizumab plus bevacizumab improved PFS vs sunitinib in a subset of patients with PD-L1–positive metastatic kidney cell carcinoma (HR, 0.74; *P* = .022), and lastly, a phase Ib trial^19^ of pembrolizumab + lenvatinib for head and neck squamous cell carcinoma found an ORR of 36.4%. Atezolizumab in combination with bevacizumab enhanced antigen T cell migration in metastatic kidney cell carcinoma.^22^ Lenvatinib plus pembrolizumab resulted in significantly longer PFS and OS than sunitinib in a phase III randomized clinical trial for patients with advanced kidney cell carcinoma without prior systemic therapy.^23^ Grade 3 and 4 toxic effect rates reported from these series,^18,19,20,21,22,23^ in general, were half of what was reported with PD1 inhibition in combination with chemotherapy,^16,17^ and better patient- reported quality of life have also been found.^20,24^

A systemic review^25^ of angiogenesis inhibitors for head and neck cancer, which included trials that involved bevacizumab, found high rates of toxic effects, but some trials had encouraging and favorable efficacy results. A phase II randomized clinical trial^26^ did not find improvements in PFS or OS with the addition of bevacizumab to carboplatin and paclitaxel for recurrent or metastatic NPC. However, authors noted that there was improvement in the tumor shrinkage rate and concluded that this combination may be an option for NPC with a substantial tumor load. These studies performed in the recurrent or metastatic setting helped provide insights and may pave the way for combination therapies in the curative setting after patients have received standard chemoradiation. Mixed results for the addition of bevacizumab to chemotherapy suggest the need for personalization.

Approximately 20% of patients developed distant metastasis for NPC despite aggressive chemoradiation, and the key may be to determine who these patients are.^5^ There is a need to individualize treatment and identify biomarkers associated with early response, outside of traditional T and N staging, that are associated with outcomes; traditional T and N staging has limitations in its ability to discriminate which patients may be likely to develop distant metastasis given the high rates toxic effects that patients with NPC experience when undergoing chemoradiation.^27,28^ A study^29^ found an association between the level of circulating Epstein-Barr virus (EBV) DNA and disease stage, tumor recurrence, and patient survival after chemoradiation. To date, postradiation plasma EBV DNA has been the most robust biomarker associated with disease outcomes and has been used to monitor recurrence after definitive therapy.^30^ Increased levels of posttreatment plasma EBV DNA have been found to predate clinical recurrence by 3 to 7 months.^31^ A 2021 study^32^ also found superior survival among patients with NPC and initial detectable plasma EBV DNA levels after chemoradiation who subsequently experienced clearance vs those who never cleared; the survival rate was comparable with that of patients who initially had undetectable postradiotherapy plasma EBV DNA levels. These findings suggest that patients with NPC and increased levels of plasma EBV DNA may be an enriched group of patients with NPC at the highest risk of recurrence; this may be a population to select for additional adjuvant treatment after definitive chemoradiation, such as VEGF inhibition or PD1 or PDL1 inhibition. This personalized approach may be associated with reductions in overtreatment of patients with undetectable plasma EBV DNA levels.

### Limitations

A limitation of this study was the small, nonrandomized sample size of 44 patients with locoregionally advanced NPC. Nonetheless, our findings suggest that VEGF inhibition may be combined safely with standard chemoradiation. We did not observe excessive rates of toxic effects with this combination even when tumors were close to major blood vessels, which is almost always the case for locoregionally advanced NPC. Another limitation was that the staging system for NPC has changed since the completion of this study. Given that our primary end point was safety and toxic effects, we do not believe that the change in the staging system would be associated with changes in our end point. Furthermore, 10 patients in the entire cohort may possibly be downstaged given the change in the staging system since the completion of this study, and therefore, we do not feel that excellent treatment outcomes were purely outcomes associated with staging migration from T4 (AJCC Staging Manual, 7th Edition) to T2 (AJCC Staging Manual, 8th Edition).^33^

## Conclusions

In this phase II nonrandomized controlled trial, no grade 4 hemorrhage or grade 5 AEs were reported with the addition of bevacizumab to CCRT for locally or regionally advanced NPC. The relatively low rate of distant metastasis despite nearly 90% of patients presenting with stage III to IVB disease is intriguing and may warrant further investigation. Given that 20% of patients died from disease,^12^ suggesting that considerations may be made to combine bevacizumab with other systemic therapy, such as PD1 or PDL1 inhibition, with further associated improvements in outcomes. Selecting which patients with NPC may be at the highest risk of recurrence using plasma EBV DNA may be key to further refining treatment so that added toxic effects may not outweigh potential benefits.

## Notes

Supplement 1.
Trial Protocol

Supplement 2.
Data Sharing Statement
