# Supplementary material for: Long-term Outcomes of Bevacizumab and Chemoradiation for Locoregionally Advanced Nasopharyngeal Carcinoma: A Nonrandomized Controlled Trial
Source: JAMA Netw Open. 2023 Jun 2;6(6):e2316094. doi: 10.1001/jamanetworkopen.2023.16094 (PMC10238946; doi:10.1001/jamanetworkopen.2023.16094)
Supplement: Supplement 1. — Trial Protocol [file jamanetwopen-e2316094-s001.pdf]

RADIATION THERAPY ONCOLOGY GROUP

RTOG 0615

A PHASE II STUDY OF CONCURRENT CHEMORADIOTHERAPY USING THREE-DIMENSIONAL CONFORMAL RADIOTHERAPY (3D-CRT) or INTENSITY-MODULATED RADIATION THERAPY (IMRT) + BEVACIZUMAB (BV) FOR LOCALLY OR REGIONALLY ADVANCED NASOPHARYNGEAL CANCER

NCI-supplied agent: Bevacizumab (NSC 704865; IND 79211)

Study Chairs

Principal Investigator/Radiation Oncology
Nancy Lee, MD
Memorial Sloan-Kettering Cancer Center
1275 York Avenue, Box #22
New York, NY 10021
212-639-3341/FAX 212-639-2417
Leen2@mskcc.org

Medical Oncology Co-Chair
David G. Pfister, MD
Memorial Sloan-Kettering Cancer Center
1275 York Avenue
New York, NY 10021
212-639-8235/FAX 212-717-3278
pfisterd@mskcc.org

Radiation Oncology Co-Chair, U.S.
Adam Garden, MD
MD Anderson Cancer Center
1515 Holcombe Blvd., Suite 97
Houston, TX 77030
713-792-3400/FAX 713-794-5573
agarden@mdanderson.org

Pathology/Biomarker Co-Chair
Kian A. Ang, MD
MD Anderson Cancer Center
1515 Holcombe Blvd., Suite 97
Houston, TX 77030
713-563-2300/FAX 713-563-2331
kianang@mdanderson.org

Radiation Oncology Co-Chair, Canada
John Kim, MD
Princess Margaret Hospital
610 University Avenue
Toronto, Canada M5G 2M9
416-946-2126/FAX 416-946-6561
John.Kim@rmp.uhn.on.ca

Pathology/Biomarker Co-Chair
Anthony T.C. Chan, MD
Chinese University of Hong Kong
Prince of Wales Hospital, Shatin
New Territories, Hong Kong
852-2632-2119/FAX 852-2649-7426
anthonytcchan@cuhk.edu.hk

Medical Physics Co-Chair
James Mechalakos, PhD
Memorial Sloan-Kettering Cancer Center
1275 York Avenue
New York, NY 10021
212/639-2904/FAX 212-717-3258
mechalaj@mskcc.org

Senior Statistician
Qiang Zhang, PhD
Radiation Therapy Oncology Group/ACR
1818 Market Street, Suite 1600
Philadelphia, PA 19103
215-574-3197/FAX 215-928-0153
ezhang@acr-arrs.org

Activation Date: December 13, 2006
Closure Date; February 5, 2009
Update Date: October 22, 2009
Version Date: February 16, 2011
Includes Amendments 1-4
(Broadcast 2/24/11)

RTOG Headquarters
1-800-227-5463, ext. 4189

This protocol was designed and developed by the Radiation Therapy Oncology Group (RTOG) of the American College of Radiology (ACR). It is intended to be used only in conjunction with institution-specific IRB approval for study entry. No other use or reproduction is authorized by RTOG nor does RTOG assume any responsibility for unauthorized use of this protocol.

INDEX

Schema

Eligibility Checklist

1.0 Introduction

2.0 Objectives

3.0 Patient Selection

4.0 Pretreatment Evaluations/Management

5.0 Registration Procedures

6.0 Radiation Therapy

7.0 Drug Therapy

8.0 Surgery

9.0 Other Therapy

10.0 Tissue/Specimen Submission

11.0 Patient Assessments

12.0 Data Collection

13.0 Statistical Considerations

References

Appendix I - Sample Consent Form
Appendix II - Study Parameters
Appendix III - Performance Status Scoring
Appendix IV - Staging System
Appendix V - Management of Dental Problems
Appendix VI - Specimen Plug Kit
Appendix VII - Blood Collection Kit Instructions

RADIATION THERAPY ONCOLOGY GROUP

RTOG 0615

A Phase II Study of Concurrent Chemoradiotherapy Using Three-Dimensional Conformal Radiotherapy (3D-CRT) or Intensity-Modulated Radiation Therapy (IMRT) + Bevacizumab (BV) [NSC 708865; IND 7921] for Locally or Regionally Advanced Nasopharyngeal Cancer

SCHEMA (4/21/08)

	**Concurrent Phase**	**Adjuvant Phase**
**R**	**3D-CRT or IMRT**[a]	**Chemotherapy**[b]
**E**	Gross disease PTV: 70 Gy/33 fractions	5-Fluorouracil:1000 mg/m^2/day, Days 64-67, 85-88, 106-109
**G**		
**I**	Subclinical region PTV: 59.4 Gy/33 fractions	Cisplatin: 80 mg/m^2/day, Days 64, 85, 106
**S**		
**T**	**Chemotherapy**[b]	**Biologic Therapy**
**E**	Cisplatin: 100 mg/m^2/day, Days 1, 22, and 43	Bevacizumab: 15 mg/kg/day, Days 64, 85, 106
**R**		
	**Biologic Therapy**	
	Bevacizumab: 15 mg/kg/day, Days 1, 22, and 43	

Note: The dosing date for cisplatin; cisplatin/5-fluorouracil/bevacizumab; and/or bevacizumab alone may be delayed for up to 3 days for logistical reasons (e.g., holiday, patient scheduling conflict).

a. See Section 5.1-5.3 for pre-registration requirements; NOTE: It is mandatory that the treating physician determine the radiation therapy technique (3D-CRT vs. IMRT) to be used prior to the site registering the patient. See Section 6.0 for details of radiation therapy.
b. See Section 7.0 for details of drug therapy.

Patient Population: (See Section 3.0 for Eligibility)
Confirmed histopathologic diagnosis of nasopharyngeal carcinoma: Types WHO I-IIb/III, Stage IIB-IVB; no head and neck surgery of the primary tumor or lymph nodes, except for incisional or excisional biopsies

Required Sample Size: 46

RTOG Institution # _________

RTOG 0615 **ELIGIBILITY CHECKLIST (6/12/08)**

Case # _________

________(Y) 1. Is the primary tumor site arising from the nasopharynx?

________(Y) 2. Does the patient have Stage IIB-IVB disease?

________(N) 3. Does the patient present with T1-2N1 disease in which node positivity is based on the presence of retropharyngeal lymph nodes?

________(Y) 4. Is the confirmed histology type WHO I-IIb/III?

________(N) 5. Is there evidence of distant metastases?

________(Y) 6. Were the required diagnostic tests completed as listed in Section 3.1.3?

________(Y) 7. Is the Zubrod 0-1?

________(Y) 8. Is the patient at least 18 years of age?

________(Y) 9. Were the following lab parameters confirmed within 2 weeks prior to registration?

- WBC ≥ 4,000/cmm:
- ANC ≥ 1,500 mm^3
- Platelets ≥ 100,000 cells/mm^3
- Hemoglobin ≥ 9.0 g/dl
- Serum creatinine ≤ 1.5 mg/dl or calculated creatinine clearance (CC) ≥ 55 ml/min
- International normalized ratio (INR) ≤1.5 X UNL
- Activated partial thromboplastin time (aPTT)≤ 1.5 X UNL
- Total bilirubin ≤ 1.5 X UNL
- Aspartate aminotransferase ≤ 1.5 X UNL
- Alanine aminotransferase ≤ 1.5 X UNL
- Alkaline phosphatase ≤ 1.5 X UNL

________(Y) 10. Was the urine protein: creatinine ratio (UPC ratio) < 1.0 within 8 weeks prior to registration?

_______(Y/NA) 11. For women of childbearing potential, was a serum pregnancy test completed within 2 weeks of registration?

________(Y) If yes, was the serum pregnancy test negative?

_______(Y/NA) 12. If a male participant or a woman of child bearing potential, did the patient agree to practice effective birth control throughout the treatment phase of the study (until at least 60 days following the last study treatment)?

________(Y) 13. Per the investigator, is the patient's nutritional and physical condition compatible with the proposed treatment regimen?

________(Y) 14. Did the patient sign a study specific informed consent prior to registration?

________(N) 15. Did the patient have a prior invasive malignancy (with the exception of non-melanomatous skin cancer)?

_______(Y) If yes, has the patient been disease free for at least three years?

(Continued on the next page)

________(N) 16. Did the patient have previous irradiation to the region of the study cancer that would result in overlap in radiation fields?

________(N) 17. Did the patient have previous chemotherapy for the study cancer?

________(N) 18. Did the patient have prior treatment with bevacizumab or other agents specifically targeting VEGF?

_______(N) 19. Was there surgery on the primary tumor or lymph nodes (with the exception of incisional or excisional biopsies?

________(N) 20. Did the patient have gross hemoptysis or hematemesis (defined as bright red blood of 1 teaspoon or more or frank clots within minimal or no phlegm) within 4 weeks prior to registration?

________(N) 21. Is the patient receiving other experimental therapeutic cancer treatment?

________(N) 22. Was the patient's blood pressure at baseline > 150/100 mmHg?

________(N) 23. Does the patient have a CTCAE, v. 3.0 ≥ grade 2 hearing deficit confirmed on baseline audiogram, which in the judgment of the investigator is felt to have primarily a sensorineural basis?

________(N) 24. Does the patient have peripheral neuropathy CTCAE, v. 3.0 ≥ grade 2?

________(N) 25. Does the patient have any of the severe comorbid conditions listed in Section 3.2.14 that would exclude him/her from participation?

_______(Y/N) 26. Is the patient currently taking warfarin, heparin, daily treatment with aspirin (> 325 mg/day), dipyramidole (Persantine®), ticlopidine (Ticlid®), clopidogrel (Plavix®), or cilostazol (Pletal®), or nonsteroidal anti-inflammatory medications known to inhibit platelet function?

_______(Y) If yes, did the patient discontinue these medications ≥ 10 days prior to registration?

_______(N) 27. Has the patient had prior allergic reactions to the study drug(s) involved in this study?

<u>The following questions will be asked at Study Registration:</u>

IMRT CREDENTIALING IS REQUIRED BEFORE REGISTRATION.

____________(N/Y) Specify use of IMRT (Question 22)

____________ 1. Name of institutional person registering this case?

____________(Y) 2. Has the Eligibility Checklist (above) been completed?

(Continued on the next page)

RTOG Institution # ________

RTOG 0615 **<u>ELIGIBILITY CHECKLIST</u> (12/13/06)**

Case # ________ (page 3 of 3)

______(Y) 3. Is the patient eligible for this study?

______ 4. Date the study-specific Consent Form was signed? (must be prior to study entry)

______ 5. Patient's Initials (First Middle Last) [May 2003; If no middle initial, use hyphen]

______ 6. Verifying Physician

______ 7. Patient's ID Number

______ 8. Date of Birth

______ 9. Race

______ 10. Ethnic Category (Hispanic or Latino; Not Hispanic or Latino; Unknown)

______ 11. Gender

______ 12. Patient's Country of Residence

______ 13. Zip Code (U.S. Residents)

______ 14. Patient's Insurance Status

______ 15. Will any component of the patient's care be given at a military or VA facility?

______ 16. Calendar Base Date

______ 17. Registration/randomization date: This date will be populated automatically.

______ 18. Medical Oncologist's name

______(Y/N) 19. Tissue and blood kept for cancer research?

______(Y/N) 20. Tissue and blood kept for medical research?

______(Y/N) 21. Allow contact for future research?

The Eligibility Checklist must be completed in its entirety prior to web registration. The completed, signed, and dated checklist used at study entry must be retained in the patient's study file and will be evaluated during an institutional NCI/RTOG audit.

Completed by ______________________ Date ________________

1.0 INTRODUCTION

1.1 Current Treatment for Locally or Regionally Advanced Nasopharyngeal Carcinoma

Treatment with concurrent cis-platinum (CDDP) chemotherapy followed by adjuvant chemotherapy (CDDP and 5-fluorouracil [5FU]) is the current standard of care for patients with locally or regionally advanced nasopharyngeal carcinoma.[1] Recent meta-analyses continue to demonstrate an added benefit in terms of overall survival and event-free survival with the addition of chemotherapy to radiation therapy.[2-3]

Radiation therapy techniques used in these trials were largely conventional two-dimensional (2D) planning. Due to the high radiation doses that were delivered to the major parotid glands bilaterally, patients experienced permanent xerostomia. In general, salivary flows are markedly reduced when 10-15 Gy of radiation is delivered to most of the gland.[4-5] High doses to most of the parotid glands will result in irreversible and permanent xerostomia. The degree of xerostomia is largely dependent on the radiation dose and the volume of the salivary gland that is in the radiation field. As a result, patients' quality of life is compromised as they experience changes in speech and taste. The oral dryness also predisposes the patients to fissures, ulcers, dental caries, infection, and in worst cases, osteoradionecrosis.[6-9] Lastly, although the achieved local progression-free rates for T1 and T2 tumors ranged from 64-95% with conventional 2D radiotherapy, these rates dropped to 44-68% in more advanced T3/T4 lesions.[10-16]

Over the past decade, radiotherapy techniques for nasopharyngeal carcinoma have changed.[17] Due to limitations in terms of adequate tumor coverage by conventional 2D radiotherapy techniques, a shift to three-dimensional (3D) methods was made and has resulted in superior coverage of the tumor while not exceeding the tolerance of nearby normal tissues, such as the brain stem and optic structures.[17] Several centers have reported their results in using 3D conformal radiotherapy (3D-CRT) in the treatment of nasopharyngeal carcinoma.[17-20] The three-year loco-regional failure-free rate was as high as 77% for advanced T4 disease treated with 3D-CRT in one series.[19] Furthermore, since the introduction of intensity modulated radiation therapy (IMRT), a more refined and more advanced type of 3D-CRT, the modulation of the intensity of the radiation beams such that a high dose can be delivered to the tumor while significantly reducing the dose to the surrounding normal tissue can be seen.[21-24] Xia, et al. compared IMRT treatment plans with conventional treatment plans for a case of locally advanced nasopharyngeal carcinoma. They concluded that IMRT provided improved tumor target coverage with significantly more sparing of sensitive normal tissue structures (not only the brain stem and optic structures but also the parotid glands) in the treatment of locally advanced nasopharyngeal carcinoma.[25] Two recent papers also substantiated this finding. The authors stated that because there was a lack of a major benefit with conventional 3D planning used only during the boost phase of treatment for nasopharyngeal carcinoma, they are currently using IMRT to deliver the entire course of radiation at their institution.[26-27]

At the University of California-San Francisco Medical Center, IMRT has been used for the treatment of nasopharyngeal carcinoma. With a median follow-up of 31 months, the local progression-free rate is 97% and the regional progression-free rate is 98% with a four year overall survival rate of 88%.[28] Patients' salivary function also recovered over time. Other single institutions also substantiated these findings.[30-32] Lastly, one recent phase III randomized trial from Hong Kong, although only reported in abstract form, showed the benefit of IMRT in terms of salivary sparing.[29]

In summary, IMRT with concurrent CDDP chemotherapy followed by adjuvant chemotherapy consisting of CDDP and 5-FU achieved superb control [91-96%] of the disease in patients who presented with locoregionally advanced nasopharyngeal carcinoma.[19-20, 28-32] Patients treated with IMRT also had a chance for recovery of their salivary function. However, the development of distant metastases after these combined modality treatments was still problematic (22-43%) and ultimately resulted in patient death. Therefore, more effective systemic therapy is highly warranted to improve overall survival for these patients.

1.2 VEGF and Nasopharyngeal Carcinoma

Increased vascular endothelial growth factor-A (VEGF-A) expression has been associated with poor prognosis in squamous cell carcinoma of the head and neck. A meta-analysis of twelve studies evaluating VEGF-A expression in 1002 patients with head and neck squamous cell carcinoma showed that positive VEGF staining was associated with an almost two-fold higher

risk of death at 2 years.[33] VEGF has been shown to play an important role in lymph node metastasis through the induction of angiogenesis in nasopharyngeal carcinoma.[34] In another study, Qian, et al. has shown that the levels of serum VEGF were significantly elevated in 65 patients with metastatic nasopharyngeal carcinoma.[35] Lastly, over expression of VEGF was seen in 67% of nasopharyngeal cases and the higher expression of VEGF in Epstein Barr Virus (EBV) positive tumors was related to higher rate of recurrence, nodal positivity, and lower survival.[36] A recent pilot study by Druzgal, et al. analyzed the pre- and post-treatment serum levels of cytokines and angiogenesis factors as markers for outcome in patients with head and neck cancer.[37] With a median follow-up of 37 months, patients were more likely to remain disease free when the VEGF level decreased post-treatment versus those who continued to have increased VEGF levels after treatment. In this study, 7% of the patients had nasopharyngeal carcinoma.

1.3 Bevacizumab in Solid Tumors

VEGF is a secreted, heparin-binding protein that exists in multiple isoforms. Action of VEGF is primarily mediated through binding to the receptor tyrosine kinases, VEGFR-1 (Flt-1) and VEGFR-2 (KDR/Flk-1). The biological effects of VEGF include endothelial cell mitogenesis and migration, increased vascular permeability, induction of proteinases leading to remodeling of the extracellular matrix, and suppression of dendritic cell maturation. VEGF-A is the most potent and specific growth factor for endothelial cells.[38]

Bevacizumab (Avastin™) is a recombinant humanized IgG1 monoclonal antibody (MAb) that binds all active isoforms of human vascular endothelial growth factor (VEGF or VEGF-A) with high affinity (k_d = 1.1 nM).[39] Of known pro-angiogenic factors, VEGF is one of the most potent and specific factor and has been identified as a crucial regulator of both normal and pathological angiogenesis.

Pre-Clinical Studies

The murine parent MAb of bevacizumab, A4.6.1, has demonstrated potent growth inhibition *in vivo* in a variety of human cancer xenograft and metastasis models, including those for SK-LMS-1 leiomyosarcoma, G55 glioblastoma multiforme, A673 rhabdo myosarcoma, Calu-6, and MCF-7 cell lines.[39-42] The antitumor activity was enhanced with the combination of A4.6.1 and chemotherapeutic agents compared to either agent alone. Furthermore, combined blockage of the VEGF pathway and other growth factor pathways (e.g., EGFR or PDGFR) has also demonstrated additive effects *in vivo.*[43-44] Associated with the anti-tumor activity of anti-VEGF MAbs were findings of reduced intratumorial endothelial cells and microcapillary counts as well as reduced vascular permeability and interstitial pressure.

Pre-clinical toxicology studies have examined the effects of bevacizumab on female reproductive function, fetal development, and wound healing. Fertility may be impaired in cynomolgus monkeys administered bevacizumab, which led to reduced uterine weight and endometrial proliferation as well as a decrease in ovarian weight and number of corpora lutea. Bevacizumab was found to be teratogenic in rabbits, with increased frequency of fetal resorption, specific gross and skeletal fetal alterations. In juvenile cynomolgus monkeys with open growth plates, bevacizumab induced physeal dysplasia which was partially reversible upon cessation of therapy. Bevacizumab also delays the rate of wound healing in rabbits, and this effect appeared to be dose-dependent and characterized by a reduction of wound tensile strength.

Clinical Studies

To date, over 3000 patients have been treated in clinical trials with bevacizumab as monotherapy or in combination regimens.[45]

The pharmacokinetics (PK) of bevacizumab have been characterized in several phase I and phase II clinical trials, with doses ranging from 1 to 20 mg/kg administered weekly, every 2 weeks, or every 3 weeks. The estimated half-life of bevacizumab is approximately 21 days (range 11-50 days). The predicted time to reach steady state was 100 days. The volume of distribution is consistent with limited extravascular distribution.

The maximum tolerated dose (MTD) of bevacizumab has not been fully determined. The dose level of 20 mg/kg was associated with severe headaches.[46] The dose schedule of either 10 mg/kg q2w, or 15 mg/kg q3w has been used in most phase II or III trials with only a few

exceptions (e.g., the pivotal phase III trial in colorectal cancer, in which bevacizumab was given at 5 mg/kg q2w).

Clinical proof of principle for anti-VEGF therapy with bevacizumab has been provided by the pivotal phase III trial of bevacizumab (5 mg/kg q2w) in combination with bolus irinotecan/5-FU/leucovorin (IFL) in patients with untreated advanced colorectal cancer (CRC).[47] In that study, the addition of bevacizumab to IFL was associated with an increase in objective responses (45% vs. 35%) and significant prolongations of both time-to-progression (TTP) [10.6 vs. 6.2 months] and median survival (20.3 vs. 15.6 months) as compared to IFL. However, in the phase III trial in previously treated metastatic breast cancer, the addition of bevacizumab to capecitabine did not show a difference in TTP despite an increase in the response rate from 9% to 20%.[48]

Bevacizumab has also been studied in renal cell cancer (RCC). In a three-arm, double-blind, placebo-controlled phase II trial,[49] patients with previously treated stage IV RCC were randomized to high-dose (HD) bevacizumab (10 mg/kg q2w), low-dose (LD) bevacizumab (3 mg/kg q2w) or placebo. The study demonstrated a highly significant prolongation of TTP in the HD arm (4.8 months) as compared with the placebo (2.6 months) [hazard ratio = 2.55, p = 0.0002]; the LD arm was associated with a smaller difference in TTP (3.0 months) of borderline significance. The tumor response rate was 10% in the HD arm but 0% in the LD and placebo groups.

Bevacizumab inhibits tumor neovascularization. As stated above, bevacizumab also may improve the delivery of chemotherapy to tumors by normalizing the permeability of existing tumor vasculature via decreasing tumor interstitial fluid pressure.[50] As a monotherapy compared to placebo, bevacizumab offered superior progression-free survival as well as complete and partial response rates for metastatic renal cell carcinoma and recurrent ovarian cancer, respectively.[49, 51] Bevacizumab combined with chemotherapy has also shown an improved overall survival in several randomized trials when compared to standard chemotherapy, i.e. metastatic colorectal, lung, and breast cancer.[52-54] The incidence of bleeding was 31% in central located (see Section 1.3 below) squamous cell lung cancer patients who received chemotherapy and bevacizumab.[55] The FDA approved bevacizumab as a first-line therapy for patients presenting with metastatic colorectal cancer in combination with 5-FU based chemotherapy.

Bevacizumab also may enhance the effects of radiotherapy.[56] Preliminary data from Willet shows that of the 9 patients with locally advanced rectal cancer who underwent neoadjuvant chemoradiotherapy + bevacizumab (5 mg/kg), the first 6 patients completed treatment without dose-limiting toxicity.[57] Subsequently, these patients underwent surgery without perioperative complications. Six patients demonstrated limited microscopic disease.[58] As a continuation of the phase I trial, 5 additional patients received bevacizumab at 10mg/kg. Pathologic evaluation of the surgical specimens for staging after the completion of therapy showed 2 complete pathologic responses. This was not seen in patients who received 5mg/kg of bevacizumab. However, the complete responses were seen in the two patients who experienced intestinal dose-limiting toxicity.[57] The correlative investigations done supported the previous findings that bevacizumab has antivascular effects and normalizes the tumor vasculature. These issues and further validation of surrogate markers currently are being explored in the ongoing phase II trial at 5mg/kg, which was determined to be the maximum tolerated dose for rectal cancer.[57]

Additional clinical trials are ongoing in a variety of solid tumors and hematological malignancies using bevacizumab as monotherapy or in combination with chemotherapy, radiation, or other targeted/biological agents.

1.4 Bevacizumab Toxicities (5/7/07)

Although it is rare, there are potential life-threatening toxicities associated with bevacizumab, i.e., arterial thrombotic event (ATE) [1.7%], gastrointestinal (GI) perforation [2-4%], and hemorrhage, especially pulmonary hemorrhage (up to 31% in squamous non-small cell lung cancer). ATEs include deep vein thrombosis, myocardial infarction, cerebrovascular accident, angina, and transient ischemic attack. The risk of ATE increases to 3.8% when bevacizumab is added to chemotherapy.[55] Of 120 GI perforations, 113 occurred in patients with abdominal tumors versus 7 in non-abdominal cancers.[52] Hemorrhage is rare except in patients with centrally located tumors with squamous cell lung cancer.[55] In a subsequent ECOG phase III trial of chemotherapy ± bevacizumab in advanced NSCLC in which centrally located squamous non-small cell lung

cancer was excluded [n=842], the rate of ≥ grade 3 hemorrhage was 4.5% in the bevacizumab containing arm.[53] The ≥ grade 3 events included pulmonary hemorrhage (1.9%) CNS hemorrhage (1.0%), GI bleed (1.2%), and hemorrhage at other sites (1.0%). In this trial, bevacizumab also increased the rate of grade 4 neutropenia (24%), neutropenic fever, grade 4 thrombocytopenia (1.4%), and hypertension (6.0%).

Safety Profile
Based on clinical trials with bevacizumab as monotherapy or in combination with chemotherapy, the most common adverse events of any severity include asthenia, pain, headache, hypertension, diarrhea, stomatitis, constipation, epistaxis, dyspnea, dermatitis and proteinuria. The most common grade 3-4 adverse events were asthenia, pain, hypertension, diarrhea and leukopenia. The most serious AEs include life-threatening or fatal hemorrhage, arterial thromboembolic events, gastrointestinal perforation and wound dehiscence; these events were uncommon but occurred at an increased frequency compared to placebo or chemotherapy controls in randomized studies.

The following is a description of major adverse events associated with bevacizumab therapy. A list of Comprehensive Adverse Events and Potential Risks (CAEPR) in CTCAE, v. 3.0 terms is included in Section 7.6 of the protocol. Reference may also be made to the Investigators' Brochure and the FDA package insert (http://www.fda.gov/cder/foi/label/2004/125085lbl.pdf).

Infusion-Related Reactions: Infusion reactions with bevacizumab were uncommon (< 3%) and rarely severe (0.2%). Infusion reactions may include rash, urticaria, fever, rigors, hypertension, hypotension, wheezing, or hypoxia. Currently, there is no adequate information on the safety of retreatment with bevacizumab in patients who have experienced severe infusion-related reactions.

Hypertension: Hypertension is common in patients treated with bevacizumab, with a reported incidence of 20-30% in clinical trials. Initiation or increase of anti-hypertensive medications may be required, but in most cases, blood pressure (BP) can be controlled with routine oral drugs. However, incidents of hypertensive crisis with encephalopathy or cardiovascular sequelae have been rarely reported. BP should be closely monitored during bevacizumab therapy and the goal of BP control should be consistent with general medical practice. Bevacizumab therapy should be suspended in the event of uncontrolled hypertension.

Proteinuria: Proteinuria has been seen in all bevacizumab studies to date, ranging in severity from an asymptomatic increase in urine protein (incidence of about 20%) to rare instances of nephrotic syndrome (0.5% incidence). Pathologic findings on renal biopsies in two patients showed proliferative glomerulonephritis. NCI-CTCAE grade 3 proteinuria (> 3.5gm/24 hour urine) is uncommon, but the risk may be higher in patients with advanced RCC. In the randomized phase II study in RCC, 24-hour urine was collected in a subset of patients enrolled, and grade 3 proteinuria was found in 4 patients in the 10 mg/kg-arm (n=37), 2 patients in the 3mg/kg arm (n=35) and none in the placebo arm (n=38). The safety of continuing bevacizumab in patients with moderate or severe proteinuria has not been adequately tested.

Hemorrhage: The incidence of hemorrhagic events is increased with bevacizumab therapy. Epistaxis is common, occurring in 20-40% of patients, but it is generally mild and rarely requires medical intervention. Life-threatening and fatal hemorrhagic events have been observed in bevacizumab studies and included pulmonary hemorrhage, CNS bleeding and gastrointestinal (GI) bleeding. In a phase II study in non-small cell lung cancer, 6 cases of life-threatening hemoptysis or hematemesis were reported among 66 patients treated with bevacizumab and chemotherapy; 4 of these events were fatal.[59] In the pivotal phase III trial in advanced colorectal cancer, the rate of GI hemorrhage (all grades) was 24% in the IFL/bevacizumab arm compared to 6% in the IFL arm; grade 3-4 hemorrhage was 3.1% for IFL/bevacizumab and 2.5% for IFL. Serious GI hemorrhage has also been observed in clinical trials with bevacizumab in patients with pancreatic cancer or varices treated with bevacizumab.

Arterial Thromboembolic Events: The risk of arterial thromboembolic events is increased with bevacizumab therapy, and such events included cerebral infarction, transient ischemic attack (TIA), myocardial infarction and other peripheral or visceral arterial thrombosis. In the pivotal trial

in CRC (AVF2107), the incidence of arterial thromboembolic events was 1% in the IFL/placebo arm compared to 3% in the IFL/ bevacizumab arm. A pooled analysis of five randomized studies showed a two-fold increase in these events (4.4% vs. 1.9%). Certain baseline characteristics, such as age and prior arterial ischemic events, appear to confer additional risk.[60] In patients > 65 years treated with bevacizumab and chemotherapy, the rate of arterial thromboembolic events was approximately 8.5%.

Gastrointestinal Perforation/Fistula: GI perforations/fistula were rare but occurred at an increased rate in bevacizumab-containing therapies. The majority of such events required surgical intervention and some were associated with a fatal outcome. In the pivotal phase III trial in CRC (AVF2107), the incidence of bowel perforation was 2% in patients receiving IFL/bevacizumab and 4% in patients receiving 5-FU/bevacizumab compared to 0.3% in patients receiving IFL alone. GI perforation has also been reported in patients with gastric/esophageal cancer, pancreatic cancer, ovarian cancer or comorbid GI conditions such as diverticulitis and gastric ulcer. GI perforation should be included in the differential diagnosis of patients on bevacizumab therapy presenting with abdominal pain or rectal/abdominal abscess.

Wound Healing Complications: Bevacizumab delays wound healing in rabbits, and it may also compromise or delay wound healing in patients. Bowel anastomotic dehiscence and skin wound dehiscence have been reported in clinical trials with bevacizumab. The appropriate interval between surgery and initiation of bevacizumab required to avoid the risk of impaired wound healing has not been determined. However, all clinical trials with bevacizumab have required a minimum of 28 days from prior major surgery; experience in the pivotal trial in advanced CRC suggests that initiation of bevacizumab 29-50 days following surgery should be associated with a very low incidence of wound dehiscence. The optimal interval between termination of bevacizumab and subsequent elective surgery has not been determined either. In the pivotal study in CRC, 40 patients on the IFL/bevacizumab arm and 25 patients on the ILF/placebo arm underwent major surgery while on study; among them, significant post-operative bleeding or wound healing complications occurred in 4 of the 40 patients from the IFL/bevacizumab arm and none of the 25 patients from the IFL alone arm. Decisions on the timing of elective surgery should take into consideration the half-life of bevacizumab (average 21 days, with a range of 11-50 days).

Congestive Heart Failure: The risk of left ventricular dysfunction may be increased in patients with prior or concurrent anthracycline treatment. In phase III controlled clinical trials in metastatic breast cancer (AVF 2119g) in which all patients had received prior anthracyclines, congestive heart failure (CHF) or cardiomyopathy were reported in 7 patients (3%) in the bevacizumab/capecitabine arm compared to 2 (1%) in the capecitabine-only arm. No increase in CHF was observed in CRC trials with bevacizumab in combination with IFL or 5-FU.

Venous Thrombosis: Venous thromboembolic events reported in bevacizumab trials included lower extremity deep vein thrombosis (DVT), pulmonary embolism and rarely, mesenteric or portal vein thrombosis. In the pivotal phase 3 trial of IFL + bevacizumab (given at 5 mg/kg q2w), the overall incidences of G3-4 venous thromboembolic events were comparable in the two arms (15.1 vs. 13.6%).

Reversible Posterior Leukoencephalopathy Syndrome (RPLS) or similar clinical syndromes related to vasogenic edema of the white matter have been rarely reported in association with bevacizumab therapy (< 1%). Clinical presentations are variable and may include altered mental status, seizure and cortical visual deficit. Hypertension is a common risk factor and was present in most (though not all) patients on bevacizumab who developed RPLS. MRI scans are key to diagnosis and typically demonstrate vasogenic edema (hyperintensity in T2 and FLAIR images and hypointensity in T1 images) predominantly in the white matter of the posterior parietal and occipital lobes; less frequently, the anterior distributions and the gray matter may also be involved. RPLS should be in the differential diagnosis in patients presenting with unexplained mental status change, visual disturbance, seizure or other CNS findings. RPLS is potentially reversible, but timely correction of the underlying causes, including control of BP and interruption of the drug, is important in order to prevent progression to irreversible tissue damage.

Fertility and Pregnancy: Clinical data are lacking regarding the immediate or long-term effect of bevacizumab on fertility and pregnancy. However, bevacizumab is known to be teratogenic and detrimental to fetal development in animal models. In addition, bevacizumab may alter corpus luteum development and endometrial proliferation, thereby having a negative effect on fertility. As an IgG1, it may also be secreted in human milk. Therefore, fertile men and women on bevacizumab studies must use adequate contraceptive measures and women should avoid breast feeding. The duration of such precautions after discontinuation of bevacizumab should take into consideration the half-life of the agent (average 21 days, with a range of 11 to 50 days).

Immunogenicity: As a therapeutic protein, there is a potential for immunogenicity with bevacizumab. With the currently available assay with limited sensitivity, high titer human anti-bevacizumab antibodies have not been detected in approximately 500 patients treated with bevacizumab.

Neutropenia: When combined with chemotherapy, bevacizumab increased the risk of neutropenia compared to chemotherapy alone. In a phase III trial with IFL +/- bevacizumab in colorectal cancer, grade 3-4 neutropenia was 21% in the bevacizumab + IFL arm vs. 14% in the IFL arm (grade 4 neutropenia was 3% vs. 2%). In a phase III trial with carboplatin and paclitaxel +/- bevacizumab in NSCLC, the bevacizumab-containing arm was associated with an increased rate of grade 4 neutropenia (27% vs. 17%), febrile neutropenia (5.4% vs. 1.8%), and an increased rate of infection with neutropenia (4.4% vs. 2.0%) with three fatal cases in the bevacizumab + chemotherapy arm vs. none in the chemotherapy control arm.

Tracheoesophageal (TE) Fistula: In a phase II trial of concurrent chemoradiation and bevacizumab in limited SCLC (concurrent irinotecan, carboplatin, radiotherapy, and bevacizumab, followed by maintenance bevacizumab), among the first 25 patients enrolled, there have been two confirmed cases of tracheoesophageal (TE) fistula (one fatal) and a third case of fatal upper aerodigestive tract hemorrhage, with TE fistula suspected but not confirmed. All three events occurred during the bevacizumab maintenance phase (1.5 to 4 months after completion of concurrent bevacizumab and chemoradiation). The TE fistula rate in this trial was higher than expected with chemoradiation alone. While pulmonary fistula (including TE fistula) also has been observed in NSCLC or SCLC patients receiving bevacizumab and chemotherapy (without radiation), the incidence was extremely low (≤ 1%), and the relationships to treatment vs. tumor in those cases was unclear. Experience is limited for bevacizumab administered sequentially after chemoradiation for either NSCLC or SCLC; in a study of chemoradiation followed by bevacizumab in SCLC, one of the 60 patients enrolled developed TE fistula.

1.5 Bevacizumab, Chemotherapy, and Radiation Therapy in Head and Neck Cancer

A recently completed phase I/II trial demonstrated the feasibility of bevacizumab (up to 15 mg/kg IV q3 weeks) + erlotinib (150 mg p.o QD) in patients with recurrent/metastatic head neck squamous cell carcinoma.[61] Bleeding occurred in 3 out of 58 patients (5.2%) in this study, in patients who underwent rebiopsies on day one of bevacizumab administration. Other grade ≥ 3 adverse events in this trial were rash, diarrhea, fatigue, and infection. The response rate was 14.6%. Another phase I trial of bevacizumab + 5-fluorouracil + hydroxyurea + RT (FHX) q2 weeks, for locally advanced HNSCC has been completed at the University of Chicago.[62] The following dose-limiting toxicities were seen at 10mg/m^2: two patients had grade 3 transaminase elevations and one patient experienced grade 4 neutropenia.[63] The authors concluded in their phase I trial that bevacizumab can be integrated with FXH chemoradiotherapy regimen at a dose of 10mg/m^2 every 2 weeks. There were no major additive toxicities observed. In the ongoing phase II study of this regimen, the bevacizumab dose is 10 mg/kg IV q2 weeks. Both of these studies demonstrated that bevacizumab administration with chemotherapy or chemoradiation is feasible in patients with HNSCC.

1.6 Bevacizumab Dose and Schedule with Chemotherapy and Radiation Therapy (5/7/07)

Pharmacokinetic studies have shown that the frequency of bevacizumab administration can be coordinated with the chemotherapeutic schedules.[64] For chemotherapy regimens administered q2 weeks, a recommended bevacizumab dose is 10 mg/kg on day 1 of each cycle.[65] In phase II/III trials of chemotherapy regimens administered q3 weeks, the bevacizumab dose was 15 mg/kg on day 1 of each cycle.[48,53,61] No data has shown an increased toxicity with the administration of bevacizumab 15 mg/kg q21 days. Pulmonary hemorrhage occurred in patients who received lower dose bevacizumab in a phase II randomized trail for advanced non-small cell

lung cancer.[55] The results of the pharmacokinetic model described above suggest that the total bevacizumab exposure would be similar for both bevacizumab regimens.

Preclinical data has shown that tumors cannot grow beyond 3 mm in diameter without developing a vascular supply[66] and there is evidence of host vasculature recruitment when tumor implants reaches100-300 cells.[67] This preclinical evidence supports the theory that in patients who are cured of their disease initially, with upregulation of angiogenesis, a progression from minimal residual disease to macroscopic recurrence can occur.[68-69] Administration of bevacizumab theoretically minimize the growth of these micrometastases.

1.7 Rationale for the Addition of Bevacizumab to Chemotherapy and IMRT (5/7/07)

Given that a) the predominant pattern of failure in locoregionally advanced nasopharyngeal cancer treated with IMRT and concurrent chemotherapy followed by adjuvant chemotherapy is distant metastasis and that b) nasopharyngeal cancer patients with elevated VEGF have a higher likelihood of recurrence, distant metastases, and decreased survival, it is logical to test the addition of bevacizumab (as a monoclonal antibody directed against VEGF) to the present treatment strategy for this group of patients. The rationale is that if bevacizumab, by targeting against VEGF, can potentially further reduce the rate of distant metastases and improve disease-free survival without significant toxicity associated with treatment, then the overall survival of nasopharyngeal cancer patients can be further improved. Bevacizumab will be administered during each phase of treatment. During the concurrent and adjuvant phases, the addition of bevacizumab is to potentiate the chemotherapeutic effects on distant micrometastasis.

1.8 Molecular Biomarker Studies (5/7/07)

Pre-treatment EBV DNA in serum or plasma has been proven to correlate with cancer stage,[70] clinical outcome,[71] and prognosis in patients with endemic nasopharyngeal cancer.[72] Post-treatment EBV DNA has an even better correlation with prognosis and has been used to monitor recurrence during post-treatment surveillance.[73-76] In 17 patients that were followed longitudinally, tumors recurred in 6 patients, and these patients had significant elevations in serum EBV DNA, even up to 6 months prior to tumor recurrence.[72] Low or undetectable levels of serum EBV DNA were observed in patients who remained in remission. In another study of early-staged nasopharyngeal carcinoma, with a median follow-up of 45 months, patients with distant failure had significantly higher pretreatment EBV DNA levels versus those without distant failure. The probability of distant metastasis was significantly higher in patients with high EBV DNA levels versus those who had low levels.[77] In a recently published phase II prospective study on 31 loco-regionally advanced nasopharyngeal carcinoma patients who underwent neoadjuvant chemotherapy followed by concurrent chemoradiation, serum EBV DNA was obtained at baseline, weekly during treatment, 4-6 weeks post-treatment, and q2 months for one year after treatment.[78] Serum EBV DNA level was found to be increased significantly in 8 of 9 patients who experienced treatment failure. Patients who had no evidence of disease did not experience elevated serum EBV DNA levels. Elevated EBV DNA has been shown to predate clinical recurrence by 3 to 7 months.[78-81] Detectable or high-level of post-treatment EBV DNA in plasma can predict a poor progression-free or overall survival when compared with undetectable or low DNA level[73,75] and may be a marker of subclinical residual disease.

Given the above results, for this current study, we will obtain research blood samples at baseline, within 2 weeks after the completion of adjuvant chemotherapy and bevacizumab and at 1 year after treatment from all patients who consent to participate in tissue/blood submission (see Section 10.0 for details of collection and submission). Preliminary analysis of the distribution of VEGF and VEGFR-2 expression (see Section 1.2) and serum EBV DNA titer will be performed by Dr. Anthony Chan and colleagues.

2.0 OBJECTIVES (5/7/07)

2.1 Primary Objective

2.1.1 To evaluate the safety and tolerability of bevacizumab (BV) plus chemoradiation

2.2 Secondary Objective

2.2.1 To determine the one- and two-year rates of local-regional progression-free interval, distant metastases-free interval, progression-free survival, and overall survival for stage IIB-IVB nasopharyngeal cancer, WHO types I-IIb/III treated with 3D-CRT or IMRT concurrent with cisplatin (CDDP) and BV, followed by adjuvant CDDP plus 5-fluorouracil plus BV

3.0 PATIENT SELECTION

NOTE: PER NCI GUIDELINES, EXCEPTIONS TO ELIGIBILITY ARE NOT PERMITTED.

3.1 Conditions for Patient Eligibility (11/7/07)

3.1.1 Biopsy proven (from primary lesion and/or lymph nodes) diagnosis of Stage IIB-IVB (AJCC, 6th ed.) non-metastatic cancer of the nasopharynx; Patients who present with T1-2N1 disease in which node positivity is based on the presence of retropharyngeal lymph nodes are not eligible.)

3.1.2 Histologic types WHO I-IIb/III;

3.1.3 Appropriate stage for protocol entry, including no distant metastases, based upon the following minimum diagnostic workup:

3.1.3.1 History/physical examination (must include a complete list of current medications, an assessment of weight and weight loss in the past 6 months, and an examination by a Medical Oncologist) within 4 weeks prior to registration;

3.1.3.2 Chest X-ray, PA and lateral (or other chest imaging, such as CT or PET/CT [with CT of diagnostic quality]) within 4 weeks prior to registration;

3.1.3.3 Pre-treatment evaluation of tumor extent with magnetic resonance imaging (MRI) with T1 contrast and T2 sequences within 6 weeks prior to registration; if MRI is medically contraindicated, obtain CT scan with ≤ 3 mm contiguous slices with contrast and bone windows (to evaluate base of skull involvement).

3.1.3.4 Liver CT (or other liver imaging, such as ultrasound or PET) within 4 weeks prior to registration: **Only** required at the discretion of the treating Medical Oncologist in the presence of elevated alkaline phosphatase, AST, or bilirubin or other clinical indicators highly suspicious for metastatic disease that is not detected on other imaging modalities;

3.1.3.5 Bone scan (or other bone imaging, such as bone survey or PET) within 4 weeks prior to registration: **Only** required at the discretion of the treating Medical Oncologist in the presence of elevated alkaline phosphatase or other clinical indicators highly suspicious for metastatic disease that is not detected on other imaging modalities;

3.1.4 Zubrod Performance Status 0-1;

3.1.5 Age ≥ 18;

3.1.6 CBC with differential obtained within 2 weeks prior to registration, with adequate bone marrow function defined as follows:

3.1.6.1 WBC ≥ 4,000/cmm;

3.1.6.2 Absolute neutrophil count (ANC) ≥ 1,500/ mm^3

3.1.6.3 Platelets ≥ 100,000 cells/ mm^3

3.1.6.4 Hemoglobin ≥ 9.0 g/dl.

3.1.7 Adequate renal function defined as serum creatinine ≤ 1.5 mg/dl or calculated creatinine clearance ≥ 55 ml/min with the following formula within 2 weeks prior to registration:

$$\text{Estimated Creatinine Clearance} = \frac{(140\text{-age}) \times WT(kg)}{72 \times \text{creatinine (mg/dl)}} \times 0.85 \text{ if female}$$

Note: A creatinine clearance based on a 24-hour urine collection also is permitted.

3.1.8 **(4/21/08)** Urine protein: creatinine ratio (UPC ratio) of < 1.0 within 8 weeks prior to registration; urine protein should be initially screened by urine analysis for UPC ratio. For UPC ratio ≤ 0.5, no further testing is required. For UPC ratio > 0.5, if dipstick is unavailable or if dipstick results are felt to be equivocal, then 24-hour urine protein should be obtained and the level should be < 1000 mg for patient enrollment.

Note: UPC ratio of spot urine is an estimation of the 24-urine protein excretion. A UPC ratio of 1 is roughly equivalent to a 24-hour urine protein of 1 gm. UPC ratio is calculated using one of the following formulae:

- [urine protein]/[urine creatinine] – if both protein and creatinine are reported in mg/dL
- [(urine protein) x 0.088]/[urine creatinine] – if urine creatinine is reported in mmol/L

3.1.9 Adequate hepatic function, defined as follows within 2 weeks prior to registration:

- Total bilirubin ≤ 1.5 X UNL;
- Aspartate aminotransferase ≤ 1.5 X UNL;
- Alanine aminotransferase ≤ 1.5 X UNL;
- Alkaline phosphatase ≤ 1.5 X UNL.

3.1.10 **(4/21/08)** International normalized ratio (INR) ≤ 1.5 and activated partial thromboplastin time (aPTT) ≤ 1.5 X UNL within 2 weeks prior to registration;

3.1.11 Per the investigator's assessment, the patient must have the nutritional and physical condition considered to be compatible with the proposed treatment regimen;

3.1.12 Serum pregnancy test within 2 weeks prior to registration for women of childbearing potential;

3.1.13 Women of childbearing potential and male participants must agree to use a medically effective means of birth control throughout their participation in the treatment phase of the study (until at least 60 days following the last study treatment);

3.1.14 Patients must sign study specific informed consent prior to registration.

3.2 Conditions for Patient Ineligibility (11/7/07)

3.2.1 Patients who present with T1-2N1 disease in which node positivity is based on the presence of retropharyngeal lymph nodes;

3.2.2 Prior invasive malignancy (except non-melanomatous skin cancer) unless disease-free for a minimum of 3 years (e.g., carcinoma in situ of the breast, oral cavity, or cervix are all permissible);

3.2.3 Prior systemic chemotherapy for the study cancer; note that prior chemotherapy for a different cancer is permitted;

3.2.4 Prior radiotherapy to the region of the study cancer that would result in overlap of radiation therapy fields;

3.2.5 Prior treatment with bevacizumab or other agents specifically targeting VEGF;

3.2.6 Head and neck surgery of the primary tumor or lymph nodes prior to registration, with the exception of incisional or excisional biopsies; **Note**: See Section 4.1.1 regarding timeframe restrictions for biopsies.

3.2.7 Patients with gross hemoptysis or hematemesis (defined as bright red blood of 1 teaspoon or more or frank clots within minimal or no phlegm per coughing episode) within 4 weeks prior to registration; patients with incidental blood mixed with phlegm are not excluded.

3.2.8 Patients receiving other experimental therapeutic cancer treatment;

3.2.9 Blood pressure at baseline > 150/100 mmHg;

3.2.10 Patients with hearing loss felt to be primarily sensorineural in nature, requiring a hearing aid or intervention (i.e., interfering in a clinically significant way with activities of daily living); conductive hearing loss from tumor-related otitis media is allowed.

3.2.11 Peripheral neuropathy CTCAE, v. 3.0 ≥ grade 2;

3.2.12 Severe, active co-morbidity, defined as follows:

3.2.12.1 Major medical or psychiatric illness, which in the investigators' opinion would interfere with the completion of therapy and follow up or with full understanding of the risks and potential complications of the therapy;

3.2.12.2 Unstable angina and/or congestive heart failure or peripheral vascular disease requiring hospitalization within the last 12 months, or other cardiac compromise that in the judgment of the investigator will preclude the safe administration of a study drug;

3.2.12.3 History of arterial thromboembolic events, transient ischemic attack (TIA), cerebral vascular accident (CVA), or transmural myocardial infarction (MI) ;

3.2.12.4 History of ongoing bleeding diathesis, hemorrhagic disorder, or coagulopathy within the last 6 months;

3.2.12.5 Chronic Obstructive Pulmonary Disease exacerbation or other respiratory illness requiring hospitalization or precluding study therapy <u>within 30 days prior to registration</u>;

3.2.12.6 History of abdominal fistula, gastrointestinal perforation, or intra-abdominal abscess within the last 6 months prior to registration;

3.2.12.7 Esophageal varices, non-healing ulcer, non-healing wound, or bone fracture within the last 6 months prior to registration;

3.2.12.8 Active, untreated infection and/or acute bacterial or fungal infection requiring intravenous antibiotics at the time of registration;

3.2.12.9 Hepatic insufficiency resulting in clinical jaundice and/or coagulation defects;

3.2.12.10 History of significant weight loss (> 15% from baseline);

3.2.12.11 Acquired Immune Deficiency Syndrome (AIDS) based upon current CDC definition; note, however, that HIV testing is not required for entry into this protocol. The need to exclude patients with AIDS from this protocol is necessary because the treatments involved in this protocol may be significantly immunosuppressive. Protocol-specific requirements also may exclude immuno-compromised patients.

3.2.13 Patients currently taking warfarin, heparin, daily treatment with aspirin (> 325 mg/day), or nonsteroidal anti-inflammatory medications known to inhibit platelet function; treatment with dipyramidole (Persantine®), ticlopidine (Ticlid®), clopidogrel (Plavix®), or cilostazol (Pletal®); **Note**: To be eligible for the study, patients must discontinue these medications ≥ 10 days prior

to study entry and must have an INR and an aPTT ≤ 1.5 X UNL within 2 weeks prior to registration (see Section 3.1.10);

3.2.14 Pregnancy or women of childbearing potential and men who are sexually active and not willing/able to use medically acceptable forms of contraception; this exclusion is necessary because the treatment involved in this study may be significantly teratogenic.

3.2.15 Prior allergic reaction to the study drug(s) involved in this protocol.

4.0 PRETREATMENT EVALUATIONS/MANAGEMENT

Note: This section lists baseline evaluations needed before the initiation of protocol treatment that do not affect eligibility.

4.1 Required Evaluations/Management

4.1.1 Incisional or excisional biopsies must NOT be done within 15 days prior to treatment.

4.1.2 Major surgical procedures, which in the treating physician's opinion would preclude the patient from participating in the trial, must NOT take place within 4 weeks prior to treatment.

4.1.3 Fine needle aspirations or placement of percutaneous gastrostomy tube (PEG) must NOT be done within 1 week prior to treatment.

4.1.4 Failure to perform one or more of the tests below may result in assessment of a protocol violation; see Appendix II.

- Electrocardiogram within 4 weeks prior to registration;
- Dental evaluation within 12 weeks prior to treatment;
- Baseline audiogram within 12 weeks prior to registration;

4.2 Highly Recommended Evaluations/Management

The following pre-treatment evaluations/interventions are not required but are highly recommended:

- The use of a PET/CT scan within 4 weeks prior to registration for treatment planning is optional. A PET/CT scan should not be substituted for the required pretreatment and follow-up MRIs of the nasopharynx and neck; however, PET or PET/CT scan results will satisfy, as indicated in Section 3.1, for chest, liver, and bone imaging requirements.
- A CT scan can be used for treatment planning when an MRI is medically contraindicated, but the scan must be within 6 weeks of start of 3D-CRT or IMRT. Treatment planning CT scans are not equivalent to diagnostic CT scans, even with contrast. Therefore, if an MRI is medically contraindicated, a diagnostic CT scan of the nasopharynx and neck should be done and will help to draw volumes on the treatment planning CT (see Section 3.1.3.3).
- Nutritional evaluation for a prophylactic gastrostomy (PEG) tube or gastrostomy-jejunostomy (G-J) tube placement is STRONGLY ENCOURAGED. Prophylactic placement must occur ≥ 1 week prior to treatment. For patients who do not have a PEG tube placed prophylactically and who subsequently require placement during treatment, bevacizumab must be stopped at least 1 week prior and for 1 week after the placement of the PEG tube.

5.0 REGISTRATION PROCEDURES

NOTE: It is mandatory the treating physician determine the radiation therapy technique (3D-CRT vs. IMRT) to be used prior to the site registering the patient.

5.1 Pre-Registration Requirements for 3D-CRT Treatment Approach

5.1.1 Only institutions that have met the technology requirements and that have provided the baseline physics information that are described in 3D-CRT Quality Assurance Guidelines may enter patients to this study.

5.1.2 The institution or investigator must complete a 3D questionnaire and/or set up an FTP account for digital data submission, both of which are available on the Image-Guided Center (ITC) web site at http://atc.wustl.edu prior to entering any cases. Upon review and successful completion of the "Dry-Run" or "Benchmark" QA test, the ITC will notify both the registering institution and RTOG Headquarters that the institution is eligible to enter patients onto this study. Institutions that have previously enrolled patients on 3D CRT trials of this same disease site may enroll patients on this study without further credentialing by the ITC.

5.2 Pre-Registration Requirements for IMRT Treatment Approach

5.2.1 In order to utilize IMRT on this study, the institution must have met specific technology requirements and have provided baseline physics information. Instructions for completing these

requirements or determining if they already have been met are available on the Radiological Physics Center (RPC) web site.

An IMRT phantom study with the RPC must be successfully completed (if the institution has not previously met this credentialing requirement on another RTOG IMRT Head and Neck study). Instructions for requesting and irradiating the phantom are available on the RPC web site at http://rpc.mdanderson.org/rpc/; select “Credentialing” and “RTOG”.

5.2.2 The institution or investigator must complete a new IMRT facility questionnaire and/or set up an FTP account for digital data submission, both of which are available on the Image-Guided Center (ITC) web site at http://atc.wustl.edu. Upon review and successful completion of the “Dry-Run” or “Benchmark” QA test, the ITC will notify both the registering institution and RTOG Headquarters that the institution is eligible to enter patients onto this study.

5.3 Regulatory Pre-Registration Requirements **(4/21/08)**

5.3.1 **U.S. sites and Canadian sites** must fax copies of the documentation below to the CTSU Regulatory Office (215-569-0206), along with the completed CTSU-IRB/REB Certification Form, http://www.rtog.org/pdf_file2.html?pdf_document=CTSU-IRBCertifForm.pdf, prior to registration of the institution’s first case:

- IRB/REB approval letter;
- IRB/REB approved consent (English Version)
- IRB/REB assurance number

5.3.2 *Pre-Registration Requirements FOR CANADIAN INSTITUTIONS*

5.3.2.1 Prior to clinical trial commencement, Canadian institutions must complete and fax to the CTSU Regulatory Office (215-569-0206) Health Canada’s Therapeutic Products Directorates’ Clinical Trial Site Information Form, Qualified Investigator Undertaking Form, and Research Ethics Board Attestation Form.

5.3.2.2 Note: International sites must receive written approval of submitted LOI forms from RTOG Headquarters prior to submitting documents to their local ethics committee for approval. See http://www.rtog.org/pdf_forms.html?members/forms=Intl_LOI_Form_1-2007.pdf.

Approved international sites fax copies of the documentation below, along with the completed International REC Certification Form, http://www.rtog.org/pdf_forms.html?members/forms Certification.doc to RTOG Headquarters (215-574-0300) prior to registration of the institution’s first case:

- REC approval letter;
- Informed Consent (English Version);
- Federalwide Assurance (FWA) number.

5.3.3 *For shipment of bevacizumab*:

The Principal Investigator (or authorized designee) at each participating institution may request bevacizumab from NCI’s Pharmaceutical Management Branch (PMB). See Section 7.6 for details.

5.4 Registration

5.4.1 *Online Registration*

Patients can be registered only after eligibility criteria are met.

Each individual user must have an RTOG user name and password to register patients on the RTOG web site. To get a user name and password:

- The investigator and research staff must have completed Human Subjects Training and been issued a certificate (Training is available via http://cme.cancer.gov/clinicaltrials/learning/humanparticipant-protections.asp).
- A representative from the institution must complete the Password Authorization Form at http://www.rtog.org/members/webreg.html (bottom right corner of the screen), and fax it to 215-923-1737. RTOG Headquarters requires 3-4 days to process requests and issue user names/passwords to institutions.

An institution can register the patient by logging onto the RTOG web site (http://www.rtog.org), going to “Data Center Login" and selecting the link for new patient registrations. The system triggers a program to verify that all regulatory requirements (OHRP assurance, IRB approval) have been met by the institution. The registration screens begin by asking for the date on which the eligibility checklist was completed, the identification of the person who completed the

checklist, whether the patient was found to be eligible on the basis of the checklist, and the date the study-specific informed consent form was signed.

Once the system has verified that the patient is eligible and that the institution has met regulatory requirements, it assigns a patient-specific case number. The system then moves to a screen that confirms that the patient has been successfully enrolled. This screen can be printed so that the registering site will have a copy of the registration for the patient's record. Two e-mails are generated and sent to the registering site: the Confirmation of Eligibility and the patient-specific calendar. The system creates a case file in the study's database at the DMC (Data Management Center) and generates a data submission calendar listing all data forms, images, and reports and the dates on which they are due.

If the patient is ineligible or the institution has not met regulatory requirements, the system switches to a screen that includes a brief explanation for the failure to register the patient. This screen can be printed.

(10/22/09) Institutions can contact RTOG web support for assistance with web registration: websupport@acr-arrs.org

In the event that the RTOG web registration site is not accessible, participating sites can register a patient by calling RTOG Headquarters, at (215) 574-3191, Monday through Friday, 8:30 a.m. to 5:00 p.m. ET. The registrar will ask for the site's user name and password. This information is required to assure that mechanisms usually triggered by web registration (e.g., drug shipment, confirmation of registration, and patient-specific calendar) will occur.

6.0 RADIATION THERAPY

Note: Radiotherapy must be given with either three-dimensional conformal radiotherapy (3D-CRT) or Intensity Modulated RT (IMRT) techniques. See pre-registration requirements for 3D-CRT and IMRT in Sections 5.1-5.2.

Protocol treatment must begin within 2 weeks after registration.

6.1 Dose Specifications (4/21/08)

Prescription dose shall be according to the following (also see Section 6.4):

6.1.1 PTV_{70} (CTV_{70} + margin) will receive 70 Gy in 33 fractions at 2.12 Gy per fraction.

6.1.2 $PTV_{59.4}$ ($CTV_{59.4}$ + margin) will receive 59.4 Gy in 33 fractions at 1.8 Gy per fraction. At the discretion of the treating physician, the dose to the high-risk subclinical region will be 59.4 Gy.

If the treating physician would like to treat all sites: primary, upper and lower neck with a single 3D-CRT or IMRT plan, the low neck will receive 54 Gy at 1.64 Gy per fraction. This is known as PTV_{54} (CTV_{54} + margin). However, if there are gross nodes in the low neck, the surrounding subclinical region will receive 59.4 Gy in this case, as it is still considered high risk due to the presence of neighboring gross nodes. In addition, at the discretion of the treating physician, small volume lymph nodes can receive 63 Gy in 33 fractions at 1.9 Gy per fraction instead of 70 Gy in 33 fractions at 2.12 Gy per fraction (see Section 6.1.4).

6.1.3 Alternatively, the low neck or supraclavicular field may be treated with conventional AP or AP/PA fields and will receive 25 fractions of 2.0 Gy/fraction, for a total of 50 Gy unless there are gross nodes in which all the gross nodes should receive the same dose as the PTV_{70}, except for small volume lymph nodes which can receive 63 Gy (see Section 6.1.4). The maximum point dose to the brachial plexus should not exceed 66 Gy.

6.1.4 The treating Radiation Oncologist has the option of prescribing an intermediate dose of 63 Gy, PTV_{63}, to small volume lymph nodes in close proximity to critical structures. The most common example of the appropriate application of this intermediate dose is the clinical scenario in which there are small lymph nodes in the lower neck close to the brachial plexus.

6.1.5 If a single 3D-CRT or IMRT approach is used, it is necessary to submit the DVH for this plan (as defined in Section 6.4, PTV_{54}). If a conventional technique (AP or AP/PA field) is used for the low neck and supraclavicular field, it will be treated with 25 fractions of 2.0 Gy/fraction, to a total dose of 50 Gy with a depth of 3 cm from the anterior surface for an AP field or midline for AP/PA fields. There will be no DVH for the conventional fields.

Treatment will be delivered once daily, 5 fractions per week, over 6 weeks and 3 days. All targets will be treated simultaneously. Treatment breaks must be clearly indicated in the treatment record along with the reason(s) for the treatment break(s). Treatment breaks, if necessary, should ideally not exceed five treatment days at a time and ten treatment days total. Treatment breaks should be allowed only for resolution of severe acute toxicity and/or for intercurrent illness and not for social or logistical reasons. Any treatment break(s) exceeding two treatment days for reasons other than toxicity/illness will be considered a protocol deviation.

6.1.6 The reported doses for PTV_{70}, PTV_{63}, $PTV_{59.4}$, and PTV_{54} shall include the prescription dose (Sections 6.1.1-6.1.2) as well as the maximum point dose for that PTV, % PTV receiving ≥ 110% and ≥ 115% for that PTV and the PTV receiving ≤ 93% of the prescribed dose for that

PTV, and the mean dose for that PTV. At the discretion of the treating physician, PTV_{63} is also allowed for small volume nodal disease.

All plans shall be normalized such that at least 95% of the volume of PTV_{70} is covered by the 70 Gy isodose surface. To avoid a minor variation:

- No less than 20% of PTV_{70} will receive ≥ 77 Gy and no more than 5% of PTV_{70} will receive ≥ 80.5 Gy.
- No more than 1% of any distinct PTV should receive ≤ 93% of the prescribed dose.

6.2 Technical Factors

6.2.1 *External Beam Equipment and Beam Delivery Methods*

Megavoltage equipment capable of delivering 3D conformal radiotherapy techniques or static intensity modulation with a multileaf collimator or dynamic intensity modulation (using a multileaf collimator or tomotherapy) is required. Other techniques are acceptable as long as dose specifications and constraints are satisfied.

Conventional anterior low-neck field(s) is/are allowed. The junction between a 3D-CRT or an IMRT dose distribution and a conventional dose distribution is dependent upon the IMRT technique used and on institutional philosophy. Institutions are required to protect the spinal cord at all times. Dosimetric details regarding the match between this field and the upper neck therapy should be provided.

6.3 Treatment Planning, Imaging and Localization Requirements

6.3.1 The immobilization device should at least include the head and neck. It is strongly encouraged that the participating centers also include the shoulders in the immobilization. This is to further ensure accurate patient set-up on a daily basis. A description of the immobilization system used by each institution and data regarding the range of positioning errors (if data exists) should be provided.

6.3.2 Treatment planning CT scans will be required to define gross target volume(s), and clinical target volume(s). MRI scans (required unless medically contraindicated) aid in delineation of the treatment volume on planning CT scans. Special attention should be paid to the skull base. The treatment planning CT scan should be acquired with the patient in the same position and using the same immobilization device as for treatment.

All tissues to be irradiated must be included in the CT scan. CT scan thickness should be ≤ 0.3 cm slices through the region that contains the primary target volumes. The regions above and below the target volume may be scanned with 0.5 cm slice thickness. MRI scans assist in definition of target volumes, especially when targets extend near the base of skull. If possible, the patient immobilization device should also be used for the MRI scan. Image registration and fusion applications, if available, should be used to help in the delineation of target volumes. Image registration should be performed in a region of interest encompassing the GTV, skull base, brainstem, and optic chiasm.

6.3.3 The GTV and CTV (see Section 6.4), and normal tissues must be outlined on all CT slices in which the structures exist.

6.4 Treatment Planning/Target Volumes

The definition of volumes will be in accordance with the 1993 ICRU Report #50: Prescribing, Recording and Reporting Photon Beam Therapy.

6.4.1 *The Gross Tumor Volume (GTV)* is defined as all known gross disease determined from CT, MRI, clinical information, and endoscopic findings. Grossly positive lymph nodes are defined as any lymph nodes > 1 cm or nodes with a necrotic center. It is strongly encouraged that the

radiation oncologist outline the radiologic extent of the primary tumor and neck nodes along with a neuro-radiologist. Whenever possible, it is recommended that the diagnostic images be fused to the planning CT scan image dataset to more accurately define the GTV. To further subdivide the GTV, gross disease at the primary site is designated as GTV-P and clinically involved gross lymph nodes are designated GTV-N.

6.4.2 *The Clinical Target Volume (CTV)*: See Sections 6.4.2.1-6.4.2.3 for delineation details. For the split beam technique [3D-CRT or IMRT asymmetrically matched to low neck AP or AP/PA field(s)], two separate CTV's will be defined, namely CTV_{70} and $CTV_{59.4}$. In terms of the GTV (GTV-P and GTV-N), a margin of ≥ 5 mm should be given circumferentially around the GTV (GTV-P and GTV-N) and this volume will be called the CTV_{70} (CTV_{70}-P and CTV_{70}-N).* This margin can be reduced to as low as 1 mm for tumors in close proximity to critical structures, e.g., tumors invading the skull base and abutting the brain stem. For regions deemed to be at high risk for microscopic disease, all potential routes of spread for primary and nodal GTVs should be delineated by the treating radiation oncologist. This is known as CTV for high risk subclinical disease or $CTV_{59.4}$.*

The low anterior neck can be separately treated with conventional AP or AP/PA portal(s). This volume is defined as a low risk subclinical region and can receive a lower dose, 50 Gy. For a single 3D-CRT or IMRT plan in which the primary site, high risk subclinical and the low risk subclinical disease are being treated with one 3D-CRT or IMRT plan, CTV_{70} and $CTV_{59.4}$ are defined exactly the same as in the split beam technique. However, the lower risk subclinical disease will receive 54 Gy at 1.64 Gy per fraction instead of 50 Gy. The lower risk subclinical disease is called CTV_{54}. At the discretion of the treating physician, a CTV_{63} for small volume lymph node disease is allowed, if the patient is treated with a single 3D-CRT or IMRT plan.

Note: In all directions, the margin between each GTV and its CTV will be at least 5 mm except when the clivus is completely infiltrated with GTV and is adjacent to the brain stem. In those situations, **this margin can be as small as 1 mm**. CTV margins may also be limited to exclude bone or air NOT at risk for subclinical disease.

6.4.2.1 To further clarify the definitions, CTV_{70} should include the gross disease at the primary disease site or any grossly involved lymph nodes (CTV_{70}-P and CTV_{70}-N), except when an intermediate 63 Gy dose may be appropriate for small volume nodal disease, CTV_{63}-N when using a single 3D-CRT or IMRT plan.

6.4.2.2 To further define the high risk subclinical region at the primary disease site, $CTV_{59.4}$-P includes the entire nasopharynx, anterior 1/2 to 2/3 of the clivus (entire clivus, if involved), skull base (foramen ovale and rotundum bilaterally must be included for all cases), pterygoid fossae, parapharyngeal space, inferior sphenoid sinus (in T3-T4 disease, the entire sphenoid sinus) and posterior fourth to third of the nasal cavity and maxillary sinuses (to ensure pterygopalatine fossae coverage). The cavernous sinus should be included in high-risk patients (T3, T4, bulky disease involving the roof of the nasopharynx).

Note: The outer most boundary of $CTV_{59.4}$-P should be at least 10 mm from the GTV-P. Typically, it is larger as coverage of the regions defined above is necessary.

6.4.2.3 Regarding the high risk lymph nodal regions, $CTV_{59.4}$-N includes:

a. Upper deep jugular (junctional, parapharyngeal): bilaterally;
b. Subdigastric (jugulodigastric) [level II]: bilaterally;
c. Midjugular (level III): bilaterally;
d. Low jugular and supraclavicular (level IV): bilaterally;
e. Posterior cervical (level V): bilaterally;
f. Retropharyngeal: bilaterally;
g. Submandibular (level I): bilaterally.**

Note: The outer most boundary of the $CTV_{59.4}$-N should be at least 10 mm away from the GTV-N. This margin should at least be 10 mm from the retropharyngeal lymph nodes, except when the CTV is in air in neck region or in bone.

**Bilateral IB lymph nodes can be spared if patient is node negative. The treatment of level IB may result in the delivery of clinical significant radiation doses to normal structures such as the floor of mouth, mandible and upper pharyngeal mucosa above the hyoid. At the discretion of the treating Radiation Oncologist, level IB may also be spared or limited to the anterior border of the submandibular gland in low risk node positive patients. Patients presenting with isolated retropharyngeal nodes or isolated level IV nodes are considered low risk for level IB involvement. Treatment of level IB should be considered in node negative

patients with extensive involvement of the hard palate, nasal cavity or maxillary antrum. (Uninvolved lower neck nodal regions may be treated to 54 Gy/33 fractions, if a single 3D-CRT or IMRT technique is considered or 50 Gy/25 fractions, if the split beam 3D-CRT or IMRT technique is considered (see Section 6.4.2).

Note: The consensus guideline for head and neck cancer is for **NODE NEGATIVE** patients only. One can use this guideline to treat the appropriate nodal levels only for **NODE NEGATIVE** patients.

6.4.2.4 Examples of the definition of the appropriate nodal groups can be found at the RTOG Image-Guided Therapy Center (ITC) web site at http://atc.wustl.edu..

6.4.3 A separate planning Target Volume (PTV) will provide a margin around the CTV's to compensate for the variabilities of treatment set up and internal organ motion. Studies should be implemented by each institution to define the appropriate magnitude of the uncertain components of the PTV. Until the results of that study are available, a minimum of 5 mm around the CTV's is required in all directions to define each respective PTV (PTV_{70}, PTV_{63}, $PTV_{59.4}$, PTV_{54}). Careful consideration should be made when defining the superior and inferior margins in three dimensions. If the treating physician feels that the set-up at his or her institution is fairly accurate, it is appropriate to use the institution's respective PTV margin for the various CTV's. **Note that at any given point, the margin from the GTV at the primary site to the $PTV_{59.4}$ should be at least 15mm.**

If the delineation is done correctly, the results should be as follows:

Example 1: GTV-P + 5mm = CTV_{70}-P; GTV-N + 5 mm = CTV_{70}-N; $CTV_{59.4}$-P includes CTV_{70}-P and $CTV_{59.4}$-N includes CTV_{70}-N but the outermost boundary must be at least 10 mm away from GTV-P and GTV-N. PTV_{70} will be 5 mm at least away from CTV_{70}. $PTV_{59.4}$ will be at least 15 mm away from GTV at the primary site and from the gross nodes including the retropharyngeal region where the margin is at least 15 mm.

Example 2: GTV-P or GTV-N = CTV_{70}-P or CTV_{70}-N; $CTV_{59.4}$-P or $CTV_{59.4}$-N includes CTV_{70}-P or CTV_{70}-N but the outermost boundary again must be at least 10 mm away from the GTV at the primary site and from the grossly involved lymph nodes including the retropharyngeal region. PTV_{70}-P and PTV_{70}-N will be 5 mm at least away from CTV_{70}. $PTV_{59.4}$-P will be at least 15 mm away from GTV-P and $PTV_{59.4}$-P is at least 15 mm away from the GTV-N including the retropharyngeal region.

Although not shown as an example, the treating physician has the option to treat small volume lymph nodes to 63 Gy. **The margins at the primary and the nodal sites are crucial to ensure no marginal misses**. The margins can be as small as 1 mm when disease is adjacent to the brain stem.

6.4.4 *Planning*

The treatment plan used for each patient treated with either 3D-CRT or IMRT will be based on an analysis of the volumetric dose, including dose-volume histogram (DVH) analyses of the PTVs and critical normal structures. A three dimensional or an "inverse" planning using computerized optimization should be used. The treatment aim will be the delivery of radiation to the PTVs and the exclusion of non-involved tissue.

6.5 Critical Structures

NOTE: All dose constraints below should be met whether the patient undergoes 3D-CRT or IMRT techniques.

6.5.1 *Critical Normal Structures*

Surrounding critical normal structures, including the brainstem, spinal cord, optic nerves, chiasm, parotid glands, pituitary, temporo-mandibular (T-M) joints and middle and inner ears, skin (in the region of the target volumes), oral cavity, mandible, eyes, lens, temporal lobes, brachial plexus, esophagus (including postcricoid pharynx) and glottic larynx should be outlined.

Physicians should assist the planner in identifying the critical normal structures. If planning organ at risk volumes (PRVs) are used, the spinal cord PRV will be defined as a three-dimensional margin at least 5 mm larger than the spinal cord to ensure that the PRV margin is at least 5 mm from any portion of the spinal cord. The brainstem PRV, chiasm PRV, and optic

nerve PRV will be defined as at least 1 mm larger in all directions than the corresponding structure. The normal tissues will be contoured and considered as solid organs. The tissue within the skin surface and outside all other critical normal structures and PTVs is designated as unspecified tissue.

DVH's must be generated for all critical normal structures, any corresponding PRVs, and the unspecified tissues. Institutions that use PRVs must clearly define them as such. Dose constraints are given below:

**Structure**	**true structure constraint**	**PRV constraint**
Brainstem	54 Gy max dose	no more than 1% to exceed 60 Gy
Spinal Cord	45 Gy max dose	no more than 1% to exceed 50 Gy
Optic Nerves, Chiasm	50 Gy max dose	54 Gy max dose
Mandible, TM joint	70 Gy, if not possible then no more than 1cc to exceed 75 Gy	
Brachial Plexus	66 Gy max dose	

Unspecified tissue outside the targets: No more than 5% of unspecified tissue can receive greater than 70 Gy or more and no more than 1% or 1cc of unspecified tissue can receive 77 Gy or more. **Participants are strongly encouraged to remain within these limits.**

6.5.2 The method used for tissue heterogeneity calculations shall be reported. The density corrected dose distributions shall be calculated and submitted to the RTOG ITC. The dose prescription is to be based on a dose distribution corrected for heterogeneities.

6.5.3 *Planning Goals: Salivary Glands and Other Normal Structures*

6.5.3.1 Parotid glands: Mean dose < 26 Gy (should be achieved in at least one gland) or at least 20 cc of the combined volume of both parotid glands will receive < 20 Gy or at least 50% of one gland will receive < 30 Gy (should be achieved in at least one gland).

Submandibular/sublingual glands and oral cavity: Reduce the dose as much as possible.

6.5.3.2 Other normal structures: Dose constraints for other normal structures are suggested below. While these dose objectives are recommendations and the constraints should not compromise the GTV or CTV coverage, every effort should be made to stay within the normal tissue dose guidelines stipulated in this protocol.

Oral cavity (excluding PTV's)	Mean dose less than 40 Gy
Each cochlea	No more than 5% receives 55 Gy or more
Eyes	Max dose less than 50 Gy
Lens	Max dose less than 25 Gy
Glottic Larynx	Mean dose less than 45 Gy
Esophagus, Postcricoid pharynx	Mean dose less than 45 Gy

6.5.4 *Planning Priorities*

Critical normal structure constraints followed by the prescription goals are the most important planning priorities. The priorities in addressing the protocol aims and constraints will be in the following order:
1) Critical Normal Structure Constraints (Section 6.5);
2) Dose Specifications (Section 6.1);
3) Planning Goals: Salivary glands (Section 6.5.3);
4) Planning Goals: Other normal structures (Section 6.5.3).

6.6 Documentation Requirements

Verification and orthogonal films or images are required. For all forms of 3D-CRT or IMRT dose delivery, orthogonal films or images that localize the isocenter placement shall be obtained. The length of the treatment field shall be indicated on these films.

6.7 Compliance Criteria

6.7.1 *Quality Assurance of Target Volumes and Critical Structure Volumes*

The ITC will facilitate the review of all PTVs and designated critical structures on all cases submitted from each institution.

6.7.2 *Quality Assurance of Field Placement*

IMRT: The ITC will review one set of orthogonal (anterior posterior and lateral) prescription images for isocenter (or IMRT reference point) localization for each group of concurrently treated beams for the first five cases submitted by each institution. The digital reconstructed radiographs (DRRs) from the treatment planning program or, alternatively, a simulation verification radiograph shall be submitted for evaluation except where geometrically impractical.

6.7.3 *Quality Assurance of Dose Distribution*

The ITC will display, and compare with hard copies, isodose distributions through the planning target volume to verify correct digital submission and conversion. The ITC will compare the submitted digital dose-volume histograms (DVHs) for the PTVs, the designated critical structures, and unspecified tissues with DVHs calculated by the ITC.

6.7.4 Each submitted treatment plan will be judged as follows: **(4/21/08)**

PTV	No Variation	Minor Variation
PTV_{70}	1. 95% of any PTV_{70} is at or above 70 Gy<br>2. 99% of PTV_{70} is at or above 65.1 Gy<br>3. No more than 20% of PTV_{70} is at or above 77 Gy<br>4. No more than 5% of PTV_{70} is at or above 80 Gy<br>5. Mean dose ≤ 74 Gy	1. 95% of PTV_{70} is at or above 70 Gy<br>2. 97% of PTV_{70} is at or above 65.1Gy<br>3. No more than 40% of PTV_{70} is at or above 77 Gy<br>4. No more than 20% of PTV_{70} is at or above 80 Gy<br>5. Mean dose ≤ 76 Gy
PTV_{63} *(if applicable)*	1. 95% of any PTV_{63} is at or above 63 Gy<br>2. 99% of PTV_{63} is at or above 58.6 Gy<br>3. No more than 20% of PTV_{63} is at or above 77 Gy<br>4. No more than 5% of PTV_{63} is at or above 80 Gy	1. 95% of any PTV_{63} is at or above 58.6 Gy<br>2. No more than 40% of PTV_{63} is at or above 77 Gy<br>3. No more than 20% of PTV_{63} is at or above 80 Gy
$PTV_{59.4}$	1. 95% of any $PTV_{59.4}$ is at or above 59.4 Gy<br>2. 99% of $PTV_{59.4}$ is at or above 55.2 Gy<br>3. No more than 20% of $PTV_{59.4}$ is at or above 77 Gy<br>4. No more than 5% of $PTV_{59.4}$ is at or above 80 Gy	1. 95% of $PTV_{59.4}$ is at or above 55.2 Gy<br>2. No more than 40% of $PTV_{59.4}$ is at or above 77 Gy<br>3. No more than 20% of $PTV_{59.4}$ is at or above 80 Gy

PTV_{54} (*if applicable*)	1. 95% of any PTV_{54} is at or above 54 Gy<br>2. 99% of PTV_{54} is at or above 50.2 Gy<br>3. No more than 20% of PTV_{54} is at or above 65.3 Gy<br>4. No more than 5% of PTV_{54} is at or above 68.3 Gy	1. 95% of PTV_{54} is at or above 50.2 Gy<br>2. No more than 40% of PTV_{54} is at or above 65.3 Gy<br>3. No more than 20% of PTV_{54} is at or above 68.3 Gy

6.8 R.T. Quality Assurance Reviews

The Radiation Oncology Study Chairs, Nancy Lee, MD, and Adam Garden, MD will remotely perform an RT Quality Assurance Review after complete data for the first 16 cases enrolled has been received. Drs. Lee and Garden will perform the next review after complete data for the next 16 cases enrolled has been received. The final cases will be reviewed within 3 months after this study has reached the target accrual or as soon as complete data for all cases enrolled has been received, whichever occurs first.

6.9 Radiation Adverse Events (2/16/11)

As of April 1, 2011, this study will utilize the Common Terminology Criteria for Adverse Events (CTCAE) version 4 for grading of all adverse events reported via AdEERS. All RTOG case report forms will continue to use CTCAE, v. 3.0. A copy of the CTCAE v. 4 can be downloaded from the CTEP home page (http://ctep.cancer.gov) or the RTOG web site, http://www.rtog.org/members/toxicity/main.html. All appropriate treatment areas should have access to a copy of the CTCAE v. 4.

Grade 3-4 therapy-induced mucositis and/or dysphagia, which are enhanced by cisplatin, are expected to develop in about two thirds of patients. Nutritional evaluation prior to the initiation of therapy for a prophylactic gastrostomy (PEG) tube placement is highly recommended. Placement of a feeding tube should be recorded, as should use of a feeding tube during and after treatment (e.g., greater than or less than 50% of nutrition by tube). Other common radiation adverse events include: fatigue, weight loss, regional alopecia, xerostomia, hoarseness, transient ear discomfort, dysgeusia, and skin erythema and desquamation within the treatment fields.

Less common long-term treatment adverse events include: hypothyroidism, loss of hearing, chronic swallowing dysfunction requiring permanent feeding tube, and cervical fibrosis. Much less common radiation adverse events include: mandibular osteoradionecrosis (< 5% incidence with attention to the dental recommendations provided in Appendix VI), and cervical myelopathy (< 1% with restriction of spinal cord dose to ≤ 45 Gy).

6.9.1 *Treatment Interruptions*

Interruptions in radiotherapy may be necessitated by skin reaction, mucositis, ulceration, edema, or other acute complication. The reason for and the length of any such interruption must be documented. If the sum total of such interruptions exceeds five normally-scheduled treatment days, the treatment may be considered in major violation of protocol. Radiation therapy will be continued without interruption if at all possible. Should confluent mucositis, moist desquamation unresponsive to topical dressings, or severe stomatitis resulting in weight loss greater than 15% occur, radiation may be interrupted in order to relieve morbidity. The use of tube feedings in this situation is encouraged; it is anticipated to minimize treatment interruptions.

6.10 Radiation Adverse Event Reporting (5/7/07)

See AdEERS Expedited Reporting Requirements in Section 7.9.

7.0 DRUG THERAPY

Institutional participation in chemotherapy studies must be in accordance with the Medical Oncology Quality Control guidelines stated in the RTOG Procedures Manual.

Protocol treatment must begin within 2 weeks after registration.

7.1 Chemotherapy and Biologic Therapy, Concurrent with Radiation Therapy

7.1.1 *Dose definition* **(11/7/07)**

- Cisplatin: 100 mg/m^2/day, days 1, 22, and 43
- Bevacizumab: 15 mg/kg/day, days 1, 22 and 43

Bevacizumab can be given first while the pre-cisplatin hydration is being administered, but the order of administration of cisplatin and bevacizumab is up to the discretion of the treating oncologist.

The dosing date for cisplatin and bevacizumab may be delayed for up to 3 days for logistical reasons (e.g., holiday, patient scheduling conflict), or per dose modification guidelines outlined in Section 7.7. The dates of subsequent cisplatin and bevacizumab treatments will be adjusted so that recycling times are not shortened (e.g., if treatment is delayed to day 24, the next cycle will be planned for day 45). **Note:** If cisplatin is delayed, bevacizumab also should be held in order to keep the chemotherapy and bevacizumab administrations on the same day.

7.1.2 *Technique of administration* **(5/7/07)**

7.1.2.1 Cisplatin Administration Guidelines

Note: Many institutions will have standard guidelines for the administration of cisplatin at the doses used in this study. For purposes of this protocol, individual investigators may use these local guidelines for cisplatin administration. One possible approach is outlined below, but this may need to be modified based on local guidelines and patient related factors (e.g., the substitution of normal saline in diabetic patients). Similarly, the anti-emetic regimen for this combination is to be determined by the local investigator. Practice guidelines for this purpose from the American Society of Clinical Oncology can be accessed at http://www.jco/cgi/reprint/JCO.2006.06.959v1.pdf.

Patients will be pre-hydrated with two liters of 5% D-1/2 NS and 40 mEq KCl/L. This is to be followed by 12.5 gm mannitol immediately before administration of cisplatin.

Cisplatin is given intravenously over 20-30 minutes followed by 1L of 5% D-1/2 NS and 40 mEq KCl and 25 gm mannitol over four hours, followed by 1L 5% D-1/2 NS and 40 mEq KCl and 8 mEq MgSO4 over eight hours.

Patients should receive at least 3L of fluids over the ensuing 24 hours, either parenterally or orally. Electrolytes and serum creatinine should be monitored during treatment with high-dose cisplatin, and a follow-up creatinine within 24-48 hours of the cisplatin dose is recommended.

See Section 7.6 for dose modification details.

Note: Bevacizumab can be given while the pre-cisplatin hydration is being administered, but the order of administration of cisplatin and bevacizumab is up to the discretion of the treating oncologist.

7.1.2.2 Bevacizumab Administration Guidelines

The first dose will be delivered intravenously over 90 +/- 15 minutes. If the first infusion is tolerated without any infusion-associated adverse events such as fever and/or chills, the second infusion may be delivered over 60 +/- 10 minutes. If this is well tolerated, the third infusion may be delivered over 30 +/- 10 minutes.

Routine premedication is not required for the first dose of bevacizumab, although it could be given at physician's discretion. Anaphylactic precautions should be observed during bevacizumab administration. If an infusion-associated reaction is experienced, the patient may be premedicated for the next dose with acetaminophen, diphenhydramine, steroids or other medications. If the next infusion is well tolerated with pre-medication, the subsequent infusion time may then be decreased by 30+/- 10 minutes as long as the patient continues to be premedicated. If the patient continues to experience an infusion-associated adverse event with the 60-minute infusion, all subsequent doses should be given over 90+/- 15 minutes. Similarly, if the patient experiences an infusion-associated adverse event with the 30-minute infusion, all subsequent doses should be given over 60 +/- 10 minutes.

To insure complete delivery of bevacizumab, the IV infusion line must be flushed with 0.9% sodium chloride. The following are two recommended methods for flushing the bevacizumab IV infusion line:

1. When the bevacizumab infusion is complete, add an additional 50mL of 0.9% sodium chloride for injection to the bevacizumab infusion bag. Continue the infusion until a volume equal to that of the volume contained in the tubing has been administered.
2. Replace the empty bevacizumab infusion bag with a 50mL bag of 0.9% sodium chloride for injection and infuse a volume equal to the volume contained in the tubing.

Please note: the flush is not included in the total recommended infusion times.

Special Cautions/Safety Issues:
Prior to each treatment, the patient should be carefully assessed with special attention to blood pressure, proteinuria, bleeding and cardiovascular events. Modification of dose or discontinuation of therapy should be considered if the patient experiences uncontrolled hypertension and/or a UPC ratio of ≥ 3.5.

See Section 7.7.6.4 for treatment modification details.

7.1.3 *Duration of treatment*

- Cisplatin: given every 3 weeks (21 days) for 3 cycles
- Bevacizumab: given every 3 weeks (21 days) for 3 cycles

7.2 Adjuvant Chemotherapy and Biologic Therapy, After Radiation Therapy (11/7/07)

7.2.1 *Dose definition* **(5/7/07)**

- Cisplatin: 80 mg/m^2/day, days 64, 85, 106
- 5-fluorouracil: 1,000 mg/m^2/day, days 64-67, 85-88, 106-109
- Bevacizumab: 15 mg/kg/day, days 64, 85, 106

The dosing date for cisplatin, 5-fluorouracil, and bevacizumab may be delayed for up to 3 days for logistical reasons (e.g., holiday, patient scheduling conflict), or per dose modification guidelines outlined in Section 7.7. The dates of subsequent cisplatin, 5-fluorouracil, and bevacizumab treatments will be adjusted so that recycling times are not shortened (e.g., if treatment is delayed to day 87, the next cycle will be planned for day 108). **Note:** If cisplatin and/or 5-fluorouracil is delayed, bevacizumab also should be held in order to keep the chemotherapy and bevacizumab administrations on the same day. Patients will not make up missed doses of chemotherapy or bevacizumab.

7.2.2 *Technique of administration*

7.2.2.1 Cisplatin Administration Guidelines
See Section 7.1.2.1.

7.2.2.2 5-fluorouracil Administration Guidelines **(4/21/08)**
5-fluorouracil is given intravenously in 5% glucose in ½ NS as a 96 hour continuous infusion after the cisplatin is completed on the first day of the chemotherapy cycle. However, institutions may follow their standard guidelines for the administration of 5-FU at the dose specified in this study (e.g., the substitution of normal saline for diabetic patients).

See Section 7.6 for dose modification details.

7.2.2.3 Bevacizumab Administration Guidelines
See Section 7.1.2.2; as this will not be the first dose of bevacizumab, infusion duration may be less than 90 minutes based on the patient's tolerance of prior dosing.

7.2.3 *Duration of treatment*

- Cisplatin: given every 3 weeks (21 days) for 3 cycles
- 5-fluorouracil: given every 3 weeks (21 days) for 3 cycles
- Bevacizumab: given every 3 weeks (21 days) for 3 cycles

7.3 Cisplatin (Cis-Diamminedichloroplatinum, DDP) **[11/7/07]**
Refer to package insert for additional information.

7.3.1 *Formulation*: Cisplatin is commercially supplied as a lyophilized powder in 10 mg and 50 mg vials. The drug should be reconstituted using 10 mL and 50 mL respectively of sterile water for injection, USP, to yield a concentration of 1 mg/mL. Preconstituted vials of 100 mg also are available. Once the multidose vial has been entered, the remaining cisplatin is stable for 28 days when protected from light. For further information, see package insert.

7.3.2 *Mechanism of Action:* The mechanism of action of DDP has not been clearly elucidated. However, preliminary studies have indicated that the most likely mechanism of antitumor action of this drug resides in its ability to inhibit DNA synthesis and to a lesser degree, RNA and

protein synthesis. It has also been shown that DDP binds to DNA and produces inter-strand cross-links. Also DDP is not phase-sensitive and its cytotoxicity is similar in all phases of the cell cycle.

7.3.3 *Preparation*: Reconstituted solution of cisplatin is stable for 20 hours when stored at 27°C and should be protected from light if not used within 6 hours.

7.3.4 *Administration*: Intravenous.

7.3.5 *Adverse Events*

The following toxicities are anticipated:

- Hematologic: Myelosuppression, often with delayed erythrosuppression; rarely, acute leukemia
- Gastrointestinal: Nausea, vomiting, anorexia, loss of taste;
- Dermatologic: Alopecia;
- Renal: Elevation of BUN, creatinine and impairment of endogenous creatinine clearance, as well as renal tubular damage which appears to be transient); hyperuricemia; much more severe and prolonged toxicity has been observed in patients with abnormal or obstructed urinary excretory tracts;
- Hepatic: Hypomagnesemia, hypokalemia, hypocalcemia,
- Neurologic: Restlessness; involuntary movements; loss of coordination; seizures; peripheral neuropathy;
- Allergic: Flushing, bronchoconstriction, tachycardia, hypotension;
- Other: Ototoxicity (with hearing loss which initially is in the high-frequency range, as well as tinnitus); muscle cramps; weakness

7.3.6 *Storage:* Intact vials of the dry powder and the aqueous injection should be stored at room temperature (15-25°C) and protected from light; the vials and injection should not be refrigerated.

7.3.7 *Supply*: Commercially available.

7.4 5-Fluorouracil

Refer to package insert for additional information.

7.4.1 *Other Names*

5-FU, Adrucil, Efudex.

7.4.2 *Formulation*

5-FU is supplied as a colorless-to-faint-yellow solution in 10-mL single-use vials. Each 10 mL of solution contains 500 mg 5-FU with pH adjusted to approximately 9.2 with sodium hydroxide. 5-FU is commercially available as a multisource product. For this study, locally obtained commercial supplies of 5-FU should be used. For further information, see package insert.

7.4.3 *Administration*

May be administered via the following: *i.v.* push, *i.v.* continuous infusion, arterial infusion, intracavitary, intraperitoneally, topically, or orally mixed in water, grape juice, or carbonated beverage.

7.4.4 *Adverse Events*

The following toxicities are anticipated:

- Hematologic: Leukopenia, thrombocytopenia, anemia (can be dose limiting, less common with continuous infusion);
- Dermatologic: Dermatitis, nail changes, hyperpigmentation, Hand-Foot Syndrome with protracted infusions, alopecia;
- Gastrointestinal: Nausea, vomiting, anorexia, diarrhea (can be dose limiting); mucositis (more common with 5-day infusion, occasionally dose limiting); severe, cholera-like diarrhea which can be fatal when given with leucovorin;
- Neurologic: Cerebellar Syndrome (headache and cerebellar ataxia);
- Cardiac: Angina, noted with continuous infusion;
- Ophthalmic: Eye irritation, nasal discharge, watering of eyes, blurred vision.

7.4.5 *Drug Interactions*

7.4.5.1 Cimetidine: Because cimetidine can decrease the clearance of 5-FU, patients should not enter on this study until the cimetidine is discontinued. Ranitidine or a drug from another anti-ulcer class can be substituted for cimetidine, as necessary.

7.4.5.2 Allopurinol: Oxypurinol, a metabolite of allopurinol, can potentially interfere with 5-FU anabolism via orotate phosphoribosyltransferase. Although this was originally used as a strategy to protect normal tissues from 5-FU-associated toxicity, further laboratory studies suggested possible antagonism of the anticancer activity of 5-FU in some tumor models. If a patient is receiving allopurinol, the need for taking this medicine should be ascertained. If

possible, allopurinol should be discontinued prior to starting on this regimen, and another agent substituted for it.

7.4.6 *Storage*
Stable for prolonged periods of time at room temperature, if protected from light. Inspect for precipitate; if apparent, agitate vial vigorously or gently heat to not greater than 140°F in a water bath. Do not allow to freeze.

7.4.7 *Supply*
Commercially available.

7.5 Bevacizumab (rhuMAb VEGF, Avastin™) [NSC #7048865; IND 7921] **(4/21/08)**
For further information, refer to the package insert.

7.5.1 *Formulation*
Bevacizumab is a recombinant humanized anti-VEGF monoclonal antibody. consisting of 93% human and 7% murine amino acid sequences. The antibody consists of a human IgG1 framework and the antigen-binding complementarity-determining regions from the murine anti-VEGF MAb A.4.6.1;[17,20,23] approximate molecular weight is 149,000 daltons.

7.5.2 *Availability* **(4/21/08)**
Bevacizumab is supplied as a clear to slightly opalescent, sterile liquid ready for parenteral administration in two vial sizes: (also see Section 7.6.8 for preparation guidelines)

- Each 100 mg (25 mg/mL – 4 mL fill) glass vial contains bevacizumab with phosphate, trehalose, polysorbate 20, and Sterile Water for Injection, USP.
- Each 400 mg (25mg/ml – 16 mL fill) glass vial contains bevacizumab with phosphate, trehalose, polysorbate 20, and Sterile Water for Injection, USP.

7.5.3 *Pharmacokinetics*
Bevacizumab blocks the binding of vascular endothelial growth factor (VEGF) to its receptors resulting in inhibition of angiogenesis. Neutralization of VEGF by A.4.6.1 or bevacizumab has been shown to inhibit the VEGF-induced proliferation of human endothelial cells *in vitro* and decrease microvessel density and interstitial pressure in tumor xenografts *in vivo*. In patients, preliminary results from a neoadjuvant trial in rectal cancer demonstrated a decrease in blood perfusion/permeability and interstitial fluid pressure in tumors after one dose of bevacizumab.[36]

7.5.4 *Storage and Stability*
Upon receipt, bevacizumab should be refrigerated (2E to 8E C). Do not freeze. Do not shake. Shelf-life studies of rhuMAb VEGF are ongoing. The sterile single use vials contain no antibacterial preservatives. Therefore, vials should be discarded 8 hours after initial entry. Once diluted in 0.9% sodium chloride, solutions of bevacizumab must be administered within 8 hours.

7.5.5 *Accountability and Supply*
The Principal Investigator (or authorized designee, who the investigator has listed on their most recent Supplemental Investigator Data Form [IDF] on file with the PMB) at each participating institution may request bevacizumab, from NCI's Pharmaceutical Management Branch (PMB). PMB will not provide drug to a site until the site has registered the patient. The updated version (11/10/03) of each institution's Drug Authorization Review and Tracking System (DARTS) will require selecting a designee from the individuals listed on the IDF. The information on the IDF is linked to the investigator during the annual investigator registration process. This process must be completed before a drug order can be entered for that investigator. Any changes necessary to this information will require updating the first two pages of the IDF, having been signed by the investigator, and returning it to the PMB via fax at 301-402-4870. Questions about the process should be directed to the PMB at 301-496-5725 Monday through Friday from 8:30 – 4:30 Eastern Time. PMB policy requires that drug be shipped directly to the institution where the patient is to be treated. PMB does not permit the transfer of agents between institutions unless prior approval from PMB is obtained. Completed Clinical Drug Requests (NIH-986) should be submitted to the PMB by fax (301) 480-4612 or mailed to the Pharmaceutical Management Branch, CTEP, DCTD, NCI, 9000 Rockville Pike, EPN, Rm. 149, Bethesda, MD 20892.

7.5.6 *Drug Inventory Records*
The Investigator, or a responsible party designated by the investigator, must maintain a careful record of the inventory and disposition of all drugs received from DCTD, using the NCI Drug Accountability Record Form (see the NCI Investigators Handbook for Procedures for Drug Accountability and Storage).

7.5.7 *Route of Administration*
Intravenous

7.5.8 *Preparation*
Vials contain no preservatives and are intended for single use only. Place the calculated dose in 100 mL of 0.9% sodium chloride for injection.

7.5.9 *Adverse Events* **(11/7/07)**
Comprehensive Adverse Events and Potential Risks List (CAEPR) for Bevacizumab (NSC 704865)

The Comprehensive Adverse Event and Potential Risks list (CAEPR) provides a single list of reported and/or potential adverse events (AE) associated with an agent using a uniform presentation of events by body system. In addition to the comprehensive list, a subset, the Agent Specific Adverse Event List (ASAEL), appears in a separate column and is identified with ***bold*** and ***italicized*** text. This subset of AEs (ASAEL) contains events that are considered 'expected' for expedited reporting purposes only. Refer to the 'CTEP, NCI Guidelines: Adverse Event Reporting Requirements' http://ctep.cancer.gov/reporting/adeers.html for further clarification. The CAEPR does not provide frequency data; refer to the Investigator's Brochure for this information. Below is the CAEPR for Bevacizumab.

Version 1.2, June 19, 2007[1]

Category (Body System)	Adverse Events with Possible Relationship to Bevacizumab (CTCAE v3.0 Term)	'Agent Specific Adverse Event List' (ASAEL)
ALLERGY/IMMUNOLOGY		
	Allergic reaction/hypersensitivity (including drug fever)	***Allergic reaction/hypersensitivity (including drug fever)***
	Allergic rhinitis (including sneezing, nasal stuffiness, postnasal drip)	***Allergic rhinitis (including sneezing, nasal stuffiness, postnasal drip)***
BLOOD/BONE MARROW		
	Hemoglobin	***Hemoglobin***
	Leukocytes (total WBC)	***Leukocytes (total WBC)***
	Neutrophils/granulocytes (ANC/AGC)	***Neutrophils/granulocytes (ANC/AGC)***
CARDIAC ARRHYTHMIA		
	Supraventricular arrhythmia NOS	***Supraventricular arrhythmia NOS***
	Ventricular fibrillation	
CARDIAC GENERAL		
	Cardiac ischemia/infarction	***Cardiac ischemia/infarction***
	Cardiac troponin I (cTnI)	
	Hypertension	***Hypertension***
	Hypotension	
	Left ventricular diastolic dysfunction	
	Left ventricular systolic dysfunction	
CONSTITUTIONAL SYMPTOMS		
	Fatigue (asthenia, lethargy, malaise)	***Fatigue (asthenia, lethargy, malaise)***
	Fever (in the absence of neutropenia, where neutropenia is defined as ANC <1.0 x 10^9/L)	***Fever (in the absence of neutropenia, where neutropenia is defined as ANC <1.0 x 10^9/L)***
	Rigors/chills	***Rigors/chills***
	Weight loss	
DERMATOLOGY/SKIN		
	Pruritus/itching	***Pruritus/itching***
	Rash/desquamation	***Rash/desquamation***
	Ulceration	
	Urticaria (hives, welts, wheals)	***Urticaria (hives, welts, wheals)***
	Wound complication, non-infectious	
GASTROINTESTINAL		
	Anorexia	***Anorexia***
	Colitis	
	Constipation	***Constipation***
	Diarrhea	***Diarrhea***
	Fistula, GI - Select	
	Heartburn/dyspepsia	***Heartburn/dyspepsia***
	Ileus (functional obstruction of bowel, i.e.,	

Category (Body System)	Adverse Events with Possible Relationship to Bevacizumab (CTCAE v3.0 Term)	'Agent Specific Adverse Event List' (ASAEL)
	neuroconstipation)	
	Leak (including anastomotic), GI: large bowel	
	Mucositis/stomatitis (functional/symptomatic) - Select	***Mucositis/stomatitis (functional/symptomatic) - Select***
	Nausea	***Nausea***
	Perforation, GI - Select	
	Ulcer, GI - Select	
	Vomiting	***Vomiting***
HEMORRHAGE/BLEEDING		
	Hemorrhage, GI - Select	***Hemorrhage GI - Select***
	Hemorrhage, CNS	***Hemorrhage, CNS***
	Hemorrhage, GU: vagina	***Hemorrhage, GU: vagina***
	Hemorrhage, pulmonary/upper respiratory: lung	***Hemorrhage, pulmonary/upper respiratory: lung***
	Hemorrhage, pulmonary/upper respiratory: nose	***Hemorrhage, pulmonary/upper respiratory: nose***
INFECTION		
	Infection with normal ANC or Grade 1 or 2 neutrophils - Select	
	Infection with normal ANC or Grade 1 or 2 neutrophils - Select (pelvis, peritoneal cavity, rectum, scrotum, skin, wound)	
METABOLIC/LABORATORY		
	Alkaline phosphatase	***Alkaline phosphatase***
	ALT, SGPT (serum glutamic pyruvic transaminase)	***ALT, SGPT (serum glutamic pyruvic transaminase)***
	AST, SGOT (serum glutamic oxaloacetic transaminase)	***AST, SGOT (serum glutamic oxaloacetic transaminase)***
	Bilirubin (hyperbilirubinemia)	***Bilirubin (hyperbilirubinemia)***
	Creatinine	
	Proteinuria	***Proteinuria***
NEUROLOGY		
	CNS cerebrovascular ischemia	***CNS cerebrovascular ischemia***
	Dizziness	***Dizziness***
	Neurology - Other: (Leukoencephalopathy syndrome including reversible posterior leukoencephalopathy syndrome [RPLS])	
PAIN		
	Pain - abdomen NOS	***Pain - abdomen NOS***
	Pain - chest/thorax NOS	***Pain - chest/thorax NOS***
	Pain - head/headache	***Pain - head/headache***
	Pain - joint	***Pain - joint***
	Pain - muscle	
	Pain - NOS	
PULMONARY/UPPER RESPIRATORY		
	Bronchospasm, wheezing	
	Cough	***Cough***
	Dyspnea (shortness of breath)	***Dyspnea (shortness of breath)***
	Fistula, pulmonary/upper respiratory - Select	
	Nasal cavity/paranasal sinus reactions	***Nasal cavity/paranasal sinus reactions***
	Voice changes/dysarthria (e.g., hoarseness, loss or alteration in voice, laryngitis)	***Voice changes/dysarthria (e.g., hoarseness, loss or alteration in voice, laryngitis)***
	Pulmonary/Upper Respiratory - Other (nasal-septal perforation)	
RENAL/GENITOURINARY		
	Fistula, GU - Select	
	Renal failure	
SYNDROMES		
	Cytokine release syndrome/acute infusion reaction	***Cytokine release syndrome/acute infusion reaction***
VASCULAR		
	Thrombosis/thrombus/embolism	***Thrombosis/thrombus/embolism***

Category (Body System)	Adverse Events with Possible Relationship to Bevacizumab (CTCAE v3.0 Term)	'Agent Specific Adverse Event List' (ASAEL)
	Visceral arterial ischemia (non-myocardial)	

[1]This table will be updated as the toxicity profile of the agent is revised. Updates will be distributed to all Principal Investigators at the time of revision. The current version can be obtained by contacting ADEERSMD@tech-res.com. Your name, the name of the investigator, the protocol and the agent should be included in the e-mail.

Also reported on bevacizumab trials but with the relationship to bevacizumab still undetermined:
BLOOD/BONE MARROW - Idiopathic thrombocytopenia purpura; platelets
CARDIAC GENERAL - Cardiac arrest; pericardial effusion; pulmonary hypertension
COAGULATION - DIC
DEATH - Sudden death (cause unknown)
DERMATOLOGY/SKIN - Hypopigmentation
GASTROINTESTINAL - Rectal abscess/necrosis; small bowel obstruction; taste alteration
METABOLIC/LABORATORY - Hyperglycemia; hypoglycemia; hypomagnesemia; hyponatremia
MUSCULOSKELETAL/SOFT TISSUE - Aseptic necrotic bone; gait/walking; myasthenia gravis
NEUROLOGY - Aseptic meningitis; confusion; peripheral neuropathy; seizure; syncope
OCULAR/VISUAL - Cataract; watery eye
PULMONARY/UPPER RESPIRATORY - ARDS; pneumonitis/pulmonary infiltrates; pneumothorax
RENAL/GENITOURINARY - Urinary frequency

Note: Bevacizumab in combination with other agents could cause an exacerbation of any adverse event currently known to be caused by the other agent, or the combination may result in events never previously associated with either agent.

7.6 Dose Modifications (11/7/07)

7.6.1 Patients will be examined and graded for subjective/objective evidence of developing toxicity according to the CTCAE, v. 3.0 each day that chemotherapy is administered and weekly while receiving radiotherapy. Treatment interruptions are allowed if there is symptomatic mucositis or skin reaction that, in the judgment of the clinician, warrants a break. The treatment is completed as per protocol for treatment breaks up to 14 days. If the break exceeds 14 days, the patient will be removed from protocol treatment. The patient will then complete treatment at the discretion of his/her physician but will be followed and included in the analysis.

7.6.2 There will be no dose escalation for concurrent cisplatin or for follow-up therapy with cisplatin and 5-FU.

7.6.3 Chemotherapy dosage modifications are based upon nadir counts and interim non-hematologic toxicities of the preceding cycle for cycles 2-6. The dose modifications for chemotherapy (below) are intended to be permanent (i.e., if the patient's dose is reduced to dose level -1, it remains at the reduced dose level).

7.6.4 *Dose Modifications for Hematologic Adverse Events During Concurrent Chemotherapy*

Cisplatin Dose Levels		
-2	-1	Starting Dose
60 mg/m^2	80 mg/m^2	100 mg/m^2

Chemotherapy must not be administered until the AGC ≥ 1,000 and platelets are ≥ 100,000. If not, delay one week. If the patient still has not recovered to these parameters in one week, discontinue cisplatin. Treatment dose is based upon the nadir counts as follows:

AGC		Platelets	Dose Reduction
≥ 1,500	and	≥ 75,000	Full dose
1,000 – 1,499	and/or	50,000 – 74,999	Decrease 1 level
< 1,000	and/or	< 50,000	Decrease 2 levels*

*If the patient is already at dose level 1, then decrease to dose level 2. If the patient is already at level 2, then discontinue concurrent cisplatin.

The third dose of cisplatin should be administered within 2 weeks of the end of radiation. If it cannot be administered in this timeframe, the dose should be held.

Note: Hematologic growth factors for neutropenia or anemia are not allowed during concurrent cisplatin and radiation treatment.

7.6.5 *Dose Modifications for Hematologic Adverse Events During Adjuvant Chemotherapy* **(4/21/08)**

Cisplatin Dose Levels		
-2	-1	Starting Dose
40 mg/m^2	60 mg/m^2	80 mg/m^2

5-FU Dose Levels		
-2	-1	Starting Dose
600 mg/m^2	800 mg/m^2	1,000 mg/m^2

Chemotherapy must not be administered until the AGC ≥ 1,000 and platelets are ≥ 100, 000. If not, delay one week. If the patient still has not recovered to these parameters in one week, discontinue adjuvant cisplatin and 5-fluorouracil. Treatment dose is based upon the nadir counts as follows:

AGC		Platelets	Dose Reduction
≥ 1,500	and	≥ 75,000	Full dose of both cisplatin and 5-FU
1,000 – 1,499	and/or	50,000 – 74,999	Decrease 1 level cisplatin; full dose of 5-FU
< 1,000	and/or	< 50,000	Decrease 2 dose levels*<br>Decrease l level 5-FU

*If patient has already been dose reduced, decreased two levels for cisplatin and decreased one level for 5-FU, and the nadir AGC is < 1,000 or platelet nadir is < 50,000, then discontinue adjuvant cisplatin and 5-FU.

If patients were dose reduced in cycle 1 of adjuvant treatment, then that reduced dose is continued, assuming the subsequent nadirs are AGC ≥ 1,500 and platelets ≥ 75,000.

Note: Prophylactic growth factors for neutropenia are not allowed. In the setting of infection*, they may be incorporated based on the judgment of the investigator. Growth factors for anemia (e.g., darbepoetin, erythropoietin) may be incorporated based on the judgment of the investigator.

*Defined as CTCAE, v.3.0 ≥ Grade 3, "Infection (documented clinically or microbiologically) with Grade 3 or 4 neutrophils (ANC< 2.0 X10^9/L) – Select".

7.6.6 *Dose Modifications for NonHematologic Adverse Events During Concurrent & Adjuvant Chemotherapy* **(4/21/08)**

Note: Dosing of chemotherapy will only change if there is a ≥ 10% weight loss from baseline.

7.6.6.1 Neutropenic fever: Temperature of 38.50C with AGC <1000 is an expected potential complication of concurrent chemotherapy and radiotherapy or chemotherapy alone. If neutropenic fever is noted, the chemotherapy dose reduction will be determined by the nadir counts.

7.6.6.2 Gastrointestinal (GI) Adverse Events Secondary to 5-FU: For Grade 3 GI adverse events (other than nausea and vomiting), including stomatitis and diarrhea, delay the chemotherapy cycle until ≤ grade 1, decrease 5-FU to the –1 level (800 mg/m2) for the remaining cycles. For Grade 4 GI adverse events (other than nausea and vomiting), delay the chemotherapy cycle until ≤ grade 1, then give subsequent doses of 5-FU at the –2 level (600 mg/m^2) for the

remaining cycles. If ≥ grade 3 adverse events occur after dose reduction to the –2 level, discontinue 5-FU.

7.6.6.3 Angina: Patients without prior history of angina, who develop angina that appears to be temporarily related to the infusion of 5-FU, must have the drug infusion stopped and the 5-FU permanently discontinued.

7.6.6.4 Renal Adverse Events: Dose will be modified based on the serum creatinine and/or creatinine clearance immediately prior to each cisplatin dose. If the serum creatinine is ≤ 1.5, creatinine clearance is not necessary for treatment with full dose. If the serum creatinine is > 1.5, a creatinine clearance should be obtained by urine collection or nomogram calculation (valid only if serum creatinine is not changing rapidly) and the dose modified as indicated below:

Creatinine		Creatinine Cl ml/min	Dose Reduction of Cisplatin
≤ 1.5	or	≥ 50 cc/min	No change
> 1.5	and	40-50 cc/min	Decrease 1 level
> 1.5	and	< 40 cc/min	Hold drug*

*Cisplatin must not be administered until creatinine is ≤ 1.5 or creatinine clearance ≥ 60. If creatinine remains > 1.5 or creatinine clearance remains < 40, the patient should not receive additional cisplatin.

7.6.6.5 Neurologic Adverse Events:

Grade	Drug	Dose Adjustment
2	Cisplatin	Decrease 1 level
3	Cisplatin	Hold drug*

*Recovery: Peripheral neuropathy is the largest concern with cisplatin. For neurologic toxicity attributed to cisplatin, treatment with 5-FU will continue uninterrupted. However, 5-FU is infrequently associated with cerebellar ataxia, somnolence, and upper motor neuron signs, and should be discontinued if these occur.

7.6.6.6 Ototoxicity: Should patients develop clinical evidence of ototoxicity, further audiometric evaluation is required. A neurologic deficit should be distinguished from a conductive loss from fluid in the eustachian tube. Cisplatin should be held for grade 3 hearing loss that has primarily a neurological basis; for grade 2 hearing loss with primarily a neurological basis, decrease 1 level.

7.6.6.7 Dermatitis secondary to 5-FU: For generalized symptomatic macular, papular, or vesicular eruption (grade 3), hold 5-FU until recovery. Resume at dose level -1 (800 mg/m^2 For grade 4 adverse events, resume at dose level -2 (600 mg/m^2).

7.6.7 *Treatment Modifications/Delay Guidelines for Bevacizumab* **(4/21/08)**

The dosing date for bevacizumab may be delayed for up to 3 days for logistical reasons (e.g., holiday, patient scheduling conflict). **Note:** Dosing of chemotherapy will only change if there is a ≥ 10% weight loss from baseline.

7.6.7.1 Hypertension: Hypertension is a known and potentially serious adverse event associated with bevacizumab treatment. Patients should have their BP monitored weekly during the first cycle of therapy and prior to each infusion of bevacizumab. Hypertensive mediation should be initiated or increased per routine practice.

7.6.7.2 Wound complications and surgery: The appropriate interval from discontinuation of bevacizumab to subsequent elective surgery required to reduce the risk of impaired wound healing has not been determined. Decision on such an interval should take into consideration the half-life of bevacizumab. It is generally recommended that bevacizumab should be discontinued at least 4 weeks prior to major elective surgery. In addition, bevacizumab should not be restarted until at least 4 weeks after major surgery provided that the wound has adequately healed.

7.6.7.3 Nutritional evaluation for a prophylactic gastrostomy (PEG) tube placement is STRONGLY ENCOURAGED. Prophylactic placement must occur ≥ 1 week prior to treatment. For patients who do not have a PEG tube placed prophylactically and who subsequently require placement during treatment, bevacizumab must be stopped at least 1 week prior and for 1 week after the placement of the PEG tube.

7.6.7.4 **There will be no dose reduction for bevacizumab. Treatment should be interrupted or discontinued for certain adverse events, as described below:**

Treatment Modification for Bevacizumab-Related Adverse Events (11/7/07)

CTCAE, v. 3.0 Term	CTCAE Grade/Definition	Action to be Taken
**Allergic reactions, or Acute infusional reactions/ cytokine release syndrome**	Grade 1: Transient flushing or rash; drug fever < 38 degrees C (< 100.4 degrees F)	If infusion-related or allergic reactions occur, premeds should be given with the next dose, and infusion time may be reduced for the subsequent infusion. Follow the guidelines in the Section 7.1.2.2 for bevacizumab administration.
	Grade 2: Rash; flushing; urticaria; dyspnea; drug fever ≥ 38 degrees C (≥ 100.4 degrees F)	If infusion-related or allergic reactions occur, premeds should be given with the next dose. Follow the guidelines in the Section 7.1.2.2 for bevacizumab administration.
	Grade 3: Symptomatic bronchospasm, with or without urticaria; parenteral medication(s) indicated; allergy-related edema/angioedema; hypotension	Bevacizumab infusion should be stopped and not restarted on the same day. At the physicians' discretion, bevacizumab may be permanently discontinued or re-instituted with premeds and at a rate of 90+15 min. **If bevacizumab is re-instituted, the patient should be closely monitored for a duration comparable to or longer than the duration of the previous reactions.**
	Grade 4: Anaphylaxis	Discontinue bevacizumab

CTCAE, v. 3.0 Term	CTCAE Grade/Definition	Action to be Taken
**ARTERIAL THROMBOSIS EVENTS:**		
**Cardiac ischemia/ infarction**	Grade 2: Asymptomatic and testing suggesting ischemia; stable angina	Discontinue bevacizumab.
	Grade 3: Symptomatic and testing consistent with ischemia unstable angina; intervention indicated	Discontinue Bevacizumab
	Grade 4: Acute myocardial infarction	Discontinue Bevacizumab
**CNS Cerebrovascular Ischemia (TIA, CVA) any peripheral or visceral arterial ischemia/thrombosis**	Grade 2: Asymptomatic; radiographic findings only (new or pre-existing)	Discontinue bevacizumab
	Grade 3: Transient ischemic event or attack (TIA) < 24 hours duration	Discontinue Bevacizumab
	Grade 4: Cerebral vascular accident (CVA, stroke), neurological deficit > 24 hours	Discontinue Bevacizumab
**Thrombosis/embolism**	Grade 2: Deep vein thrombosis or cardiac thrombosis; intervention (e.g., anticoagulation, lysis, filter, invasive procedure) not indicated	Omit 1 bevacizumab dose, and reassess the patient.
	Grade 3: Deep vein thrombosis or cardiac thrombosis; intervention (e.g., anticoagulation, lysis, filter, invasive procedure) indicated	▪ Hold bevacizumab treatment. If the planned duration of full-dose anticoagulation is <2weeks, bevacizumab should be held until the full-dose anticoagulation period is over.<br>▪ If the planned duration of full-dose anticoagulation is >2 weeks, bevacizumab may be resumed during the period of full-dose anticoagulation IF all of the criteria below are met:<br>- The subject must have an in-range INR (usually 2-3) on a stable dose of warfarin, or on stable dose of heparin prior to restarting bevacizumab.<br>- The subject must not have pathological conditions that carry high risk of bleeding (e.g. tumor involving major vessels or other conditions)<br>- The subject must not have had hemorrhagic events while on study<br>▪ If thromboemboli worsen/recur upon resumption of study therapy, discontinue bevacizumab
	Grade 4: Embolic event including pulmonary embolism or life-threatening thrombus	If symptomatic, discontinue bevacizumab. If thromboemboli worsen/recur upon resumption of study therapy, discontinue bevacizumab. If asymptomatic Grade 4, same as above.

CTCAE, v. 3.0 Term	CTCAE Grade/Definition	Action to be Taken
**Hypertension**<br>Treat with anti-hypertensive medication as needed. The goal of BP control should be consistent with general medical practice	Grade 1:<br>Asymptomatic, transient (< 24 hrs.) increase by >20 mmHg (diastolic) or to >150/100 if previously WNL; intervention not indicated Pediatric: Asymptomatic, transient (< 24 hrs.) BP increase >ULN; intervention not indicated	Consider increased BP monitoring
	Grade 2:<br>Recurrent or persistent (≥ 24 hrs.) or symptomatic increase by >20 mmHg (diastolic) or to >150/100 if previously WNL; monotherapy may be indicated Pediatric: Recurrent or persistent (≥ 24 hrs.) BP >ULN; monotherapy may be indicated	Begin anti-hypertensive therapy and continue bevacizumab
	Grade 3: Requiring more than one drug or more intensive therapy than previously	Bevacizumab should be held until symptoms have resolved AND BP Diastolic < 160/90mm Hg*<br><br>*If bevacizumab cannot be restarted after a four-week interruption, it should be discontinued.
	Grade 4: Life-threatening consequences (e.g., hypertensive crisis) Pediatric: Same as adult	Discontinue bevacizumab
**CNS Hemorrhage**	Grade 1: Asymptomatic, radiographic findings only (new or pre-existing)	Discontinue bevacizumab
	Grade 2: Medical intervention indicated	Discontinue bevacizumab
	Grade 3: Ventriculostomy, ICP monitoring, intraventricular thrombolysis, or operative intervention indicated	Discontinue bevacizumab
	Grade 4: Life-threatening consequences; neurologic deficit or disability	Discontinue bevacizumab
**Hemorrhage/ pulmonary/upper respiratory**	Grade 2: Symptomatic and medical intervention indicated	Discontinue bevacizumab
	Grade 3: Transfusion, interventional radiology, endoscopic, or operative intervention indicated; radiation therapy (i.e., hemostasis of bleeding site)	Discontinue bevacizumab
	Grade 4: Life-threatening consequences; major urgent intervention indicated	Discontinue bevacizumab

CTCAE, v. 3.0 Term	CTCAE Grade/Definition	Action to be Taken
**Hemorrhage/GI Bleeding**	Grade 3: Transfusion, interventional radiology, endoscopic, or operative intervention indicated; radiation therapy (i.e., hemostasis of bleeding site)	▪ Patients receiving full-dose anticoagulation should discontinue bevacizumab.<br>▪ For patients not on full-dose anticoagulation, hold bevacizumab until ALL of the following criteria are met:<br>- the bleeding has resolved and Hb is stable<br>- there is no bleeding diathesis that would increase the risk of therapy<br>- there is no anatomic or pathologic condition that could increase the risk of hemorrhage recurrence.<br>▪ Patients who experience recurrence of grade 3 hemorrhage should discontinue study therapy.
	Grade 4: Life-threatening consequences; major urgent intervention indicated	Discontinue bevacizumab
**GI Perforation**	Grade 2:Medical intervention indicated; IV fluids indicated < 24 hrs.	Discontinue bevacizumab
	Grade 3:IV fluids, tube feedings, or TPN indicated ≥ 24 hrs.; operative intervention indicated	Discontinue bevacizumab
**GI Leak** (including anastomotic) GI - Select is to be used for clinical signs/symptoms or radiographic confirmation of anastomotic or conduit leak (e.g., biliary, esophageal, intestinal, pancreatic, pharyngeal, rectal), but without development of fistula.	Grade 2: Symptomatic; medical intervention indicated	Discontinue bevacizumab
	Grade 3: Symptomatic and interfering with GI function; invasive or endoscopic intervention indicated	Discontinue bevacizumab
**GI Fistula** A fistula is defined as an abnormal communication between two body cavities, potential spaces, and/or the skin. The site indicated for a fistula should be the site from which the abnormal process is believed to have originated. For example, **a tracheo-esophageal fistula** arising in the context of a resected or irradiated esophageal cancer is graded as Fistula, GI – esophagus	Grade 2: Symptomatic; altered GI function (e.g., altered dietary habits, diarrhea, or GI fluid loss); IV fluids indicated < 24 hrs.	Discontinue bevacizumab
	Grade 3: Symptomatic and severely altered GI function (e.g., altered dietary habits, diarrhea, or GI fluid loss); IV fluids, tube feedings, or TPN indicated ≥ 24 hrs.	Discontinue bevacizumab
	Grade 4: Life-threatening consequences	Discontinue bevacizumab

For other fistulas, see table below.		
**Other clinically significant AEs attributable to bevacizumab** (except controlled nausea/vomiting)	Grade 3	▪ Hold bevacizumab until symptoms resolve to < grade 1<br>▪ If treatment delay is >3-4 weeks due to toxicity, discontinue bevacizumab.
	Grade 4	▪ Discontinue bevacizumab<br>▪ **Upon consultation with the study chair**, resumption of bevacizumab may be considered if a patient is benefiting from therapy, and the G4 toxicity is transient, has recovered to < grade 1 and unlikely to recur with retreatment.

**Other Bevacizumab Treatment Modifications Non-specific to CTCAE, v. 3.0 (5/7/07)**		
**Any Fistula, specifically in the head and neck region**	Any fistula	Discontinue bevacizumab at any time of the study therapy, concurrent or adjuvant phase.
**Hemorrhage within the radiation field**	≥ Grade 2	Discontinue bevacizumab
**During Concurrent Phase: Local adverse events (e.g., stomatitis, ulceration, dysphagia) that necessitate interruption of RT**	Grade 3-4	Hold bevacizumab until patient is able to resume RT
**During Adjuvant Phase: Local adverse events (e.g., stomatitis, ulceration, dysphagia)**	Grade 3-4	Hold bevacizumab until recovery to ≤ Grade 1.<br>Bevacizumab should be discontinued if local adverse events worsen or recur.
**Proteinuria**<br>Proteinuria should be monitored by urine analysis for urine protein creatinine (UPC) ratio prior to every dose of bevacizumab.	UPC ratio ≤ 3.5	Continue bevacizumab
	UPC ratio > 3.5	Hold bevacizumab until it UPC recovers to ≤ 3.5. If therapy is held for > 2 months due to proteinuria, discontinue bevacizumab.
	Nephrotic syndrome	Discontinue bevacizumab.
**Wound dehiscence requiring medical or surgical intervention**		Discontinue bevacizumab
**Reversible Posterior Leukoencephalopathy syndrome (RPLS)**		BV should be held in patients with symptoms/signs suggestive of RPLS, pending work-up and management, including control of blood pressure. BV should be discontinued upon diagnosis of RPLS.

7.7 Modality Review

The Medical Oncology Co-Chair, David Pfister, MD, will perform a Chemotherapy Assurance Review of all patients who receive or are to receive chemotherapy in this trial. In addition, an independent medical oncologist not associated with this study will review AdEERs reports of all hemorrhages (any grade or attribution) and all Grade 5 adverse events. The goal of the review is to evaluate protocol compliance. The review process is contingent on timely submission of chemotherapy treatment data as specified in Section 12.1. The scoring mechanism is: **per**

protocol; **not per protocol**; or **not evaluable for chemotherapy review.** A report is sent to each institution once per year to notify the institution about compliance for each case reviewed in that year.

The Medical Oncology Co-Chair, David Pfister, MD, will perform a Quality Assurance Review after complete data for the first 16 cases enrolled has been received at RTOG Headquarters. Dr. Pfister will perform the next review after complete data for the next 16 cases enrolled has been received at RTOG Headquarters. The final cases will be reviewed within 3 months after this study has reached the target accrual or as soon as complete data for all cases enrolled has been received at RTOG Headquarters, whichever occurs first.

7.8 Adverse Events (2/16/11)

As of April 1, 2011, this study will utilize the Common Terminology Criteria for Adverse Events (CTCAE) version 4, MedDRA, version 12.0 for grading of all adverse events reported via AdEERS. All RTOG case report forms will continue to use CTCAE, v. 3.0. A copy of the CTCAE v. 4 can be downloaded from the CTEP home page (http://ctep.cancer.gov) or the RTOG web site (http://www.rtog.org/members/toxicity/main.html). All appropriate treatment areas should have access to a copy of the CTCAE v. 4..

All adverse events (AEs) as defined in the tables below will be reported via the AdEERS (Adverse Event Expedited Reporting System) application accessed via the CTEP web site (https://webapps.ctep.nci.nih.gov/openapps/plsql/gadeers_main$.startup).

Serious adverse events (SAEs) as defined in Section 7.9 table below will be reported via AdEERS. Sites also can access the RTOG web site (http://www.rtog.org/members/toxicity/main.html) for this information.

In order to ensure consistent data capture, serious adverse events reported on AdEERS reports also must be reported on an RTOG case report form (CRF). In addition, sites must submit CRFs in a timely manner after AdEERS submissions.

In the rare instance when Internet connectivity is lost, an AE report may be submitted using CTEP's Adverse Event Expedited Report-Single Agent or Multiple Agent paper template (available at http://ctep.cancer.gov) and faxed to 301-230-0159. A 24-hour notification is to be made by CTEP by telephone at 301-897-7497, only when Internet connectivity is disrupted. Once Internet connectivity is restored, an AE report submitted on a paper template or a 24-hour notification made by phone must be entered electronically into AdEERS by the original submitter at the site.

7.8.1 Adverse Events (AEs)

Definition of an AE: Any unfavorable and unintended sign (including an abnormal laboratory finding), symptom, or disease temporally associated with the use of a medical treatment or procedure regardless of whether it is considered related to the medical treatment or procedure (attribution of unrelated, unlikely, possible, probable, or definite). [*CTEP, NCI Guidelines: Expedited Adverse Event Reporting Requirements*. December 2004.]

The following guidelines for reporting adverse events (AEs) apply to **all** NCI/RTOG research protocols. AEs, as defined above, experienced by patients accrued to this protocol should be reported on the AE section of the appropriate case report form (see Section 12.1). **Note: AEs indicated in the AdEERS Expedited Reporting Requirements in the Section 7.9 text and/or table also must be reported via AdEERS.**

NOTE: If the event is a Serious Adverse Event (SAE) [see next section], further reporting will be required. Reporting AEs only fulfills Data Management reporting requirements.

7.8.2 Serious Adverse Events (SAEs) — All SAEs that fit any one of the criteria in the SAE definition below must be reported via AdEERS. Contact the AdEERS Help Desk if assistance is required.

Certain SAEs as outlined below will require the use of the 24 Hour AdEERS Notification:

- **Phase II & III Studies: All unexpected potentially related SAEs**
- **Phase I Studies: All unexpected hospitalizations and all grade 4 and 5 SAEs regardless of relationship**

Definition of an SAE: Any adverse experience occurring during any part of protocol treatment and 30 days after that results in any of the following outcomes:

- Death;
- A life-threatening adverse drug experience;
- Inpatient hospitalization or prolongation of existing hospitalization;
- A persistent or significant disability/incapacity;
- A congenital anomaly/birth defect.

Important medical events that may not result in death, be life threatening, or require hospitalization may be considered an SAE drug experience, when, based upon medical judgment, they may jeopardize the patient and may require medical or surgical intervention to prevent one of the outcomes listed in the definition.

Pharmaceutically supported studies will require additional reporting over and above which is required by CTEP.

SAEs (more than 30 days after last treatment) attributed to the protocol treatment (possible, probable or definite) should be reported via AdEERS.

All supporting source documentation indicated as being provided in the Additional Information Section of the AdEERS Report must be properly labeled with the study/case numbers and the date of the adverse event and must be faxed to both the NCI at 301-230-0159 and the RTOG dedicated SAE FAX, 215-717-0990, before the five or ten-calendar-day deadline to allow RTOG to comply with the reporting requirements of the pharmaceutical company/companies supporting the RTOG trial. The RTOG Case Number without any leading zeros should be used as the Patient ID when reporting via AdEERS. Non-RTOG intergroup study and case numbers must also be included, when applicable. Submitted AdEERS Reports are forwarded to RTOG electronically via the AdEERS system.

SAE reporting is safety related and separate and in addition to the Data Management reporting requirements as outlined in the previous AE reporting section. **Any event that meets the above outlined criteria for an SAE but is assessed by the AdEERS System as "expedited reporting NOT required" must still be reported for safety reasons and to fulfill the obligations of RTOG to the pharmaceutical company/companies supporting the RTOG trial. Sites must bypass the "NOT Required" assessment and complete and submit the report. The AdEERS System allows submission of all reports regardless of the results of the assessment. Note:** Sites must select the option in AdEERS to send a copy of the report to the FDA or print the AdEERS report and fax it to the FDA, FAX 1-800-332-0178.

7.8.3 <u>Acute myeloid leukemia (AML) or myelodysplastic syndrome (MDS)</u> [2/16/11]

AML or MDS that is diagnosed during or subsequent to treatment in patients on NCI/CTEP-sponsored clinical trials must be reported via the AdEERS system **within 30 days of AML/MDS diagnosis.** If the site is reporting in CTCAE, v. 4, the event(s) maybe reported as 1) Leukemia secondary to oncology chemotherapy; 2) Myelodysplastic syndrome; or 3) Treatment-related secondary malignancy.

7.9 AdEERS Expedited Reporting Requirements

CTEP defines routine AE reporting requirements for phase 2 and 3 trials as described in the table below. **Important**: All AEs reported via AdEERS also must be reported on the AE section of the appropriate case report form (see Section 12.1).

7.9.1 Phase 2 and 3 Trials Utilizing an Agent under a CTEP IND: AdEERS Expedited Reporting Requirements for Adverse Events that Occur within 30 Days[1] of the Last Dose of the Investigational Agent, Bevacizumab, in this Study

	Grade 1	Grade 2	Grade 2	Grade 3		Grade 3		Grades 4 & 5[2]	Grades 4 & 5[2]
	Unexpected and Expected	Unexpected	Expected	Unexpected		Expected		Unex-pected	Expected
				with Hospitali-zation	without Hospitali-zation	with Hospitali-zation	without Hospitali-zation		

**Unrelated Unlikely**	Not Required	Not Required	Not Required	10 Calendar Days	Not Required	10 Calendar Days	Not Required	10 Calendar Days	10 Calendar Days
**Possible Probable Definite**	Not Required	10 Calendar Days	Not Required	10 Calendar Days	10 Calendar Days	10 Calendar Days	Not Required	24-Hour; 5 Calendar Days	10 Calendar Days

[1] **Adverse events with attribution of possible, probable, or definite that occur greater than 30 days after the last dose of treatment with an agent under a CTEP IND require reporting as follows:**

AdEERS 24-hour notification followed by complete report within 5 calendar days for:

- Grade 4 and Grade 5 unexpected events

AdEERS 10 calendar day report:

- Grade 3 unexpected events with hospitalization or prolongation of hospitalization
- Grade 5 expected events

[2] Although an AdEERS 24-hour notification is not required for death clearly related to progressive disease, a full report is required as outlined in the table.

Please see exceptions below under section entitled "Additional Instructions or Exceptions."

March 2005

Note: All deaths on study require both routine and expedited reporting regardless of causality. Attribution to treatment or other cause must be provided. "On study" is defined as during or within 30 days of completing protocol treatment.

- Expedited AE reporting timelines defined:
  - "24 hours; 5 calendar days" – The investigator must initially report the AE via AdEERS within 24 hours of learning of the event followed by a complete AdEERS report within 5 calendar days of the initial 24-hour report.
  - "10 calendar days" - A complete AdEERS report on the AE must be submitted within 10 calendar days of the investigator learning of the event.
- **(2/16/11)** Any medical event equivalent to CTCAE, v. 4 grade 3, 4, or 5 that precipitates hospitalization (or prolongation of existing hospitalization) must be reported regardless of attribution and designation as expected or unexpected with the exception of any events identified as protocol-specific expedited adverse event reporting exclusions.
- Any event that results in persistent or significant disabilities/incapacities, congenital anomalies, or birth defects must be reported via AdEERS if the event occurs following treatment with an agent under a CTEP IND.
- Use the NCI protocol number and the protocol-specific patient ID assigned during trial registration on all reports.

Additional Instructions or Exceptions to AdEERS Expedited Reporting Requirements for Phase 2 and 3 Trials Utilizing an Agent under a CTEP-IND:

- Hemorrhage, regardless of grade, requirement for hospitalization, or attribution, should be reported through AdEERS. Note: The AdEERS report must include the following: Source/site of bleeding; severity, duration, and quantity of bleeding; the patient's platelet, PT/INR, and PTT levels.
- Grade 3 fistula, regardless of requirement for hospitalization, should be reported through AdEERS;
- Grade 3-4 myelosuppression, whether or not hospitalization is required, does **not** require expedited reporting through AdEERS.

7.10 Clinical Trials Agreement

The Bevacizumab supplied by CTEP, DCTD, NCI used in this protocol is provided to the NCI under a CRADA between Genentech, Inc. (hereinafter referred to as "Collaborator") and the NCI Division of Cancer Treatment and Diagnosis. Therefore, the following obligations/guidelines, in addition to the provisions in the "Intellectual Property Option to Collaborator" (http://ctep.cancer.gov/industry/ipo.html) contained within the terms of award, apply to the use of the Agent(s) in this study:

1. Agent(s) may not be used for any purpose outside the scope of this protocol, nor can Agent(s) be transferred or licensed to any party not participating in the clinical study. Collaborator(s) data for Agent(s) are confidential and proprietary to Collaborator(s) and shall be maintained as such by the investigators. The protocol documents for studies utilizing investigational Agents contain confidential information and should not be shared or

distributed without the permission of the NCI. If a copy of this protocol is requested by a patient or patient's family member participating on the study, the individual should sign a confidentiality agreement. A suitable model agreement can be downloaded from: http://ctep.cancer.gov.

2. For a clinical protocol where there is an investigational Agent used in combination with (an)other investigational Agent(s), each the subject of different collaborative agreements , the access to and use of data by each Collaborator shall be as follows (data pertaining to such combination use shall hereinafter be referred to as "Multi-Party Data".):

   a. NCI will provide all Collaborators with prior written notice regarding the existence and nature of any agreements governing their collaboration with NIH, the design of the proposed combination protocol, and the existence of any obligations that would tend to restrict NCI's participation in the proposed combination protocol.
   b. Each Collaborator shall agree to permit use of the Multi-Party Data from the clinical trial by any other Collaborator solely to the extent necessary to allow said other Collaborator to develop, obtain regulatory approval or commercialize its own investigational Agent.
   c. Any Collaborator having the right to use the Multi-Party Data from these trials must agree in writing prior to the commencement of the trials that it will use the Multi-Party Data solely for development, regulatory approval, and commercialization of its own investigational Agent.

3. Clinical Trial Data and Results and Raw Data developed under a Collaborative Agreement will be made available exclusively to Collaborator(s), the NCI, and the FDA, as appropriate and unless additional disclosure is required by law or court order. Additionally, all Clinical Data and Results and Raw Data will be collected , used and disclosed consistent with all applicable federal statutes and regulations for the protection of human subjects, including, if applicable, the Standards for Privacy of Individually Identifiable Health Information set forth in 45 C.F.R. Part 164.
4. When a Collaborator wishes to initiate a data request, the request should first be sent to the NCI, who will then notify the appropriate investigators (Group Chair for Cooperative Group studies, or PI for other studies) of Collaborator's wish to contact them.
5. Any data provided to Collaborator(s) for Phase 3 studies must be in accordance with the guidelines and policies of the responsible Data Monitoring Committee (DMC), if there is a DMC for this clinical trial.
6. Any manuscripts reporting the results of this clinical trial must be provided to CTEP by the Group office for Cooperative Group studies or by the principal investigator for non-Cooperative Group studies for immediate delivery to Collaborator(s) for advisory review and comment prior to submission for publication. Collaborator(s) will have 30 days from the date of receipt for review. Collaborator shall have the right to request that publication be delayed for up to an additional 30 days in order to ensure that Collaborator's confidential and proprietary data, in addition to Collaborator(s)'s intellectual property rights, are protected. Copies of abstracts must be provided to CTEP for forwarding to Collaborator(s) for courtesy review as soon as possible and preferably at least three (3) days prior to submission, but in any case, prior to presentation at the meeting or publication in the proceedings. Press releases and other media presentations must also be forwarded to CTEP prior to release. Copies of any manuscript, abstract and/or press release/ media presentation should be sent to: Regulatory Affairs Branch, CTEP, DCTD, NCI
   Executive Plaza North, Suite 7111
   Bethesda, Maryland 20892
   FAX 301-402-1584
   Email: anshers@mail.nih.gov

The Regulatory Affairs Branch will then distribute them to Collaborator(s). No publication, manuscript or other form of public disclosure shall contain any of Collaborator(s) confidential/ proprietary information.

8.0 SURGERY

8.1 Neck Dissection

A neck dissection should be considered if a palpable or worrisome radiographic abnormality persists in the neck eight weeks after radiotherapy ends. In this rare occasion, chemotherapy and bevacizumab should be withheld for one month (28 days) after completion of neck dissection.

8.2 Cervical Lymphadenectomy

The type of neck dissection will depend on the extent of lymphadenopathy, and preservation of the accessory nerve, jugular vein, and sternomastoid muscle will be at the discretion of the surgeon.

8.3 Operative Report

The operative report must accurately and completely describe the precise location and the extent of the cervical lymph node metastases. Assessment of the completeness of the resection and results of intra-operative frozen section should be included.

9.0 OTHER THERAPY

9.1 Permitted Supportive Therapy

9.1.2 All supportive therapy for optimal medical care will be given during the study period at the discretion of the attending physician(s) within the parameters of the protocol and documented on each site's source documents as concomitant medication.

9.2 Non-permitted Supportive Therapy

9.2.1 Prophylactic use of amifostine or pilocarpine is not allowed.

9.2.2 Treatment with dipyramidole (Persantine®), ticlopidine (Ticlid®), clopidogrel (Plavix®), or cilostazol (Pletal®) is not allowed.

9.2.3 Growth factors for neutropenia (e.g., granulocyte colony stimulating factor), or anemia (e.g., darbepoetin, erythropoietin) during chemotherapy/bevacizumab concurrent with radiation. During adjuvant cisplatin/5-FU/bevacizumab, prophylactic growth factors for neutropenia are not allowed. (In the setting of infection, they may be incorporated based on the judgment of the investigator. Growth factors for anemia [e.g., darbepoetin, erythropoietin] may be incorporated based on the judgment of the investigator).

10.0 TISSUE/SPECIMEN SUBMISSION (4/21/08)

For patients on the study who have consented to submit specimens (see Appendix I).

The RTOG Biospecimen Resource at the University of California San Francisco acquires and maintains high quality specimens from RTOG trials. Tissue from each block is preserved through careful block storage and processing. The RTOG encourages participants in protocol studies to consent to the banking of their tissue. The RTOG Biospecimen Resource provides tissue specimens to investigators for planned and future translational research studies. Translational research studies integrate the newest research findings into current protocols to investigate important biologic questions.

In this study, tissue and blood will be submitted to the RTOG Biospecimen Resource for the purpose of banking for future translational research. Submission of specimens is highly recommended.

NOTE: **(4/21/08)** Specimens should not be submitted via RTOG 0514, the Head and Neck Cancer Tissue/Specimen Repository. Sites should follow the instructions below.

10.1 Specimen Collection for Tissue Banking and Translational Research

The following must be provided in order for the case to be evaluable for the Biospecimen Resource:

10.1.1 One H&E stained slide

10.1.2 A paraffin-embedded tissue block of the tumor or a 2 mm diameter core of tissue, punched from the tissue block containing the tumor with a skin punch and submitted in a plastic tube labeled with the surgical pathology number. **Note**: A kit with the punch, tube, and instructions can be obtained free of charge from the Biospecimen Resource (see Appendix VI). Block or core must be clearly labeled with the pathology identification number that corresponds to the Pathology Report.

10.1.3 A Pathology Report documenting that the submitted block or core contains tumor. The report must include the RTOG protocol number and patient's case number. The patient's name and/or other identifying information should be removed from the report. The surgical pathology numbers and information must not be removed from the report.

10.1.4 A Specimen Transmittal Form clearly stating that tissue is being submitted for the RTOG Biospecimen Resource; if for translational research, this should be stated on the form. The form must include the RTOG protocol number and patient's case number.

10.2 Serum/Plasma Collection (5/7/07)

10.2.1 The RTOG Head and Neck Translational Program has an excellent tract record for conducting correlative biomarker studies. As discussed in Section 1.0, relevant to this protocol are primary tumor VEGF and VEGFR-2 expression and circulating EBV DNA.

10.2.2 *Plan and Hypotheses*
Serum and plasma will be banked to conduct preliminary analysis of the distribution of VEGF and VEGFR-2 expression and plasma EBV DNA titer by racial populations (Asian versus Caucasian and others).

10.2.3 In this study, 6 mL of blood (3 mL serum and 3 mL EDTA plasma) will be drawn at baseline, within 2 weeks after the completion of adjuvant chemotherapy and bevacizumab and at 1 year after treatment (a total of 18 mL from each patient). Study collection kits are available free of charge from the RTOG Biospecimen Resource. See Appendix VII for a description of the kit, detailed collection instructions, and contact information.

10.2.4 *Serum and Plasma Collection Instructions* **(5/7/07)**

10.2.4.1 Serum

- After allowing the serum to clot, keep serum tubes at 4° C until processing (tubes may be on ice up to 2 hrs.). Centrifuge specimens at 1000 x g (approximately 2500 RPM for standard clinical centrifuge) at 4° C for 10 minutes.
- Using sterile techniques to avoid contamination, aliquot serum into cryovials and freeze. Take great care to collect only serum and avoid collecting any solid particulate matter before transferring serum into the cryovials.

10.2.4.2 Plasma **(10/22/09)**

- Collect 3 mL venous blood into an EDTA tube, and keep at 4° C until processing (tubes may be on ice up to 2 hrs.). Centrifuge specimens at 1, 600 g for 10 minutes.
- Carefully pipette out the supernatant without disturbing the blood cells, and put the plasma into a 1.5 mL eppendorf tube.
- Centrifuge at 16,000 g for 10 minutes to remove any residual cells.
- Pipette out the cell-free plasma, and put it into 1 ml cryovials.

10.2.4.3 Label each aliquot with study protocol and case number, the date and time of collection, specimen type (i.e., serum) and the time point at which the specimen was taken.

10.2.4.4 Specimens should be sent with a Specimen Transmittal Form documenting the date of collection of the serum; the RTOG protocol number, the patient's case number, and method of storage, for example, stored at -20° C, must be included. Questions regarding blood collection or shipment should be directed to the RTOG Biospecimen Resource (see contact information below). Ship by express overnight service, Monday through Thursday; avoid a weekend or holiday arrival date, and DO NOT ship on Friday.

10.3 Specimen Collection Summary (5/7/07, 10/22/09)

**Specimens for Tissue Banking and Translational Research**		
**Collected Pre-Treatment**		
Specimens taken from patient:	Submitted as:	Shipped:
One H & E stained slide of the primary tumor	H & E stained slide	Slide shipped ambient
A paraffin-embedded tissue block of the primary tumor taken before initiation of treatment or a 2 mm diameter core of tissue, punched from the tissue block with a skin punch	Paraffin-embedded tissue block or punch biopsy	Block or punch shipped ambient

**Collected pre-treatment (baseline); 2 weeks post-adjuvant chemo and bevacizumab; and 1 year post-treatment**		
Specimens taken from patient:	Submitted as	Shipped:
3 mL of whole blood in red-	Frozen serum samples	Serum sent frozen on dry ice

top tube and centrifuged for serum	containing a minimum of 0.05 mL per aliquot in 1 mL cryovials	via overnight carrier.
3 mL of whole blood in EDTA tube and centrifuged for plasma	Frozen plasma in 1 ml cryovials	Plasma sent frozen on dry ice via overnight carrier.

Submit materials for Tissue Banking and Translational Research as follows:

Mailing Address: For Non-frozen Specimens Only
RTOG Biospecimen Resource
University of California San Francisco
Campus Box 1800
1657 Scott Street, Room 223
San Francisco, CA 94143-1800

Courier Address (FedEx, DHL, etc.): For Frozen Specimens
RTOG Biospecimen Resource
University of California San Francisco
1657 Scott Street, Room 223
San Francisco, CA 94115

Questions: 415-476-RTOG (7864)/FAX 415-476-5271; RTOG@ucsf.edu

10.4 Reimbursement (4/21/08)
RTOG will reimburse submitting institutions $300 per case for fresh or flash frozen tissue; $200 per case for a block or core of material; $100 per case for 10-12 slides, $50 per case for urine, $300 per case for complex material (blood, serum, buffy coat cells). After confirmation from the RTOG Biospecimen Resource that appropriate materials have been received, RTOG Administration will prepare the proper paperwork and send a check to the institution. Pathology payment cycles are run twice a year in January and July and will appear on the institution's summary report with the institution's regular case reimbursement.

10.5 Confidentiality/Storage
(See the RTOG Patient Tissue Consent Frequently Asked Questions, http://www.rtog.org/tissuebank/tissuefaq.html for further details.)

10.5.1 Upon receipt, the specimen is labeled with the RTOG protocol number and the patient's case number only. The RTOG Biospecimen Resource database only includes the following information: the number of specimens received, the date the specimens were received, documentation of material sent to a qualified investigator, type of material sent, and the date the specimens were sent to the investigator. No clinical information is kept in the database.

10.5.2 Specimens for tissue banking will be stored for an indefinite period of time. Specimens for the translational research component of this protocol will be retained until the study is terminated, unless the patient has consented to storage for future studies. If at any time the patient withdraws consent to store and use specimens, the material will be returned to the institution that submitted it.

11.0 PATIENT ASSESSMENTS

11.1 Study Parameters: See Appendix II.

11.2 Evaluation During and Post-Treatment

11.2.1 *Chemotherapy and Biologic Therapy, Concurrent with Radiation Therapy*
History and physical, Zubrod performance status, weight, adverse event evaluation, CBC, differential and platelets, electrolytes (sodium, potassium, bicarbonate, chloride, BUN, glucose, calcium, phosphorus), serum creatinine, and Mg++ should be done weekly during concurrent therapy and then prior to the adjuvant phase. A urine protein: creatinine ratio (UPC) and a comprehensive metabolic panel (sodium, potassium, chloride, bicarbonate, glucose, blood urea nitrogen, creatinine, calcium, bilirubin, total protein, albumin, AST, ALT, and alkaline phosphatase) will be performed on each bevacizumab treatment date. [**Note**: Do not repeat the sodium, potassium, bicarbonate, chloride, BUN, glucose, and serum creatinine if it is being done in preparation for cisplatin on the same day].

11.2.2 *Adjuvant Chemotherapy and Biologic Therapy, After Radiation Therapy* **(4/21/08)**

- History and physical, and CBC, differential, and platelets should be done within 24 hours prior to each treatment.
- Zubrod performance status, weight, adverse event evaluation, sodium, potassium, bicarbonate, chloride, BUN, glucose, serum creatinine), and Mg++, should be done prior to each cycle of cisplatin and 5-FU.
- A CBC, differential, platelets, comprehensive metabolic panel (sodium, potassium, chloride, bicarbonate, glucose, blood urea nitrogen, creatinine, calcium, bilirubin, total protein, albumin, AST, ALT, and alkaline phosphatase), Mg++, and a urine protein: creatinine ratio (UPC) will be performed on each bevacizumab treatment date (no earlier than 24 hours prior to the treatment). The UPC is obtained for management of proteinuria. [**Note**: Do not repeat the sodium, potassium, bicarbonate, chloride, BUN, glucose, and serum creatinine if it is being done in preparation for cisplatin and 5FU on the same day.]
- A CBC also should be done to obtain nadir counts on days 77 (+/- 3 days), 98 (+/- 3 days), and 119 (+/- 3 days). Clinical assessment for interval toxicities is encouraged at this time as well.
- A dental evaluation should be done at approximately 3, 6, and 12 months following radiotherapy.
- An audiogram should be done following radiotherapy if the middle/inner ear is irradiated > 40 Gy and/or if any hearing loss, vertigo, or tinnitus occurs.

11.2.3 *Follow up* **(5/7/07)**

Follow up will be done at completion of concurrent treatment; at completion of adjuvant treatment; at 6, 9, and 12 months from start of treatment; every 3 months in year 2; every 6 months in years 3- 5; then annually.

- A chest x-ray and CBC, platelets, and TSH evaluation should be done every 6 months during the first 3 years.
- An MRI of the nasopharynx should be done at 2 months and 4 months after radiotherapy, every 6 months for 2 years, then annually
- An audiogram should be done annually if the middle/inner ear is irradiated > 40 Gy and/or if any hearing loss, vertigo, or tinnitus occurs.
- Biopsy: Any suspicious mucosal lesion in the upper aerodigestive tract; pharyngeal pain referred to the ear; any firm node that persists longer than four weeks; epistaxis; chronic nasal congestion not thought to be due to radiation mucosal changes.
- Post-operative follow up (for those patients who have surgery): Sites will document incidents of wound complications (dehiscence, hemorrhage, infection, etc.) on the Follow-up Form **(F1)**.

11.3 Measurement of Response

11.3.1 *Local or Regional Relapse*

Relapse is defined as reappearance of tumor after complete response. If possible, relapse should be confirmed by biopsy.

11.3.2 *Local or Regional Progression*

Progression is defined as an estimated increase in the size of the tumor (product of the perpendicular diameters of the two largest dimensions) of greater than 25%, taking as reference the smallest value of all previous measurements or appearance of new areas of malignant disease.

11.3.3 *Distant Metastasis*

Clear evidence of distant metastases (lung, bone, brain, etc.); biopsy is recommended where possible. A solitary, spiculated lung mass/nodule is considered a second primary neoplasm unless proven otherwise in a patient with a smoking history.

11.3.4 *Second Primary Neoplasm*

Tumor reappearing within the initial and immediate adjoining anatomical region of the primary will be considered local recurrence. Multiple lung nodules/masses are considered distant metastases from the index cancer unless proven otherwise.

11.4 Discontinuation of Protocol Treatment

Protocol treatment may be discontinued for any of the following reasons:

- Progression of disease;
- Sustained, severe debilitation resulting in chronic dehydration and/or progressive unintentional weight loss (> 25% of baseline weight) unresponsive to tube feeding;
- Unacceptable adverse events [at the discretion of the treating physician(s)];
- A delay in protocol treatment > 2 weeks.

If protocol treatment is discontinued, follow up and data collection will continue as specified in the protocol.

12.0 DATA COLLECTION

Data should be submitted to:

RTOG Headquarters*
1818 Market Street, Suite 1600
Philadelphia, PA 19103

***If a data form is available for web entry, it must be submitted electronically.**

Patients will be identified by initials only (first middle last); if there is no middle initial, a hyphen will be used (first-last). Last names with apostrophes will be identified by the first letter of the last name.

12.1 Summary of Data Submission (5/7/07)

Item	Due
Demographic Form **(A5)**<br>Initial Evaluation Form **(I1)**<br>Pathology Report **(P1)**<br>Slides/Blocks **(P2)**	Within 2 weeks of study entry
Follow-up Form **(F1)**	At completion of concurrent treatment; at completion of adjuvant treatment; at 6, 9, and 12 months from start of treatment; every 3 months in year 2; every 6 months in years 3-5; then annually; also at death
Surgical Evaluation **(S1)**<br>Surgical Operative Report **(S2)**<br>Surgical Pathology Report **(S5)**	For patients who have surgery: Within 4 weeks of surgery

12.2 Summary of Dosimetry Digital Data Submission (Submit to ITC; see Section 12.2.1) [11/7/07]

Item	Due
**Preliminary Dosimetry Information**	
†Digital Data Submission Form **(DDSI)**	Within 1 week of start of RT
CT data, critical normal structures, all GTV, CTV, and PTV contours **(C1, C3)**	
Simulation films and/or digital film images for all initial treatment fields and orthogonal set up pair	
First day port films (or digital images) of all initial treatment fields and orthogonal set up pair	
Digital beam geometry for initial and boost beam sets	
Doses for initial and boost sets of concurrent treated beams	
Digital DVH data for all required critical normal structures, GTV, CTV, and PTVs for total dose plan **(DV)**	
Hard copy isodose distributions for total dose plan as described in QA guidelines† **(T6)**	
**Final Dosimetry Information**	Within 1 week of RT end
Radiotherapy Form **(T1) [copy to HQ and ITC]**	
Daily Treatment Record **(T5) [copy to HQ and ITC]**	
Simulation films and/or digital film images (or digital images) of all boost treatment fields and orthogonal	

set up pair **(T8)**
First day port films of all boost treatment fields and orthogonal set up pair **(T8)**
Modified digital patient data as required through consultation with Image Guided Therapy QA Center

†Available on the ATC web site, http://atc.wustl.edu/

12.2.1 *Digital Data Submission to ITC* **(10/22/09)**
Digital data submission may be accomplished using media or the Internet.
For network submission: The FTP account assigned to the submitting institution by the ITC shall be used, and e-mail identifying the data set(s) being submitted shall be sent to:

itc@wustl.edu

For media submission: Please contact the ITC about acceptable media types and formats.
Hardcopies accompanying digital data should be sent by mail or Federal Express and should be addressed to:

Image-Guided Therapy Center (ITC)
ATTN: Roxana Haynes
4511 Forest Park, Suite 200
St. Louis, MO 63108
314-747-5415
FAX 314-747-5423

13.0 STATISTICAL Considerations

13.1 Study Endpoints (4/21/08)

13.1.1 *Primary Endpoint*
Grade 4 hemorrhage or any Grade 5 adverse event assessed to be definitely, probably, or possibly related to protocol treatment during the first year

13.1.2 *Secondary Endpoints*

13.1.2.1 Grade 4 hemorrhage or any Grade 5 adverse event assessed to be definitely, probably, or possibly related to protocol treatment after the first year;
13.1.2.2 Patient tolerability to each component (concurrent and adjuvant) of the protocol treatment regimen;
13.1.2.3 Other ≥ Grade 3 adverse events;
13.1.2.4 Death during or within 30 days of discontinuation of protocol treatment;
13.1.2.5 One- and two-year distant metastases-free rates;
13.1.2.6 One- and two-year local-regional progression-free rates;
13.1.2.7 One- and two-year progression-free survival rates;
13.1.2.8 One- and two-year overall survival rates.

13.2 Background and Sample Size Determination (5/7/07)

The baseline rates used in this study are based upon the recently completed phase II nasopharyngeal trial, RTOG 0225.

Since there is limited experience with the proposed protocol regimen, the study will determine whether it can be delivered per protocol prescription and is safe. There is particular concern with the incidence of patients with grade 4 hemorrhage with BV. Therefore, the incidence of patients with either grade 4 hemorrhage or any grade 5 adverse event attributed to the protocol treatment will be used to evaluate safety. The incidence of these events will be examined in the first year from the start of treatment and then beyond the first year. The primary endpoint will examine safety based only upon the first year because follow-up for this time period will be complete and the incidence is expected to be lower beyond the first year.

(4/21/08) Treatment efficacy also will be evaluated (see secondary endpoints, Sections 13.1.2.5-13.1.2.8). The metastasis, progression, and survival rates in this study will be compared with the historical rates from RTOG 0225. The addition of BV is hypothesized to reduce rate of distant metastases. Since the local-regional control rate for this population is already outstanding (~90%)

given the chemoradiotherapy regimen without BV, a further improvement of local-regional control is not anticipated.

The targeted sample size is based upon the incidence of patients with either grade 4 hemorrhage or any grade 5 adverse events assessed to be definitely, probably, or possibly related to protocol treatment during the first year. The unacceptable rate for such adverse events is set at ≥ 15% and the acceptable rate at ≤ 5%. Then the statistical hypothesis would be:

> H0: Incidence of patients with either grade 4 hemorrhage or any grade 5 adverse event for the protocol treatment regimen ≤ 0.05
>
> HA: Incidence of patients with either grade 4 hemorrhage or any grade 5 adverse event for the protocol treatment regimen ≥ 0.15

The following table gives the number of patients with the above specified adverse events that are considered unacceptable as calculated by the method of Fleming[82] for a two-stage design where the type I error and the statistical power were set at 0.14 and 0.83, respectively. The first stage will utilize one-third of the required sample size thus permitting an earlier formal evaluation of adverse events.

Number of patients with specified adverse events	Total number evaluable
3	14
4	42

Four patients will be entered on the study in addition to the 42 evaluable patients to guard against the possibility that some patients may not start protocol treatment, may be found retrospectively ineligible, or may have no baseline or follow-up data submitted. Two of these four patients will be entered for the first stage of the study, and the remaining two patients will be entered for the second stage. Therefore, the **total sample size required** for the study is **46 patients**.

At the time of final analysis, patient tolerability also will be evaluated in terms of protocol treatment delivery. The protocol treatment regimen will be considered as 2 treatment components (concurrent and adjuvant). Tolerability for the concurrent component will be measured by the percentage of patients who received 2 or more cycles of cisplatin (CDDP) and bevacizumab (BV) during concurrent treatment with RT and RT scored by the study chair as no variation or minor variation. Tolerability for the adjuvant component will be measured by the percentage of patients who received 2 or more cycles of CDDP and 5-FU and BV during the adjuvant treatment phase.

A tolerability rate of 75% will be considered the minimum acceptable rate for a treatment component, while a rate less than 50% will be considered unacceptably low. If the true tolerability rate is 75% or more, there is less than a 1% chance that the regimen will be identified as unacceptable assuming a binomial distribution with a one-sided test. If the true tolerability rate is 50% or less, there is less than a 1% chance that the regimen will be identified as acceptable assuming a binomial distribution with a one-sided test.

13.3 Patient Accrual

It is projected that there will be a period of approximately 6 months with very slow accrual at the beginning of this study to allow for both institutional IRB approval and IMRT approval by the RTOG QA center. After this initial period, it is projected that this study will accrue approximately 2.5 patients per month. **Patient accrual will be discontinued after 16 patients have been entered and until the first stage analysis shows that the frequency of patients with Grade 4 hemorrhage or Grade 5 adverse event as described in 13.2 is acceptable**. The suspension will be 3-6 months in duration, allowing time for patients to complete the concurrent component and for the data analysis to be performed. Total accrual time including the time for suspension is projected to be approximately 27-30 months.

Note: Institutions in Canada and the Far East are being recruited as new RTOG members and have expressed interest in participating in the follow-up phase III study; some of these sites will able to participate in this phase II study and this would increase the accrual rate.

13.4 Analysis Plan

13.4.1 *Statistical Methods*

- The rates of tolerability and adverse events will be estimated using a binomial distribution along with their associated 95% confidence intervals. Only adverse events assessed by the treating institution as definitely, probably, or possibly related to protocol treatment (or with missing/unknown relationship) will be considered in evaluating the primary endpoint. Rates of distant metastases-free interval and local-regional progression-free interval will be estimated using the cumulative incidence method[83] while progression-free and overall survival rates will be estimated using the Kaplan-Meier method.[84] Failure for each endpoint is defined as follows:Local-regional progression-free interval: local or regional progression or recurrence or death due to study cancer with undocumented site of failure (i.e., no local, regional, or distant progression) or from unknown causes with undocumented site of failure;
- Distant metastases-free interval: distant metastases;
- Progression-free survival: local or regional progression or recurrence, distant metastases, or death due to any cause;
- Overall survival: death due to any cause.

13.4.2 *Independent Review of Hemorrhages and Grade 5 Adverse Events*

All hemorrhages (any grade or attribution) and all Grade 5 adverse events will be reviewed by a medical oncologist not associated with this study. The grade and attribution for all events will be reported in two ways: as scored by the treating institution and as scored by the independent reviewer.

13.4.3 *Interim Analysis to Monitor Study Progress*

Interim reports will be prepared twice each year until the final analysis has been accepted for presentation or publication. In general, these reports will contain information about the accrual rate with projected completion date for the accrual phase, exclusion rates and reasons, pretreatment characteristics of patients accrued, compliance rate of treatment delivered with respect to the protocol prescription, and the frequency and severity of adverse events. This study will be monitored by the Clinical Data Update System (CDUS) version 3.0. Cumulative CDUS data will be submitted quarterly by electronic means. Reports are due January 31, April 30, July 31, and October 31.

13.4.4 *Special Analysis of Adverse Events* **(5/7/07)**

As described in Section 13.3, accrual will be suspended after 16 patients and the study will not be reopened to accrual until the data for the first evaluation after the concurrent phase have been received for all 16 patients and the rate of adverse events is determined to be acceptable. While the primary endpoint is the rate of Grade 4 hemorrhage and Grade 5 adverse events in the first year, for practical reasons the analysis to evaluate the rate of hemorrhage will only require that data for the concurrent phase have been received for all 16 patients. Many patients also will have data by this time of the adjuvant phase, and these data will be included. The suspension will be 3-6 months in duration, allowing time for patients to complete the concurrent component and for the data analysis to be performed. Every effort will be made to complete radiation therapy (RT) delivery review before this analysis is started. However, it will not be delayed because of it. Rates of hemorrhage by grade and attribution will be reported in two ways: as scored by the treating institution and as scored by the independent reviewer. Patients with Grade 4 hemorrhage or any Grade 5 adverse event as described in Section 13.2 will be identified. If there are 3 or more such patients out of the original 14 eligible patients by either institutional scoring or independent review, the study chairs will review the data pertaining to these events. After their review, the study chairs and study statistician will make a recommendation to the RTOG Head and Neck Steering Committee, RTOG Research Strategy Committee, CTEP, and the corporate sponsor for their consideration. These committees and individuals jointly will decide the future course of action for the study.

13.4.5 *Analysis for Reporting the Initial Treatment Results*

The analysis to report the initial results of treatment will be undertaken when each patient has been potentially followed for a minimum of 12 months. Only eligible patients with both on-study and follow-up information that start protocol treatment will be included. The emphasis of this analysis will be on treatment compliance and adverse events. The usual components of this analysis are:

- Tabulation of all cases entered, and any excluded from analysis with reasons for exclusion;
- Patient accrual rate;
- Institutional accrual;

- Distribution of important baseline prognostic variables;
- Frequency and severity of adverse events;
- Compliance rate for treatment delivery with respect to the protocol prescription;
- Observed results with respect to the endpoints described in Section 13.1.

The rates of adverse events and tolerability will be estimated along with their associated 95% confidence intervals. Rates of hemorrhage by grade and attribution will be reported in two ways: as scored by the treating institution and as scored by the independent reviewer. Patients with Grade 4 hemorrhage or any Grade 5 adverse event as described in Section 13.2 will be identified. If there are 4 or more such patients out of the first 42 eligible patients by either institutional scoring or independent review, the protocol treatment will be considered to have an unacceptably high adverse event rate to use this treatment without modification in the follow-up phase III trial. In addition, the tolerability rate for each treatment component (concurrent and adjuvant) will be computed. If the percentage is less than 50% for a component, then that component will be considered to have an unacceptably low tolerability rate to use without modification in the follow-up phase III trial, then the treatment component will be dropped or modified. Subgroup analyses will not be undertaken because of the relatively small sample sizes.

13.4.6 *Analysis for Reporting the Final Treatment Results* **(4/21/08)**

The analysis to report the final results of treatment will be undertaken when each patient has been potentially followed for a minimum of 2 years. Only eligible patients with both on-study and follow-up information that start protocol treatment will be included. The emphasis of this analysis will be on local-regional progression and distant metastases. The usual components of this analysis are:

- Tabulation of all cases entered, and any excluded from analysis with reasons for exclusion;
- Patient accrual rate;
- Institutional accrual;
- Distribution of important baseline prognostic variables;
- Frequency and severity of adverse events;
- Compliance rate for treatment delivery with respect to the protocol prescription;
- Observed results with respect to the endpoints described in Section 13.1.

The rates of local-regional progression-free interval, distant metastases-free interval, progression-free survival, and overall survival at one and two years will be estimated along with their associated 95% confidence intervals. These rates also will be compared with historical rates from RTOG 0225. Subgroup analyses will not be undertaken because of the relatively small sample sizes.

13.5 Inclusion of Women and Minorities

In conformance with the National Institutes of Health Revitalization Act of 1993 with regard to inclusion of women and minorities in clinical research, we have considered the possible interactions (treatment by race and treatment by gender). The study was designed under the assumption of the same results between the gender and among the races.

Based on the accrual to the completed protocol for nasopharyngeal cancer, RTOG 0225, we project that 75% of patients enrolled on this study will be male and 25% female; 50% white and 50% non-white; 10% Hispanic and 90% non-Hispanic. The following table gives the projected number of patients in each race, ethnicity, and gender group.

Projected Distribution of Gender and Minorities

	Gender		
**Ethnic Category**	**Females**	**Males**	**Total**
Hispanic or Latino	0	5	5
Not Hispanic or Latino	11	30	41
**Ethnic Category: Total of all subjects**	11	35	**46**
	**Gender**		
**Racial Category**	**Females**	**Males**	**Total**
American Indian or Alaskan Native	0	0	0
Asian	5	14	19
Black or African American	1	4	5
Native Hawaiian or other Pacific Islander	0	0	0
White	5	17	22
**Racial Category: Total of all subjects**	11	35	**46**

REFERENCES

1. Al-Sarraf M, LeBlanc M, Giri PG, et al. Chemoradiotherapy versus radiotherapy in patients with advanced nasopharyngeal cancer: Phase III randomized intergroup study 0099. *J Clin Oncol*. 16:1310-1317,1998.

2. Baujat B, Audry H, Bourhis J, et al. Chemotherapy in locally advanced nasopharyngeal carcinoma: An individual patient data meta-analysis of eight randomized trials and 1753 patients. *Int J Radiat Oncol Biol Phys*. 47-56, 2006.

3. Langendijk JA, Leemans CR, Buter J., et al. The additional value of chemotherapy to radiotherapy in locally advanced nasopharyngeal carcinoma: A meta-analysis of the published literature. *J Clin Oncol*. 4604-4612, 2004.

4. Leslie MD, Dische S. The early changes in salivary gland function during and after radiotherapy given for head and neck cancer. Radiother Oncol. 30:26-32, 1994.

5. Mira JG, Wescott WB, Starcke EN, Shannon IL. Some factors influencing salivary function when treating with radiotherapy. Int J Radiat Oncol Biol Phys. 7:535-541, 1981.

6. Harrison LB, Zelefsky MJ, Pfitzer,DG, et al. Detailed quality of life assessment in patients treated with primary radiotherapy for cancer of the base of tongue. Head and Neck. 19:169-175, 1997.

7. Balogh JM, Sutherland SE. Osteoradionecrosis of the mandible: A review. J Otolaryngol. 18(5):245-250, 1989.

8. Cooper JS, Fu K, Marks J, Silverman S. Late effects of radiation therapy in the head and neck region. Int J Radiat Oncol Biol Phys. 31:1141-1164, 1995.

9. Shannon IL, Trodahl JN, Wescott, WB. Effects of radiotherapy on whole saliva flow. J Dent Res. 56:693, 1977.

10. Chu A, Flynn MB, Achino E, et al. Irradiation of nasopharyngeal carcinoma: Correlations with treatment factor and stage. *Int J Radiat Oncol Biol Phys*. 10: 2241-2249, 1984.

11. Hoppe RT, Goffinet DR, Bagshaw, MA. Carcinoma of the nasopharynx: Eighteen years' experience with megavoltage radiation therapy. *Cancer*. 37:2605,1976.

12. Mesic JB, Fletcher GH, Goepfert H. Megavoltage irradaition of epithelial tumors of the nasopharynx. *Int J Radiat Oncol Biol Phys*. 7:447-453, 1981.

13. Sanguineti G, Geara F, Garden A, et al. Carcinoma of the nasopharynx treated by radiotherapy alone: Determinants of local and regional control. *Int J Radiat Oncol Biol Phys*. 37:983-996, 1997.

14. Bailet, JW, Mark RJ, Abemayor E, et al. Nasopharyngeal carcinoma: Treatment results with primary radiation therapy. *Laryngoscope*. 102:965-972, 1992.

15. Vikram B, Strong EW., Manolatos S, et al. Improved survival in carcinoma of the nasopharynx. *Head Neck Surg*. 7:123-128, 1984.

16. Wang CC. Carcinoma of the nasopharynx. In: Wang, CC, ed. *Radiation Therapy for Head and Neck Neoplasms: Indications, Techniques, and Results*. 2nd ed. Chicago, Ill: Year Book Medical Publishers, Inc; 1990: 261-83.

17. Teo PML, MA BBY, Chan ATC. Radiotherapy for nasopharyngeal carcinoma-transition from two dimensional to three-dimensional methods. Radioth Oncol 2004;73:163-172.

18. Liu WS, SU MC, WU MF, et al. Nasopharyngeal carcinoma treated with precision-oriented radiation therapy techniques including intensity-modulated radiotherapy : preliminary resutls. Kaohsiung J Med Sci 2004;20:49-55.
19. Lee AW, Yau TK, Wong DH, et al. Treatment of stage IV(A-B) nasopharyngeal carcinoma by induction-concurrent chemoradiotherapy and accelerated fractionation. Int J Radiat Oncol BIol Phys 2005;63:1331-8.

20. Sutanem K, Shu HK, Xia P, et al. Three-dimensional intensity-modulated radiotherapy in the treatment of nasopharyngeal carcinoma: the University of Califronia-San Francisco experience. Int J Radiat Oncol Biol PHys 2000;48:711-22.

21. Nutting C, Dearnaley DP, Webb S. Intensity modulated radiation therapy; a clinical review. Br J Radiol. 73:459-469, 2000.

22. Butler EB, The BS, Grant, WH, et al. Smart (simultaneous modulated accelerated radiation therapy) boost: a new accelerated fractionation schedule for the treatment of head and neck cancer with intensity modulated radiotherapy. Int J Radia Oncol Biol Phys. 45:21-32, 1999.

23. Eisbruch A, Ship JA, Martel MK, et al. Parotid gland sparing in patients undergoing bilateral head and neck irradiation; techniques and early results. Int J Radiat Oncol Biol Phys. 36:469-480, 1996.

24. Eisbruch A, Ten Haken RK, Kim HM, et al. Dose, volume, and function relationships in parotid salivary glands following conformal, and intensity-modulated irradiation of head and neck cancer. Int J Radiat Oncol Biol Phys. 45:577-587, 1999.

25. Xia P, Fu KK, Wong GW, et al. Comparison of treatment plans involving intensity modulated radiotherapy for nasopharyngeal carcinoma. Int J Radiat Oncol Biol Phys. 48;329-337, 2000.

26. Wolden SL, Zelefsky MJ., Hunt MA, et al. Failure of a 3d conformal boost to improve radiotherapy for nasopharyngeal carcinoma. Int J Radiat Oncol Biol Phys. 49:1229-1234, 2001.

27. Hunt MA, Zelefsky MJ, Wolden S, et al. Treatment planning and delivery of intensity-modulated radiation therapy for primary nasopharynx cancer. Int J Radiat Oncol Biol Phys. 49:623-632, 2001.

28. Lee N, Xia P, Quivey JM, et al. Intensity-modulated radiotherapy in the treatment of nasopharyngeal carcinoma : An update of the UCSF experience. *Int J Radiat Oncol Biol Phys*.12-22, 2002.29.

29. Kam MK, Leung SF, Zee B, et al. Impact of Intensity-modulated radiotherapy (IMRT) on salivary gland function in early-stage nasopharyngeal carcinoma (NPC) patients. *Proc Am Soc Clin Oncol*. 23: 500s, 2005.

30. Kam MK, Teo PM, Chau RM, et al. Treatment of nasopharyngeal carcinoma with intensity-modulated radiotherapy: The Hong Kong experience. *Int J Radiat Oncol Biol Phys*.1440-50, 2004.

31. Kwong DL, Pow EH, Sham JS, et al. Intensity-modulated radiotherapy for early-stage nasopharyngeal carcinoma: A prospective study on disease control and preservation of salivary function. *Cancer*. 584-93, 2004.

32. Wolden SL, Chen WC, Pfister DG, et al. Intensity-modulated radiation therapy (IMRT) for nasopharynx cancer: Update of the Memorial Sloan-Kettering experience. *Int J Radiat Oncol Biol Phys*.57-62, 2006.

33. Kyzas PA, Cunha IW, Ionnidis JPA. Prognostic significance of vascular endothelial growth factor immunohistochemical expression in head and neck squamous cell carcinoma: A meta-analysis. *Clin Cancer Res.* 11: 1434-1440, 2005.

34. Wakisaka N, Wen Q, Yoshizaki T, et al. Association of vascular endothelial growth factor expression with angiogenesis and lymph node metastasis in nasopharyngeal carcinoma. *Laryngoscope*. 810-814, 1999.

REFERENCES (Continued)

35. Qian CN, Zhang CQ, Guo X, et al. Elevation of serum vascular endothelial growth factor in male patients with metastatic nasopharyngeal carcinoma. *Cancer.* 255-61, 2000.

36. Krishna SM, James S, Balaram P. Expression of VEGF as prognosticator in primary nasopharyngeal cancer and its relation to EBV status. *Virus Res*. 85-90, 2006.

37. Druzgal CH, Chen Z, Yeah NT. A pilot study of longitudinal serum cytokine and angiogenesis factor levels as markers of therapeutic response and survival in patients with head and neck squamous cell carcinoma. *Head Neck*. 771-784, 2005

38. Ferrara N. Vascular endothelial growth factor: Basic science and clinical progress. *Endocr Rev*. 25:581-611, 2004.

39. Presta, L.G., H. Chen, S.J. O'Connor, et al. Humanization of an anti-vascular endothelial growth factor monoclonal antibody for the therapy of solid tumors and other disorders. *Cancer Res*. 57: 4593-4599, 1997.

40. Borgstrom P, Gold DP, Hillan KJ, Ferrara N. Importance of VEGF for breast cancer angiogenesis in vivo: Implications from intravital microscopy of combination treatments with an anti-VEGF neutralizing monoclonal antibody and doxorubicin. *Anticancer Res*. 19: 4203-4214, 1999.

41. Kabbinavar F, Wong JT, Ayala RE, et al. The effect of antibody to vascular endothelial growth factor and cisplatin on the growth of lung tumors in nude mice. *Proc Am Assoc Cancer Res*. 36: A488, 1995.

42. Kim KJ, Li B, Winer J, et al. Inhibition of vascular endothelial growth factor-induced angiogenesis suppresses tumour growth in vivo. *Nature*. 362: 841-844, 1993.

43. Bergers G, Song S, Meyer-Morse N, et al. Benefits of targeting both pericytes and endothelial cells in the tumor vasculature with kinase inhibitors. *J Clin Invest*. 111: 1287-1295, 2003.

44. Shaheen RM, Ahmad SA, Liu W, et al. Inhibited growth of colon cancer carcinomatosis by antibodies to vascular endothelial and epidermal growth factor receptors. *Br J Cancer*. 85: 584-589, 2001.

45. Avastin™ (bevacizumab) Investigator’s Brochure.

46. Cobleigh MA, Langmuir VK, Sledge GW, et al. A phase I/II dose-escalation trial of bevacizumab in previously treated metastatic breast cancer. *Semin Oncol.* 30:117-124, 2003.

47. Hurwitz HL, Fehrenbacher CT, et al. Bevacizumab (a monoclonal antibody to vascular endothelial growth factor) prolongs survival in first-line colorectal cancer (CRC): Results of a phase III trial of bevacizumab in combination with bolus IFL (irinotecan, 5-fluorouracil, leucovorin) as first-line therapy in subjects with metastatic CRC. *Proc Am Soc Clin Oncol*. 22: A3646, 2003.

48. Miller K, Rugo HS, Cobleigh MA, et al. Phase III trial of capecitabine (Xeloda®) plus bevacizumab (Avastin™) versus capecitabine alone in women with metastatic breast cancer (MBC) previously treated with an anthracycline and a taxane. *Breast Cancer Res Treat*. 76(S37), 2002.

49. Yang JC, Haworth L, Sherry RM, et al. A randomized trial of bevacizumab, an anti-vascular endothelial growth factor antibody, for metastatic renal cancer. *NEJM*. 349:427-434, 2003.

50. Jain RK. Normalization of tumor vasculature: An emerging concept in antiangiogenic therapy. *Science*. 307: 58-62, 2005.

51. Burger RA, Sill M, Monk BJ, et al. Phase II trial of bevacizumab in persistent or recurrent epithelial ovarian cancer or primary peritoneal cancer: A Gynecologic Oncology Group Study. *Proc Am Assoc Cancer Res*. A5009, 2005.

REFERENCES (Continued)

52. Hurwitz H, Fehrenbacher L, Novotny W, et al. Bevacizumab plus irinotecan, fluorouracil, and leucovorin for metastatic colorectal cancer. *NEJM*. 350: 2335-2342, 2004.

53. Sandler AB, Gray R, Brahmer J, et al. Randomized phase II/III trial of paclitaxel (P) plus carboplatin (C) with or without bevacizumab in patients with advanced non-small cell lung cancer (NSCLC): An Eastern Cooperative Group (ECOG) Trial-E4599. ASCO Annual Meeting. LBA 4, 2005

54. Miller KD, Wang M, Gralow J, et al. E2100-A randomized phase III trial of paclitaxel versus paclitaxel plus bevacizumab as first-line therapy for locally recurrent or metastatic breast cancer. ASCO Annual Meeting. Educational Session, 2005.

55. Johnson DH, Fehrenbacher L, Novotny WF, et al. Randomized phase II trial comparing bevacizumab plus carboplatin and paclitaxel with carboplatin and paclitaxel alone in previously untreated locally advanced or metastatic non-small-cell lung cancer. *J Clin Oncol.* 22: 2184-2191, 2004.

56. Teicher BA, Holden SA, Gulshan A, et al. Influence of an anti-angiogenic treatment on 9L gliosarcoma: Oxygenation and response to cytotoxic therapy. *Int J Cancer*. 61: 732-737, 1995.

57. Willet C. Surrogate markers for antiangiogenic therapy and dose-limiting toxicities for bevacizumab with radiation and chemotherapy: Continued experience of a phase I trial in rectal cancer patients. *J Clin Oncl.* 8136-8138 [correspondence], 2006.

58. Willett CG, Chung D, Sahani D, et al. Phase I study of neoadjuvant bevacizumab, 5-fluorouracil, and radiation therapy followed by surgery for patients with primary rectal cancer. ASCO Annual Meeting, A3589, 2004.

59. Novotny W, Holmgren E, Griffing S, et al. Identification of squamous cell histology and central, cavitary tumors as possible risk factors for pulmonary hemorrhage in patients with advanced NSCLC receiving bevacizumab. *Proc Am Soc Clin Oncol*. 20: A1318, 2001.

60. Schilling, et al. ASCO, 2005.

61. Vokes EE, Cohen EE, Mauer AM, et al. A phase I/II study of erlotinib and bevacizumab for recurrent or metastatic squamous cell carcinoma of the head and neck. ASCO Annual Meeting A5504, 2005.

62. E. Cohen, personal communication.

63. Seiwert TY, Haraf DJ, Cohen EE. A phase I study of bevacizumab with fluorouracil and hydroxyurea with concomitant radiotherapy for poor prognosis head and neck cancer. *Proc Am Soc Clin Oncol*. A5530, 287s, 2006.

64. Gaudreault J, Bruno R, Kabbinavar F, et al. Clinical pharmacokinetics of bevacizumab following every 2- or every 3-week dosing. ASCO Annual Meeting, A3041, 2004.

65. Miller KD, Chap LI, Holmes FA, et al. Randomized phase III trial of capecitabine compared with bevacizumab plus capecitabine in patients with previously treated metastatic breast cancer. *J Clin Oncol*. 23: 792-799, 2005.

66. Folkman J. What is the evidence that tumors are angiogenesis dependent? *J Natl Cancer Inst.* 3: 4-6, 1990.

67. Li CY, Shan S, Huang Q, et al. Initial stages of tumor-induced angiogenesis: Evaluation via skin window chambers in rodent models. *J Natl Cancer Inst*. 92: 143-147, 2000.

68. Folkman J, Kalluri R. Cancer without disease. *Nature*. 427: 787, 2004.

69. Hanahan D, Folkman J. Patterns and emerging mechanisms of the angiogenic switch during tumorigenesis. *Cell*. 86: 353-364, 1996.

REFERENCES (Continued)

70. Lo YM, Chan LY, Lo KW, et al. Quantitative analysis of cell-free Epstein-Barr virus DNA in plasma of patients with nasopharyngeal carcinoma. *Cancer Res*. 59: 1188-91, 1999.

71. Lo YM, Chan AT, Chan LY, et al. Molecular prognostication of nasopharyngeal carcinoma by quantitative analysis of circulating Epstein-Barr virus DNA. *Cancer Res*. 60: 6878-81, 2000.

72. Lo YM, Chan LY, Chan AT, et al. Quantitative and temporal correlation between circulating cell-free Epstein-Barr virus DNA and tumor recurrence in nasopharyngeal carcinoma. *Cancer Res*. 59: 5452-5, 1999.

73. Lin JC, Wang WY, Chen KY, et al. Quantification of plasma Epstein-Barr virus DNA in patients with advanced nasopharyngeal carcinoma. *NEJM*. 350: 2461-70, 2004.

74. Chan AT, Lo YM, Zee B, et al. Plasma Epstein-Barr virus DNA and residual disease after radiotherapy for undifferentiated nasopharyngeal carcinoma. *J Natl Cancer Inst*. 94: 1614-9, 2002.

75. Hong RL, Lin CY, Ting LL, Ko JY, Hsu MM. Comparison of clinical and molecular surveillance in patients with advanced nasopharyngeal carcinoma after primary therapy: the potential role of quantitative analysis of circulating Epstein-Barr virus DNA. *Cancer*. 100:1429-37, 2004.

76. Li YH, Shao JY, Zhao MQ, et al. Quantitative analysis of plasma Epstein-Barr virus (EBV) DNA for monitoring of recurrence and metastasis in nasopharyngeal carcinoma patients after radiotherapy. *Ai Zheng.* 22:645-8, 2003.

77. Leung SF, Tam JS, Chan ATC. Improved accuracy of detection of nasopharyngeal carcinoma by combined application of circulating epstein-barr virus DNA and anti-epstein-barr viral capsid antigen IgA antibody. *Clin Chem*. 339-345, 2004.

78. Chan AT, Ma BB, Lo YM, et al. Phase II study of neoadjuvant carboplatin and paclitaxel followed by radiotherapy and concurrent cisplatin in patients with locoregionally advanced nasopharyngeal carcinoma: Therapeutic monitoring with plasma Epstein-Barr virus DNA. *J Clin Oncol*. 22:3053-60, 2004.

79. Lo YM, Leung SF, Chan LY, et al. Kinetics of plasma Epstein-Barr virus DNA during radiation therapy for nasopharyngeal carcinoma. *Cancer Res*. 60: 2351-5, 2000.

80. Ngan RK, Lau WH, Yip TT, et al. Remarkable application of serum EBV EBER-1 in monitoring response of nasopharyngeal cancer patients to salvage chemotherapy. *Ann N Y Acad Sci* . 945:73-9, 2001.

81. Kondo S, Horikawa T, Takeshita H, et al. Diagnostic value of serum EBV-DNA quantification and antibody to viral capsid antigen in nasopharyngeal carcinoma patients. *Cancer Sci*. 95:508-13, 2004.

82. Fleming T. One-sample multiple testing procedure for phase II clinical trials. *Biometrics*. 38 :143-151, 1982.

83. Kalbfleisch JD, Prentice, RL. *The Statistical Analysis of Failure Time Data*. New York: John Wiley and Sons; 1980.

84. Kaplan EL, Meier P. Nonparametric estimation from incomplete observations. *J Amer Statist Assoc*. 53: 457-481, 1958.

APPENDIX I

Informed Consent Template for Cancer Treatment Trials (English Language)

RTOG 0615

A Phase II Study of Concurrent Chemoradiotherapy Using Three-Dimensional Conformal Radiotherapy (3D-CRT) or Intensity-Modulated Radiation Therapy (IMRT) + Bevacizumab (BV) [NSC 708865; IND 7921] for Locally or Regionally Advanced Nasopharyngeal Cancer

This is a clinical trial, a type of research study. Your study doctor will explain the clinical trial to you. Clinical trials include only people who choose to take part. Please take your time to make your decision about taking part. You may discuss your decision with your friends and family. You can also discuss it with your health care team. If you have any questions, you can ask your study doctor for more explanation.

You are being asked to take part in this study because you have nasopharyngeal cancer that has not spread to other parts of your body.

Why is this study being done?

The standard treatment for your tumor is radiation therapy and chemotherapy given together, followed by chemotherapy alone. The standard treatment can stop tumors from growing in the head and neck region in most patients. However, the cancer still can spread to other parts of the body. The drug, bevacizumab, may decrease the chance of the cancer spreading.

Tumors set off the growth of new blood vessels, which feed the tumor. This growth of new blood vessels is called angiogenesis. Bevacizumab is an anti-angiogenic agent, which interrupts your body's ability to grow new blood vessels, causing tumors to shrink. In 2004, the FDA approved bevacizumab for treatment of colorectal cancer when used with standard chemotherapy. Bevacizumab is an investigational treatment for nasopharyngeal cancer.

The purpose of this study is to determine whether adding bevacizumab to the standard treatment of radiation therapy and chemotherapy is safe and tolerable for you. This study will find out what effects, good and/or bad, radiation therapy, chemotherapy (cisplatin and 5-fluorouracil) and bevacizumab has on you and your cancer.

How many people will take part in the study?

About 46 people will take part in this study.

What will happen if I take part in this research study? (5/7/07)

If you take part in this study, you will receive treatment in 2 stages:

- **Stage 1**: You will receive a combination of radiation therapy, chemotherapy (cisplatin), and bevacizumab for about 6-7 weeks. During this stage:
  - You will receive radiation therapy once a day, Monday through Friday, for about 6 weeks.
  - You also will receive cisplatin and bevacizumab through your vein, once on the first day of radiation therapy then once every 3 weeks until radiation ends (a total of 3 treatments).
- **Stage 2:** About 3 weeks after completing Stage 1, you will receive a combination of chemotherapy (cisplatin and 5-fluorouracil) and bevacizumab through your vein. Then you will receive cisplatin, 5-fluorouracil, and bevacizumab once every 3 weeks for about 6 weeks (a total of 3 treatments).

You will receive treatment for a total of about 15-16 weeks.

Before you begin the study
You will need to have the following exams, tests or procedures to find out if you can be in the study. These exams, tests or procedures are part of regular cancer care and may be done even if you do not join the study. If you have had some of them recently, they may not need to be repeated. This will be up to your study doctor.

- A physical examination
- You will be weighed and asked about your ability to carry out your daily activities.
- You will be asked what medicines you are taking.
- An EKG, a test that measures the electrical activity of the heart on the surface of the chest
- A chest X-ray
- An MRI (Magnetic Resonance Imaging) of your tumor; an MRI is imaging using a strong magnetic field to look at one part of your body. Or a CT (Computed Tomography) scan of your tumor, if your study doctor recommends this test instead. A CT scan is a study using x-rays to look at one part of your body
- Blood tests (about 2-3 teaspoons of blood will be taken from your vein)
- Urine test
- A dental evaluation
- A hearing test
- For women able to have children, a pregnancy test
- And if your study doctor recommends:
  - The combination of a PET (Positron Emission Tomography) and CT scan of your body for planning your radiation treatment; A PET scan is a computerized image that looks at the activity of tumor cells in your entire body and that requires injection of a special marker into your vein, such as sugar (glucose) combined with a low-dose radioactive substance (a tracer). A camera records the tracer's signal as it travels through your body.
  - A CT scan of your liver
  - A bone scan, a type of x-ray to find out if cancer has spread to your bones
  - An evaluation of your diet and ability to chew and swallow; Some patients have difficulty chewing and swallowing during treatment and cannot take in enough food for their body's needs. You and the study doctor should discuss whether you should have a feeding tube placed before starting treatment.

During the study
If the exams, tests and procedures show that you can be in the study, and you choose to take part, then you will need the following tests and procedures. They are part of regular cancer care. The urine test and some of the blood tests are being done more often because you are in this study.

- A physical examination, taking your weight, evaluation of your ability to carry out your daily activities, and blood tests:
  - Weekly while you receive radiation, chemotherapy, and bevacizumab (during Stage 1)
  - When radiation, chemotherapy, and bevacizumab is completed (at end of Stage 1)
  - Prior to each chemotherapy and bevacizumab treatment (during Stage 2); some blood tests also will be done after chemotherapy to check the effect of treatment and to find out if treatment doses should be adjusted.
- A urine test every time you receive bevacizumab
- Taking your weight: Every 4 weeks while you receive chemotherapy and bevacizumab (during Stage 2)
- A dental evaluation at 3, 6, and 12 months following completion of radiation therapy
- If your study doctor recommends, a hearing test following completion of radiation therapy and then yearly

You will need these tests and procedures in follow-up visits. They are being done to see how you and your cancer was affected by the treatment you received. These tests and procedures are part of regular cancer care.

- An MRI of your tumor at 2 and 4 months after radiation therapy, every 6 months for years 1-2, then once a year
- A chest x-ray and blood tests every 6 months for years 1-3

In addition after you have finished treatment, the study doctor will ask you to visit the office for follow-up exams at the end of Stage 1, at the end of Stage 2, at 6, 9, and 12 months from the start of your treatment, every 3 months during year 2, every 6 months during years 3-5, then once a year.

Study Plan (5/7/07)

Another way to find out what will happen to you during the study is to read the chart below. Start reading at the top and read down the list, following the lines and arrows.

Stage 1 (About 6 weeks)
Radiation Therapy, Cisplatin, and Bevacizumab

Radiation, once a day, M-F, for about 6 weeks
Cisplatin and Bevacizumab once on the first day of radiation and then once every 3 weeks until radiation ends (a total of 3 treatments)

↓

Stage 2 (About 6 weeks)
Cisplatin, 5-Fluorouracil, and Bevacizumab

Cisplatin, 5-Fluorouracil, and Bevacizumab about 3 weeks after finishing Stage 1 and then Cisplatin, 5-Fluorouracil, and Bevacizumab once every 3 weeks for about 6 weeks (a total of 3 treatments)

How long will I be in the study? (5/7/07)

You will receive a combination of radiation therapy, chemotherapy, and bevacizumab for about 6 weeks. Then, you will receive chemotherapy and bevacizumab for 6 weeks. You will receive treatment for a total of about 15-16 weeks.

After you have finished treatment, the study doctor will ask you to visit the office for follow-up exams at the end of Stage 1, at the end of Stage 2, at 6, 9, 12 months from the start of your treatment, every 3 months during year 2, every 6 months during years 3-5, then once a year.

Can I stop being in the study?

Yes. You can decide to stop at any time. Tell the study doctor if you are thinking about stopping or decide to stop. He or she will tell you how to stop safely.

It is important to tell the study doctor if you are thinking about stopping so any risks from the radiation therapy, chemotherapy, or bevacizumab can be evaluated by him/her. Another reason to tell your study doctor that you are thinking about stopping is to discuss what follow-up care and testing could be most helpful for you.

The study doctor may stop you from taking part in this study at any time if he/she believes it is in your best interest; if you do not follow the study rules; or if the study is stopped.

What side effects or risks can I expect from being in the study?

You may have side effects while on the study. Everyone taking part in the study will be watched carefully for any side effects. However, researchers don't know all the side effects that may happen. Side effects may be mild or very serious. Your health care team may give you medicines to help lessen side effects. Many side effects go away soon after you stop taking the radiation therapy, chemotherapy, or bevacizumab. . In some cases, side

effects can be serious, long lasting, or may never go away. Death from treatment is possible, but rare (less than 5%).

You should talk to your study doctor about any side effects that you have while taking part in the study.

Risks Associated With Radiation

Very Likely
- Sores in the mouth and/or throat, which can be painful and make it very difficult to chew and/or swallow foods and for which most patients will need medicine for pain

Because chewing and/or swallowing are likely to be difficult, the study doctor may recommend placing a feeding tube before or during treatment so you can receive the nutrition you need. You might need a long term or permanent feeding tube. You should talk to your study doctor about this.

- Mouth dryness or changes in taste and/or smell that may be permanent
- Thick saliva
- Tanning or redness of the skin in the head and neck area being treated with radiation
- Ear pain and/or pressure
- Fatigue
- Weight loss
- Permanent hair loss in the area treated with radiation
- Loss of teeth, or cavities in the teeth, if strict dental care is not followed and/or hypersensitivity of teeth

Less Likely, But Serious
- Decrease in function of the thyroid gland that may require you to take thyroid replacement medicine to prevent you from feeling tired or sleepy
- Serious damage to the spinal cord, nerves in the neck, jawbone, voice box, skin, or other parts of the head and neck that may require a major operation to correct and, rarely, can even be life threatening
- Temporary pain or scarring around nerves in the shoulder that could cause numbness and/or weakness
- Breathing problems
- Difficulty with swallowing and eating with the possibility of inhaling food and/or liquids into the lungs – which could also result in pneumonia.
- Serious ear infections and/or hearing loss
- Damage to the spinal cord leading to permanent weakness and/or symptoms like a “stroke”
- Permanent hair loss (of the face/chin/neck)

Risks Associated with Chemotherapy

Cisplatin

Very Likely
- Decrease in blood counts, which can lead to a risk of infection, decreased healing after surgery, and/or bleeding
- Anemia
- Loss of appetite and/or taste; metallic taste in your mouth
- Nausea and/or vomiting
- Fatigue
- Generalized loss of strength
- Hearing loss, ringing in the ears
- Loss of muscle or nerve function that may cause weakness or numbness in your hands and feet
- Loss of appetite and weight loss
- Low magnesium in the body
- Low calcium in the body (It is unlikely that the calcium level will be low enough to affect heart function)
- Low potassium in the body(It is unlikely that the potassium level will be low enough to affect heart function)
- Kidney damage

Less Likely
- Allergic reactions (sweating, difficulty breathing, rapid heartbeat)

- Muscle cramps or spasm
- Facial swelling
- Loss of taste
- Loss of coordination
- Involuntary movement
- Restlessness
- Loss of hair, which is temporary
- Blood clots
- Low blood pressure

Less Likely, But Serious
- Seizures
- A severe allergic reaction, which could be life threatening
- Decrease in the kidneys' ability to handle the body's waste, which may be permanent
- Decrease in liver function
- Another cancer called acute leukemia
- A condition called hemolytic uremic syndrome that involves decreased red blood cells and platelets, fever, and kidney failure

5-FU (5-fluorouracil)

Likely
- Decrease in white blood cell count, which may increase the risk of infection, decreased healing, and/or bleeding
- Decrease in red blood cell count, which may result in anemia, tiredness, and/or shortness of breath
- Loss of appetite
- Nausea and/or vomiting
- Diarrhea with cramping or bleeding
- Skin rash
- Fatigue
- Headaches
- Hair loss, which is temporary
- Mouth sores
- Sore throat

Less Likely

- Confusion
- Eye irritation, watering of eyes, and/or runny nose
- Redness, tenderness, peeling, and/or tingling of the palms and soles of feet
- Increased sensitivity to sunlight
- Darkening of the skin, nails, or veins
- Loss of coordination or balance

Less Likely, But Serious
- Damage to the heart or spasm of the heart's blood vessels that can cause chest pain
- Inflammation of the liver, which may result in yellowing of skin and eyes, tiredness, and/or pain on upper right of the stomach area
- Infection at the catheter entry site

Risks Associated with Bevacizumab (4/21/08)

Very Likely:
- Nose bleeds
- High blood pressure - In most patients, blood pressure can be controlled with routine medications. Rarely, uncontrolled hypertension may lead to damage to the brain and other vital organ functions.
- Fatigue
- Rash
- Headache
- Soreness in mouth or throat

Less Likely

- Dizziness
- Decrease in blood counts, which can lead to a risk of infection
- Anemia
- Low blood pressure
- Loss of appetite
- Weight loss
- Itching, hives, welts of the skin
- Ulcers (open sores of the skin or mucus membrane that shed inflamed dead tissue)
- Nausea and/or vomiting
- Constipation
- Inflammation of the colon, which can result in stomach cramps and/or diarrhea
- Obstruction of the bowel
- Mild to moderate bleeding in the tumor, stomach, intestines, vagina, or other parts of the body
- Blood clots in the veins: blood clots can occur in the veins of the leg and the lungs or other organs. These events can be life-threatening.
- Clots in the arteries, including stroke or heart attack; these conditions can be life-threatening or fatal: When several studies were looked together, problems due to clots in arteries were increased about two-fold (up to 4-5%) in patients receiving chemotherapy plus bevacizumab compared to chemotherapy alone (about 2%). Elderly patients with past history of clots in the arteries were at a greater risk for these problems.
- Leakage of protein in the urine, which rarely can lead to damage to the kidney
- Reactions associated with infusion of the bevacizumab: rash, chills, fever, rigor
- Watery eyes, nasal stuffiness
- Shortness of breath, cough, wheezing
- Pain in the stomach, chest, joints or muscles and/or pain at the tumor site
- Hoarseness and/or change in or loss of voice

Rare but serious

- Serious or fatal bleeding from the tumor, brain, intestines, vagina, or the lungs
- Fistulas (abnormal openings or passages between internal organs or from an internal organ to the surface of the body) in the lung and/or intestinal tract
- Nasal septum perforation: a hole in the wall that divides the inside of the nose, which could result in crusting in the nose, bleeding and/or discharge from the nose, and/or whistling on intake of air and which would require surgery to repair
- Bowel perforation and bowel anastomotic dehiscence. Bowel perforation occurs when an opening exists in the bowel wall allowing bowel contents to spill into the abdomen. Bowel anastomotic dehiscence is a breakdown in the surgical connection between two pieces of bowel. These events are rare but can lead to serious infection and require surgery to repair.
- Heart problems (including irregular heartbeat, fluid collections surrounding the heart, heart attack or heart failure)
- Delayed or poor wound healing
- Acute and/or severe allergic reactions that result in difficulty breathing or drop in blood pressure
- Reversible Posterior Leukoencephalopathy Syndrome (RPLS) (<1%): RPLS is a medical condition related to leakiness of blood vessels in the brain and can cause confusion, blindness or vision changes, seizure and other symptoms, as well as changes in brain scans. This condition is usually reversible, but in rare cases, it is potentially life-threatening and may have long-term effect on the brain function.
- Reversible changes in liver functions
- Kidney failure
- Sudden death of uncertain relationship to bevacizumab

Risks Associated with Bevacizumab, Chemotherapy, and Radiation Therapy (5/7/07)
The combination of bevacizumab with chemotherapy and radiation therapy could increase the likelihood and/or severity of the side effects of chemotherapy and radiation therapy, such as the severity of mouth sores or risk of infection, bleeding, or anemia.

Low blood count (neutropenia) with increased risk of infection is a common side effect of chemotherapy drugs. This side effect may be increased when bevacizumab is added to chemotherapy. In some studies of bevacizumab plus chemotherapy, there also has been an increase in neutropenia-related fever and infections, including rare instances of infections leading to death.

Risks Associated with Feeding Tube Placement (if recommended by your doctor)
- Soreness at the site of the tube placement for a few days
- The area around the tube may become infected
- Bleeding may occur
- Rarely, stomach acid could leak onto the skin or into the abdomen, requiring an operation to revise the tube

Reproductive risks:
Because the drugs and radiation in this study can possibly affect an unborn baby and infants, you should not become pregnant or father a baby or breast feed while you are on this study. Also, because bevacizumab remains in your body for weeks to months, you should continue to use adequate contraceptive measures and avoid nursing a baby for at least 6 months after your last dose of bevacizumab, although the optimal or the maximal time required for drug clearance cannot be precisely predicted. Check with your study doctor about what kind of birth control methods to use and how long to use them. Some methods might not be approved for use in this study. In addition while, data in patients are not available, bevacizumab may have negative impact on fertility.

For more information about risks and side effects, ask your study doctor.

Are there benefits to taking part in the study?

Taking part in this study may or may not make your health better. While researchers hope that radiation therapy, chemotherapy, and bevacizumab will be more useful against cancer compared to the usual treatment, there is no proof of this yet. We do know that the information from this study will help researchers learn more about these therapies as a treatment for cancer. This information could help future cancer patients.

What other choices do I have if I do not take part in this study?

Your other choices may include:
- Radiation therapy given with chemotherapy but without bevacizumab
- Getting treatment or care for your cancer without being in a study
- Taking part in another study
- Getting no treatment
- Getting comfort care, also called palliative care. This type of care helps reduce pain, tiredness, appetite problems and other problems caused by the cancer. It does not treat the cancer directly, but instead tries to improve how you feel. Comfort care tries to keep you as active and comfortable as possible.

Talk to your study doctor about your choices before you decide if you will take part in this study.

Will my medical information be kept private?

Data are housed at RTOG Headquarters in a password-protected database. We will do our best to make sure that the personal information in your medical record will be kept private. However, we cannot guarantee total privacy. Your personal information may be given out if required by law. If information from this study is published or presented at scientific meetings, your name and other personal information will not be used.

Organizations that may look at and/or copy your medical records for research, quality assurance, and data analysis include:
- Radiation Therapy Oncology Group

- The National Cancer Institute (NCI) and other government agencies, like the Food and Drug Administration (FDA), involved in keeping research safe for people
- Genentech, manufacturer of bevacizumab

What are the costs of taking part in this study?

You and/or your health plan/ insurance company will need to pay for some or all of the costs of treating your cancer in this study. Some health plans will not pay these costs for people taking part in studies. Check with your health plan or insurance company to find out what they will pay for. Taking part in this study may or may not cost your insurance company more than the cost of getting regular cancer treatment.

Genentech is supplying bevacizumab at no cost to you while you are participating in the study. However, if you should need to take bevacizumab much longer than is usual, it is possible that the supply of free bevacizumab could run out. If this happens, your study doctor will discuss with you how to obtain additional drug from the manufacturer, and you may be asked to pay for it. In addition, you or your health plan may need to pay for costs of the supplies for drug administration and personnel who give you the bevacizumab.

You will not be paid for taking part in this study.

For more information on clinical trials and insurance coverage, you can visit the National Cancer Institute's Web site at http://cancer.gov/clinicaltrials/understanding/insurance-coverage. You can print a copy of the "Clinical Trials and Insurance Coverage" information from this Web site.

Another way to get the information is to call 1-800-4-CANCER (1-800-422-6237) and ask them to send you a free copy.

What happens if I am injured because I took part in this study?

It is important that you tell your study doctor, __________________ *[investigator's name(s)],* if you feel that you have been injured because of taking part in this study. You can tell the study doctor in person or call him/her at __________________ *[telephone number].*

You will get medical treatment if you are injured as a result of taking part in this study. You and/or your health plan will be charged for this treatment. The study will not pay for medical treatment.

What are my rights if I take part in this study?

Taking part in this study is your choice. You may choose either to take part or not to take part in the study. If you decide to take part in this study, you may leave the study at any time. No matter what decision you make, there will be no penalty to you and you will not lose any of your regular benefits. Leaving the study will not affect your medical care. You can still get your medical care from our institution.

We will tell you about new information or changes in the study that may affect your health or your willingness to continue in the study.

In the case of injury resulting from this study, you do not lose any of your legal rights to seek payment by signing this form.

Who can answer my questions about the study?

You can talk to your study doctor about any questions or concerns you have about this study. Contact your study doctor __________________ *[name(s)]* at __________________ *[telephone number].*

For questions about your rights while taking part in this study, call the ________________________ *[name of center]* Institutional Review Board (a group of people who review the research to protect your rights) at __________________ *(telephone number). [Note to Local Investigator: Contact information for patient representatives or other individuals in a local institution who are not on the IRB or research team but take calls regarding clinical trial questions can be listed here.]*

*You may also call the Operations Office of the NCI Central Institutional Review Board (CIRB) at 888-657-3711 (from the continental US only). *[*Only applies to sites using the CIRB.]*

Please note: This section of the informed consent form is about additional research that is being done with people who are taking part in the main study. You may take part in this additional research if you want to. You can still be a part of the main study even if you say 'no' to taking part in this additional research.

You can say "yes" or "no" to the following study. Below, please mark your choice.

Consent Form for Use of Tissue and Blood for Research

About Using Tissue and Blood for Research (5/7/07)

You are going to have or have had a biopsy (or surgery) to see if you have cancer. Your doctor will remove some body tissue to do some tests. The results of these tests will be given to you by your doctor and will be used to plan your care.

We would like to keep some of the tissue that is left over for future research. If you agree, this tissue will be kept and may be used in research to learn more about cancer and other diseases. Please read the information sheet called "How is Tissue Used for Research" to learn more about tissue research. This information sheet is available to all at the following web site: http://www.cancerdiagnosis.nci.nih.gov/specimens/patient.pdf

In addition, we would like to send a small amount of your blood to a central office for future research. About 4 teaspoons of your blood will be drawn 3 times: before treatment, 2 weeks after chemotherapy and bevacizumab (stage 2), 1 year after treatment. These samples of your blood will be sent to the central office and will be used to learn more about cancer and other diseases.

Your tissue and blood may be helpful for research whether you do or do not have cancer. The research that may be done with your tissue and blood is not designed specifically to help you. It might help people who have cancer and other diseases in the future.

Reports about research done with your tissue and blood will not be given to you or your doctor. These reports will not be put in your health record. The research will not have an effect on your care.

Things to Think About

The choice to let us keep your tissue and blood for future research is up to you. No matter what you decide to do, it will not affect your care or your participation in the main part of the study.

If you decide now that your tissue and blood can be kept for research, you can change your mind at any time. Just contact us and let us know that you do not want us to use your blood. Then any blood that remains will no longer be used for research and will be destroyed.

In the future, people who do research may need to know more about your health. While the doctor/institution may give them reports about your health, it will not give them your name, address, phone number, or any other information that will let the researchers know who you are.

Sometimes tissue and blood is used for genetic research (about diseases that are passed on in families). Even if your tissue and blood is used for this kind of research, the results will not be put in your health records.

Your tissue and blood will be used only for research and will not be sold. The research done with your tissue and blood may help to develop new products in the future.

Benefits

The benefits of research using tissue and blood include learning more about what causes cancer and other diseases, how to prevent them, and how to treat them.

Risks

The greatest risk to you is the release of information from your health records. We will do our best to make sure that your personal information will be kept private. The chance that this information will be given to someone else is very small.

Making Your Choice

Please read each sentence below and think about your choice. After reading each sentence, circle "Yes" or "No". If you have any questions, please talk to your doctor or nurse, or call our research review board at IRB's phone number.

No matter what you decide to do, it will not affect your care.

1. My tissue and blood may be kept for use in research to learn about, prevent, or treat cancer.

Yes No

2. My tissue and blood may be kept for use in research to learn about, prevent or treat other health problems (for example: diabetes, Alzheimer's disease, or heart disease).

Yes No

3. Someone may contact me in the future to ask me to take part in more research.

Yes No

Where can I get more information?

You may call the National Cancer Institute's Cancer Information Service at:

1-800-4-CANCER (1-800-422-6237) or **TTY: 1-800-332-8615**

You may also visit the NCI Web site at http://cancer.gov/

- **For NCI's clinical trials information, go to: http://cancer.gov/clinicaltrials/**
- **For NCI's general information about cancer, go to http://cancer.gov/cancerinfo/**

You will get a copy of this form. If you want more information about this study, ask your study doctor.

Signature

I have been given a copy of all _____ *[insert total of number of pages]* **pages of this form. I have read it or it has been read to me. I understand the information and have had my questions answered. I agree to take part in this study.**

Participant _________________________________

Date ______________________________________

<u>APPENDIX II</u> (4/21/08)

RTOG 0615 STUDY PARAMETER TABLE

Assessments	Pre-Treatment	Concurrent Phase	Adjuvant Phase	Follow up
**Evaluations**				
Biopsy	X			X (if indicated)
History/physical	X	X	X	X
Zubrod, weight	X	X	X	X
EKG	X			
Adverse event evaluation		X	X	X
List of current meds	X			
Dental eval	X		X	X
Audiogram	X		X (if indicated)	X (if indicated)
Nutritional eval	Highly recommended; not required			
**Imaging**				
Chest X-ray (or chest CT or PET/CT with contrast)	X			X
MRI Of nasopharynx, with contrast	X			X
Liver CT	Only if indicated; see Section 3.1			
Bone scan				
Planning PET/CT	Recommended; not required			
**Labs**				
CBC and diff	X	X	X	
Platelets; Hgb	X	X	X	
Serum creatinine and/or calc creatinine clearance	X	X	X	
UPC ratio	X	X	X	
Metabolic Panel	X	X	X	
Electrolytes, Mg++		X	X	
INR/aPTT	X			
TSH evaluation				X
Serum pregnancy test (if applicable)	X			
Tissue for banking	Highly recommended			
Blood for TRP	Highly recommended		Highly Recommended	Highly Recommended
Post-operative evaluation				X

See Section 11.2 for further details.

APPENDIX III

ZUBROD PERFORMANCE SCALE

0	Fully active, able to carry on all predisease activities without restriction (Karnofsky 90-100).
1	Restricted in physically strenuous activity but ambulatory and able to carry work of a light or sedentary nature. For example, light housework, office work (Karnofsky 70-80).
2	Ambulatory and capable of all self-care but unable to carry out any work activities. Up and about more than 50% of waking hours (Karnofsky 50-60).
3	Capable of only limited self-care, confined to bed or chair 50% or more of waking hours (Karnofsky 30-40).
4	Completely disabled. Cannot carry on self-care. Totally confined to bed or (Karnofsky 10-20).
5	Death (Karnofsky 0).

KARNOFSKY PERFORMANCE SCALE

100	Normal; no complaints; no evidence of disease
90	Able to carry on normal activity; minor signs or symptoms of disease
80	Normal activity with effort; some sign or symptoms of disease
70	Cares for self; unable to carry on normal activity or do active work
60	Requires occasional assistance, but is able to care for most personal needs
50	Requires considerable assistance and frequent medical care
40	Disabled; requires special care and assistance
30	Severely disabled; hospitalization is indicated, although death not imminent
20	Very sick; hospitalization necessary; active support treatment is necessary
10	Moribund; fatal processes progressing rapidly
0	Dead

APPENDIX IV

HEAD & NECK, 6th Edition

STAGING-PRIMARY TUMOR (T)

TX	Primary tumor cannot be assessed
T0	No evidence of primary tumor
Tis	Carcinoma *in situ*

Nasopharynx

T1	Tumor confined to the nasopharynx
T2	Tumor extends to soft tissues of oropharynx and or nasal fossa
T2a	without parapharyngeal extension
T2b	with parapharyngeal extension
T3	Tumor invades bony structures and/or paranasal sinuses
T4	Tumor with intracranial extension and/or involvement of cranial nerves, infratemporal fossa, hypopharynx, orbit, or masticator space

REGIONAL LYMPH NODES (N) Nasopharynx

NX	Regional lymph nodes cannot be assessed
N0	No regional lymph node metastasis
N1	Unilateral metastasis in lymph node(s), 6 cm or less in greatest dimension, above supraclavicular fossa*
N2	Bilateral metastasis in lymph node(s), 6 cm or less in greatest dimension, above supraclavicular fossa*
N3	Metastasis in lymph node(s) > 6 cm and/or to supraclavicular fossa
N3a	Greater than 6 cm in dimension
N3b	Extension to the supraclavicular fossa**

*Note: Midline nodes are considered ipsilateral nodes.

**Supraclavicular zone or fossa is relevant to the staging of nasopharyngeal carcinoma and is the triangular region originally described by Ho. It is defined by three points: 1) the superior margin of the sternal end of the clavicle; 2) the superior margin of the lateral end of the clavicle; 3) the point where the neck meets the shoulder. Note that this would include caudal portions of Levels IV and V. All cases with lymph nodes (whole or part) in the fossa are considered N3b.

DISTANT METASTASIS (M)

MX	Distant metastasis cannot be assessed
M0	No distant metastasis
M1	Distant metastasis

STAGE GROUPING Nasopharynx

Stage 0	T*is*, N0, M0
Stage I	T1, N0, M0
Stage IIA	T2a, N0, M0
Stage IIB	T1-T2a, N1, M0
	T2b, N0-1, M0
Stage III	T1-T2b, N2, M0
	T3, N0-2, M0
Stage IVA	T4, N0-2, M0
Stage IVB	Any T, N3, M0
Stage IVC	Any T, Any N, M1

APPENDIX V

MANAGEMENT OF DENTAL PROBLEMS IN IRRADIATED PATIENTS

Dental Care for Irradiated Patients
Goals for a dental care program include:
1. To reduce incidence of bone necrosis.
2. To reduce incidence of irradiation caries.
3. To allow proper fitting of dentures following treatment.

Pre-irradiation Care and Procedures
The patients may be grouped into four groups in accordance with the problems they present prior to irradiation.

Group 1
Includes edentulous patients. They may require surgical removal of any symptomatic cysts, infected retained root tips, or alveolar hyperplasia. These patients require hygiene instruction and precautionary instruction about trauma with premature use of a prosthesis.

Group 2
Includes those with poor dental hygiene, including those patients whose teeth are beyond repair by ordinary dental procedure, those with generalized oral sepsis, those with generalized periodontal disease, and those with chronic periapical abscesses or granulomas.

Procedures performed on this group include removal of all remaining teeth prior to irradiation with primary closure and surgical preparation of the alveolar ridges to laterally support a prosthesis. There should be antibiotic coverage during the healing stage and adequate time prior to the start of radiation therapy. These patients need complete hygiene instruction and precautionary instruction about premature use of a prosthesis.

Group 3
Includes those in whom dental condition is fair, including those patients whose teeth are restored, ordinary dental procedures, periodontal pockets are less than 3 mm deep, carious lesions are not in proximity to the pulp, and no more than 20 restorable carious lesions are present. X-ray examinations show at least 1/2 of the bone still present around root surfaces. These patients require removal of any teeth that are non-salvageable in accordance with the above and restorations of the remaining teeth as required. The patients are instructed for dental prophylaxis and the patients utilize custom-made fluoride carriers.

Group 4
Includes those in whom dental hygiene is good. This includes patients who do not have severe malocclusion in whom few carious lesions are present. Carious lesions are not in close proximity to the pulp and are correctable by conventional methods. These patients require periodontal evaluation and dental prophylaxis training, restorations as needed, no extractions prior to radiation therapy, and fitting for custom carriers.

Extraction of Teeth
If extraction of teeth is necessary prior to radiation therapy, the bone must be contoured so that closure at the extraction site is possible. All loose spicules and sharp projections must be removed. The approximation of the gingival tissue must be such that the closure is neither too loose nor too tight. At least 10 days are required for adequate healing prior to initiation of therapy.

Causative Factors
The major causative factors appear to be the reduction of the amount of saliva and secondarily, reduced pH in the mouth. This occurs following high dose radiation to the major salivary glands using parallel opposed fields. The decay process usually occurs in the first year following radiation therapy. It tends to occur more quickly in teeth which have a large amount of root cementum exposed to those teeth with large amounts of plaque formation present. Doses of radiation in excess of 20 Gy to salivary tissue place the teeth at risk.

Preventive Program
The rationale behind the use of fluoride treatments is to make the tooth surfaces less susceptible to the decay process. This is accomplished by a combination of increasing fluoride concentration on the tooth surface and by the effect of fluoride on the plaque and flora that are present in the oral cavity. Adequate results are obtained by: 1) cleansing the teeth thoroughly, followed by a good home care dental prophylaxis program, 2) construction of

fluoride carriers, custom-made mouth guards, which provide local application of fluoride solution to the gingiva and tooth surfaces. Fluoride carriers are made individually with the use of casts. Material used for making a mouth guard is "Sta-Guard" plastic used in conjunction with vacutrole unit produced by Jelrus Technical Products, Corp., both of which are available through local dental supply. This material is molded to the cast impression and allowed to harden. A fluoride solution prepared at the M.D. Anderson Hospital is now available from the Emerson Laboratories, Inc., Dallas, Texas 75221. It has been used to coat the plastic carrier for use in the mouth. The patients are instructed to cleanse their teeth prior to placement of the carrier. It is then worn in place for 5 minutes each day. The patients are instructed to rinse their mouths thoroughly following the use of the carrier. This will be continued for an indefinite period of time. Close follow-up is necessary.

Results
In the 5-1/2 year program at the M.D. Anderson Hospital beginning in 1966, a study of 304 patients shows that the incidence of necrosis of the jaw was reduced to approximately 21% compared to 37% prior to initiation of the study. Groups 3 and 4 patients randomized with and without fluoride treatment showed reduction in radiation carries from 67% to 34% among Group 3 patients, and from 65% to 22% among Group 4 patients.

Failure to Control Decay
Management of failure to control radiation decay includes silver fillings with continued use of fluoride treatments. If the decay process is sufficiently advanced that a filling will no longer stay in place, these teeth are merely smoothed so that there will be no sharp, irritating edges. The mere existence of such a decayed tooth is not necessarily reason for extraction, for it must be remembered that extraction could lead to complications such as bone necrosis.

Pulp exposure resulting from the decay process can usually be handled by use of antibiotics and/or root-canal therapy.

Hypersensitivity of Teeth
Occasionally, a patient will exhibit extreme sensitivity of the teeth secondary to diminished amounts of saliva. This has been shown to be reduced in incidence with the fluoride treatments. Should this problem become manifest, increasing the fluoride treatment to 10 to 15 minutes 3 times a day is recommended.

Infections
Infections occurring in patients under or after radiation therapy are best managed conservatively with good oral hygiene, irrigation and flushing procedures, and systemic antibiotics.

Bone Necrosis
The patients receiving radiation therapy to a high dose to the head and neck region have increased susceptibility to bone necrosis for several reasons including: impairment of normal metabolism, increased susceptibility to infection and severely limited repair process. Bone necrosis occurs most often after post-irradiation surgery or other traumas. Conservative management should be tried first, though in more aggressive lesions a more radical approach may ultimately be necessary.

Appendix VI (4/21/08)
SPECIMEN PLUG KIT*

The Specimen Plug Kit contains a shipping tube and a dermal needle. **Note**: **Sites should not dispose of the Plug Kit.** Sites should ship the Plug Kit to the RTOG Biospecimen Resource to be used again.

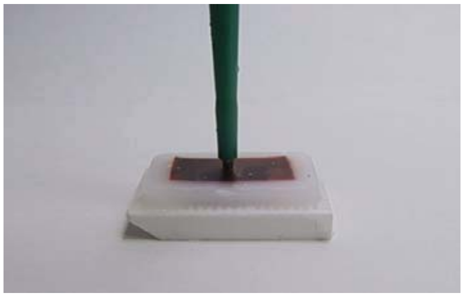

Step 1
Place the dermal needle on the paraffin block over the selected tumor area. (*Ask a Pathologist to select area with tumor.*) Push the needle into the paraffin block. Twist the needle once around to separate the plug from the block. Then pull the needle out of the block. The needle should be filled with tissue sample.

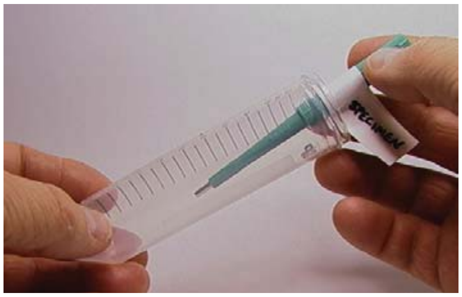

Step 2
Label dermal needle with proper specimen ID. **Do not try to remove specimen from needle.**

Use a separate dermal needle for every specimen. **Do not mix specimens.** Call or email the RTOG Biospecimen Resource for questions or for additional specimen Plug Kits.

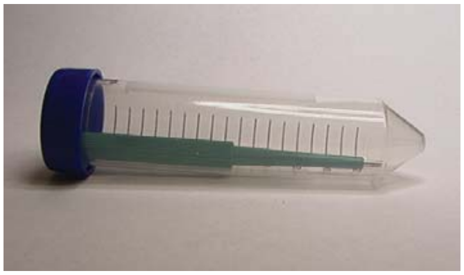

Step 3
Once specimen needle is labeled, place it in the shipping tube and mail to the address below.

The RTOG Biospecimen Resource will remove the specimen from the needle and embed it in a cassette, labeled with the specimen ID.

***NOTE**: If an institution is uncomfortable obtaining the plug but wants to retain the tissue block, the institution should send the entire block to the RTOG Biospecimen Resource. The Biospecimen Resource will sample a plug from the block and will return the remaining block to the institution. Institutions should indicate their request to perform the plug procedure and to return the block on the submission form.

Ship: Specimen Plug Kit, specimen in dermal needle, and all paper work as follows:

Mailing Address: For Non-frozen Specimens Only
RTOG Biospecimen Resource
University of California San Francisco
Campus Box 1800
1657 Scott Street, Room 223
San Francisco, CA 94143-1800

Courier Address (FedEx, DHL, etc.): For Frozen Specimens
RTOG Biospecimen Resource
University of California San Francisco
1657 Scott Street, Room 223
San Francisco, CA 94115

Questions: 415-476-RTOG (7864)/FAX 415-476-5271; RTOG@ucsf.edu

APPENDIX VII (4/21/08)
Blood Collection Kit Instructions

Study collection kits are available free of charge. Contact the RTOG Biospecimen Resource (contact information is below).

The kit includes:
- Ten (10) 1ml cryovials
- Biohazard bags
- Absorbent shipping material
- Styrofoam container (inner)
- Cardboard shipping (outer) box
- Pre-paid shipping label(s)

Instructions for collection of serum:
Using four (4) or more 1ml cryovials, label them with the RTOG study and case number, collection date and time, and clearly mark cryovials "serum".

Process:
1. Allow one 5ml red top tube to clot for 30 minutes at room temperature.
2. Spin in a standard clinical centrifuge at ~2500 RPM at 4° Celsius for 10 minutes.
3. Aliquot a minimum of 0.5 ml serum (optimal 1ml) into each cryovial labeled with RTOG study and case numbers, collection date/time, time point collected, and clearly mark specimen as "serum".
4. Place cryovials into biohazard bag and store serum at –80° Celsius until ready to ship.

Instructions for collection of plasma:
1. Collect 3 mL venous blood into an EDTA tube.
2. Put on ice or store at 4° C for at most 4 hours before processing
3. Centrifuge at 1,600 g for 10 minutes.
4. Carefully pipette out the supernatant without disturbing the blood cells.
5. Put the plasma into a 1.5 mL eppendorf tube.
6. Centrifuge at 16,000 g fo 10 minutes to remove any residual cells.
7. Pipette out the cell-free plasma, and put it into a clean eppendorf tube. Label with RTOG study and case numbers, collection date/time, time point collected, and clearly mark specimen as "plasma".
8. Place cryovials into biohazard bag and store serum at –80° Celsius until ready to ship.

PLEASE MAKE SURE THAT EVERY SPECIMEN IS LABELED.

Shipping/Mailing:
- Include all RTOG paperwork in pocket of biohazard bag.
- Ship specimens overnight Monday-Thursday. Avoid shipping on a weekend or around a holiday.
- Place frozen specimens and the absorbent shipping material in the Styrofoam cooler and fill with dry ice (if appropriate; double-check temperature sample shipping temperature). Ship ambient specimens in a separate envelope/cooler. Place Styrofoam coolers into outer cardboard box, and attach shipping label to outer cardboard box.
- *Multiple cases may be shipped in the same cooler, but make sure each one is in a separate bag.*
- Ship specimens as follows:

Mailing Address: For Non-frozen Specimens Only
RTOG Biospecimen Resource
University of California San Francisco
Campus Box 1800
1657 Scott Street, Room 223
San Francisco, CA 94143-1800

Courier Address (FedEx, DHL, etc.): For Frozen Specimens
RTOG Biospecimen Resource
University of California San Francisco
1657 Scott Street, Room 223
San Francisco, CA 94115

Questions: 415-476-RTOG (7864)/FAX 415-476-5271; RTOG@ucsf.edu
