# Supplementary material for: Long-term Outcomes of Bevacizumab and Chemoradiation for Locoregionally Advanced Nasopharyngeal Carcinoma: A Nonrandomized Controlled Trial
Source: JAMA Netw Open. 2023 Jun 2;6(6):e2316094. doi: 10.1001/jamanetworkopen.2023.16094 (PMC10238946; doi:10.1001/jamanetworkopen.2023.16094)
Supplement: Supplement 2. — Data Sharing Statement [file jamanetwopen-e2316094-s002.pdf]

Data Sharing Statement

Lee. Long-term Outcomes of Bevacizumab and Chemoradiation for Locoregionally Advanced Nasopharyngeal Carcinoma: A Nonrandomized Controlled Trial. *JAMA Netw Open*. Published online June 2, 2023. doi:10.1001/jamanetworkopen.2023.16094

Data

Data available: Yes
Data types: Deidentified participant data
How to access data: All deidentified participant data and data dictionary from this paper will be available upon request within 6 months of publication in accordance with NRG Oncology's data sharing policy, which can be found at https://www.nrgoncology.org/Resources/Ancillary-Projects-Data- Sharing-Application.
When available: With publication

Supporting Documents

Document types: None

Additional Information

Who can access the data: All deidentified participant data and data dictionary from this paper will be available upon request within 6 months of publication in accordance with NRG Oncology's data sharing policy, which can be found at https://www.nrgoncology.org/Resources/Ancillary-Projects-Data- Sharing-Application.
Types of analyses: All deidentified participant data and data dictionary from this paper will be available upon request within 6 months of publication in accordance with NRG Oncology's data sharing policy, which can be found at https://www.nrgoncology.org/Resources/Ancillary-Projects-Data- Sharing-Application.
Mechanisms of data availability: All deidentified participant data and data dictionary from this paper will be available upon request within 6 months of publication in accordance with NRG Oncology's data sharing policy, which can be found at https://www.nrgoncology.org/Resources/Ancillary-Projects-Data- Sharing-Application.
Any additional restrictions: All deidentified participant data and data dictionary from this paper will be available upon request within 6 months of publication in accordance with NRG Oncology's data sharing policy, which can be found at https://www.nrgoncology.org/Resources/Ancillary-Projects-Data- Sharing-Application.

© 2023 Lee NY et al. *JAMA Network Open*.
